# When Do Single‐Species Occupancy Models Outperform Multispecies Models?

**DOI:** 10.1002/ece3.72315

**Published:** 2025-11-23

**Authors:** Gavin G. Cotterill, Douglas A. Keinath, Tabitha A. Graves

**Affiliations:** ^1^ U.S. Geological Survey Northern Rocky Mountain Science Center West Glacier Montana USA; ^2^ U.S. Fish and Wildlife Service Wyoming Ecological Services Field Office Cheyenne Wyoming USA

**Keywords:** eDNA, guilds, habitat restoration, hierarchical community modeling, imperfect detection, JAGS, pollinator conservation, power analysis, presence/absence data, species exchangeability assumption

## Abstract

Occupancy models have become increasingly popular for species monitoring and assessment, in part, because detection/non‐detection data are readily obtained using a variety of methods. Multispecies occupancy models (MSOMs) can yield more accurate parameter estimates than single‐species models (SSOMs) with less data through their hierarchical structure, making MSOMs an attractive option when species are hard to detect or when data collection is constrained, leading to sparse datasets. Such constraints may arise from limited sampling resources, but also occur in rare species monitoring or where preliminary results are desired to inform adaptive management. Further, experimental habitat treatments often impose spatial constraints on sampling based on the scale of their implementation. Whether a MSOM outperforms SSOMs depends on the volume of data, characteristics of the ecological community, research goals of a study and how these factors align with modeling assumptions. We performed a simulation study of hypothetical pollinator communities under varying sampling intensities for scenarios in which experimental habitat treatments produced different community‐level effects. We fit occupancy models to simulated datasets and assessed model performance. At lower sampling intensities (< 20 spatial replicates and < 4 temporal replicates), MSOM community‐level treatment effect estimates were biased. Even at twice this sampling intensity, SSOMs yielded more accurate species‐specific effect estimates in treatment effect scenarios with high variance. In some cases, MSOMs can pull species in the tails of distributions too far toward the community mean effect, which risks incorrect conclusions concerning whether treatments help or harm individual species. When quantifying species‐specific effects is the main objective, particularly for rarely observed species, SSOMs are more robust to outliers across a range of community response scenarios. Researchers can use this information to inform study design, guide simulation studies and decide whether the higher precision of MSOMs outweighs risks of improperly estimated effects for some species.

## Graphical abstract

Plains prickly pear ( *Opuntia polyacantha* ) flower being pollinated by multiple members of the sweat bee family (Halictidae) in eastern Montana, USA. When species‐specific effects are the main research question of interest, particularly for rare species, single species occupancy models are more robust than multispecies models to outliers across a range of community response scenarios. Photograph by Erica Gustilo, U.S. Geological Survey, 2024.

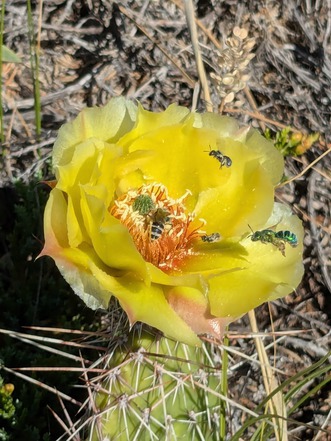

## 1 Introduction

Evaluating the effects of environmental covariates on species' abundance and distribution is the central goal of many studies. Often the choice of analytic approach is determined, at least in part, by the data that can be collected. Occupancy modeling has emerged as a popular tool for biological monitoring programs and species assessments (McGowan et al. 2020; Muths et al. 2005; Otto et al. 2025) because it can operate on binary species detection data (sometimes called presence/absence data), which can be collected using a variety of sampling methods (e.g., human observer, camera traps, eDNA) and are often easier to obtain than, for example, accurate abundance measures (MacKenzie and Nichols 2004). A major caveat with this data type is that failure to detect a species during a survey could arise either because that species is truly absent from a site or because it was missed (Tyre et al. 2003). For this reason, we use the terminology “detection/non‐detection” data. Ignoring imperfect detection could result in inferential consequences (Guillera‐Arroita et al. 2014; Lahoz‐Monfort et al. 2014) that can be resolved by jointly modeling observational (data collection) and state (the underlying biological truth) processes to directly estimate detection probabilities through repeat sampling of at least some sites (MacKenzie et al. 2002; Royle and Dorazio 2006). Originally formulated as single‐season, single‐species occupancy models (SSOMs), occupancy models have been expanded to include multi‐season (MacKenzie et al. 2003), multi‐species (Dorazio and Royle 2005), spatially explicit (Gelfand et al. 2005) and data‐integration extensions (Doser et al. 2021) to address different research questions.

A perceived weakness of SSOMs occurs when focal species are rare or difficult to detect, in which case acquiring sufficient observations for statistical modeling necessitates higher levels of sampling intensity (Guillera‐Arroita et al. 2010). The hierarchical multispecies occupancy models (MSOMs) ameliorate this dilemma by simultaneously considering two or more species. This is achieved by ascribing individual species to larger groups, as in individual species or functional groups within an ecological community (Dorazio and Royle 2005). In doing so, the precision and accuracy of parameter estimates for hard‐to‐detect species are improved by sharing information (i.e., partial pooling, also referred to as “borrowing strength”; Gelman and Hill 2007) with more frequently observed species, thus reducing the overall level of required sampling (Zipkin et al. 2009). MSOMs may be appealing because although collecting data on multiple species can create additional work, in practice, it may not increase project costs (e.g., bird point counts; eDNA samples of water, air, or flowers; vegetation transects) and can provide community information that may also be of interest.

Even though the implementation of occupancy models has never been easier, study design considerations remain far from trivial. Among simpler occupancy models, like SSOMs, providing broadly applicable sampling recommendations is difficult as sampling design must be carefully tailored to the particular system, objectives of a study and modeling assumptions (MacKenzie et al. 2017). Among more complex extensions, the task becomes more challenging. Whereas some occupancy model extensions seek to relax or accommodate possible assumption violations (e.g., explicitly handling lack of independence in spatial replicates), others, including MSOMs, rely on an expanding set of assumptions. For a concise summary of MSOM assumptions, refer to Devarajan et al. (2020). Thus, prescriptive advice for sampling design typically emphasizes the importance of clearly articulated study objectives: “why” the study will be conducted, “what” state variables are appropriate to measure and only then, “how” to sample and evaluate survey design tradeoffs, which may ultimately require a simulation study (Bailey et al. 2007; Block et al. 2001; Devarajan et al. 2020; Guillera‐Arroita et al. 2019; Pollock et al. 2002). Our motivations for the present study follow directly from this advice. In particular, we sought to explore sampling design relevant to pilot studies and the adaptive management of species of conservation concern under resource constraint limitations common among public land agencies (Moore et al. 2011; Smiley 2008). We therefore focus especially on considerations pertaining to (1) experimental habitat modifications, (2) limited sampling intensity and (3) understudied ecological communities.

Many occupancy studies are observational or descriptive in that spatial sampling replicates do not consist of discrete experimental and control sites (Eberhardt and Thomas 1991). Although the lack of experimental design has been leveled as a critique of ecological research (Hurlbert 1984) and can impose statistical and logistical challenges (Oksanen 2001), a key advantage is that large spatial datasets can be considered, thus permitting continental‐scale studies that reveal important aspects of a focal species' life history (e.g., Janousek et al. 2023; Pease et al. 2022). By contrast, land managers may implement habitat manipulations over relatively small spatial scales owing to high input costs associated with management actions and the size of managed parcels. In this context, the possible number of spatial replicates is restricted by logistics involving the scope of managers' jurisdiction, funding constraints on personnel hours and the need to locate spatial replicates at an appropriate distance from one another—locating sample sites too closely to one another can induce strong spatial autocorrelation, which, if unaccounted for, violates independence assumptions of the model (Legendre 1993). On the other hand, co‐locating sites can be an asset in paired treatment‐control designs if proximally located “site pairs” are selected such that their primary difference is the experimental treatment. Limited spatial replication can, to some extent, be alleviated by increased temporal replication (Bailey et al. 2007), yet repeat visits must occur within an appropriate time window to meet demographic closure assumptions for the surveyed taxa (Rota et al. 2009). Thus, an increased reliance on temporal replicates of sites that are potentially difficult to access can impose challenges even for less labor‐intensive detectors (e.g., cameras, bowl traps).

Managers may wish to evaluate the impacts of specific actions or practices on focal species; however, management actions are often designed to meet multiple objectives and may precede the interest in a focal species. Consequently, baseline data salient to focal species research may be absent. When the management actions to be evaluated, the focal species, or both, are relatively understudied, determining the species to be included in the modeled community for MSOMs requires additional thought. Correctly identifying group membership is a core challenge in statistical inference and ecology (Draper et al. 1993; Elton 1946; Hubbell 2005). In some contexts, defining a community based on the co‐occurrence of species may be reasonable. Importantly for evaluating management actions using MSOMs, ideal species groupings would be based on species' similarities of responses (Pacifici et al. 2014), and whether they can be modeled as arising from shared prior distributions—i.e., whether or not they should be treated as exchangeable (Bernardo 1996; Kéry and Royle 2015). Assuming exchangeability is often necessary absent better information (Gelman et al. 2013), but this assumption is not inconsequential, particularly when species‐specific inference (rather than community average responses) is the goal (Guillera‐Arroita et al. 2019; Pacifici et al. 2014; Sollmann et al. 2021).

**FIGURE 1:**
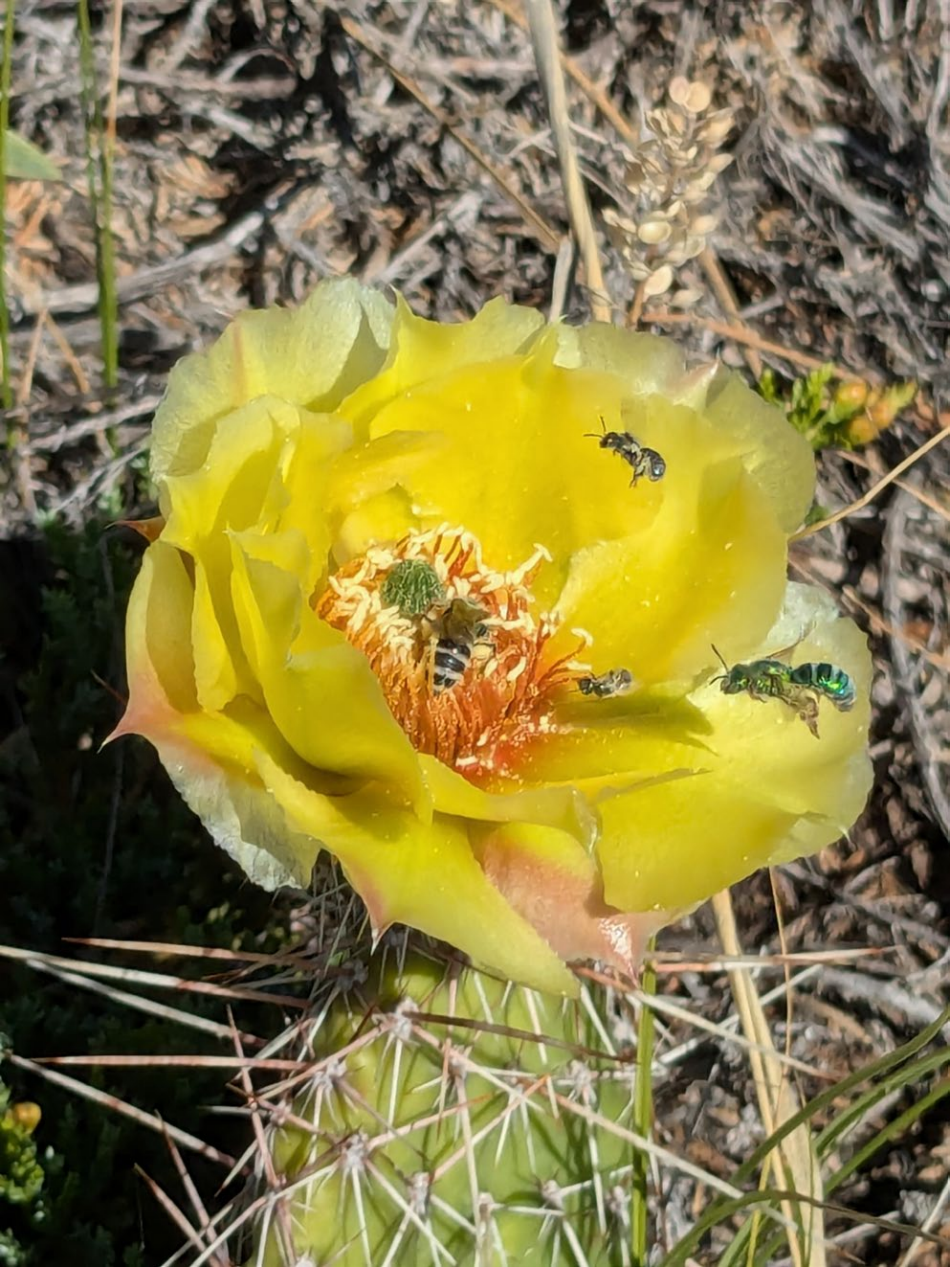
Plains prickly pear ( *Opuntia polyacantha* ) flower being pollinated by multiple members of the sweat bee family (*Halictidae*) in eastern Montana, USA. Photograph by Erica Gustilo, U.S. Geological Survey, 2024.

With these challenges in mind, we considered a hypothetical pollinator community in the western United States where native pollinators are declining due to complex causes including loss of floral resources (Ogilvie et al. 2017), increasing temperature and drought and neonicotinoid pesticide use (Janousek et al. 2023; Figure 1). Although directly addressing some factors may fall outside land managers' purview, improving habitat quality can sometimes help mitigate stressors. Some experimental treatments currently implemented on public lands in the region are expected to benefit all species in the pollinator community—e.g., restoration plantings to increase native flowers (Drobney et al. 2020)—whereas other treatments are expected to benefit some species at the expense of others—e.g., rotational livestock grazing that enhances ground‐nesting bee habitat but reduces bumble bee (*Bombus* spp.) nesting habitat (Goosey et al. 2024). We conducted a simulation study to identify minimum data requirements to accurately quantify species‐specific treatment effects using single‐season detection/non‐detection data. We hypothesized that actions producing highly variable responses, including scenarios in which some species benefit and others are harmed, would increase MSOM data requirements and reduce the accuracy of species‐specific estimates. We compared MSOM results against SSOM results using several error metrics. Our goal was to inform sample designs for evaluating habitat management actions that could be applied regardless of the taxa of interest or data collection method.

## 2 Materials and Methods

We analyzed treatment effects using occupancy models that consider a predetermined number of species in a community, rather than data‐augmented models (Dorazio and Royle 2005). This choice was made for several practical reasons: (1) Our focus was not on estimating species richness or biodiversity metrics; and (2) pollinator sampling often includes visually or genetically similar species, which restricts the depth of taxonomic identification. This leads to a mix of species‐, genus‐, or family‐level identifications due to bottlenecks in taxonomic expertise, methodological precision and cost (Woodard et al. 2020). These limitations can occur across sampling methods (e.g., human surveyors, bowl traps, or eDNA). Understanding how various species or functional groups respond might be more important than biodiversity metrics when management objectives include monitoring species of conservation concern.

### 2.1 Model Formulations

The multispecies occupancy model (Dorazio and Royle 2005) treats the latent occupancy (i.e., unobserved ecological truth or state process; *z*) at site *i*, for species *k*, as a Bernoulli random variable based on the probability, $\psi_{\mathrm{ik}}$,

$$z_{\mathrm{ik}}\sim \text{Bernoulli}\left(\psi_{\mathrm{ik}}\right)$$

$$\text{logit}\left(\psi_{\mathrm{ik}}\right)\sim \beta_{0k}+\beta_{1\ldots n,k}*X_{i}$$

where $\psi_{\mathrm{ik}}$ is logit‐transformed to accommodate a linear model estimating a random effect of species identity (the intercepts, $\beta_{0k}$) and one or more environmental effects ($\beta_{1\ldots n,k}$) from a vector of site‐specific covariates ($X_{i}$). This process model is linked to an observation model that estimates species' detection probabilities ($p_{k}$). In the MSOM, a data point ($y_{\mathrm{ijk}}$; i.e., whether species *k* was observed at site *i* during sampling event *j*) is a Bernoulli random variable based on probability $\theta_{\mathrm{ijk}}$, which is the probability of observing the species conditional on its occupying the site and its probability of detection:

$$y_{\mathrm{ijk}}\sim \text{Bernoulli}\left(\theta_{\mathrm{ijk}}\right)$$

$$\theta_{\mathrm{ijk}}=z_{\mathrm{ik}}*p_{\mathrm{ijk}}$$

$$\text{logit}\left(p_{\mathrm{ijk}}\right)\sim \alpha_{0k}$$

Detection probabilities can vary as a function of site or survey event (implied by the inclusion of three subscripts in the equation above), but here we simulated from, and subsequently fit, an intercept‐only ($\alpha_{0k}$) model in which detection probabilities only varied by species. This assumes no unmodeled heterogeneity across surveys, such as when survey protocols are designed to minimize weather and seasonal differences influencing species' abundance and detection. This is a common practice for pollinator surveys (FWS 2019), reduces the dimensionality of the simulations and speaks to the question of minimum data collection needs (if surveying during conditions that reduce detection rates, more data may be required). In our process model, we considered a single habitat covariate: that of categorical treatment effect. In this case, $X_{i}$ represents a “dummy variable”, the vector of zeros or ones indicating whether a site belonged to the control (0) or treatment (1) group. On their own, the $\alpha_{0k}$, $\beta_{0k}$ and $\beta_{1k}$ parameters are of interest, but our main goal was to estimate the change in species‐specific occupancy probabilities resulting from a treatment effect ($\hat{\lambda_{k}}$), which were the derived quantities,

$$\hat{\lambda_{k}}=\text{ilogit}\;\left(\beta_{0k}+\beta_{1k}\right)-\text{ilogit}\;\left(\beta_{0k}\right)$$

where ilogit is the inverse logit function $\frac{1}{\left(1+e^{-x}\right)}$.

This MSOM uses priors for species‐level random effects as required by Bayesian models (Kéry and Royle 2015). In ecological research, priors are usually uninformative or weakly informative distributions (Northrup and Gerber 2018). For example, priors for $\alpha_{0k}$, $\beta_{0k}$ and $\beta_{1k}$ could all be normal distributions with a zero mean and some error. Doing so is equivalent to fitting many single‐species occupancy models simultaneously. The difference in the MSOM is that priors for $\alpha_{0k}$, $\beta_{0k}$ and $\beta_{1k}$ are drawn from community‐level distributions for each of the parameters (called hyperpriors). The prior structure for a single parameter therefore took the form:

$$\beta_{0k}\sim \text{Normal}\left(\mu ,\tau \right)$$

$$\mu \sim \text{Normal}\left(0,2.25^{-2}\right)$$

$$\sigma \sim \text{halfCauchy}\left(2\right)$$

$$\tau =\sigma^{-2}$$

Here, species‐specific occupancy intercepts ($\beta_{0k}$) were drawn from normal distributions governed by the community‐level mean (μ) and precision (τ). In turn, the prior for μ was a weakly informative normal distribution, and τ was equal to 1/$\sigma^{2}$, where the prior for σ was an uninformative half‐Cauchy distribution. For assessments of uninformative or weakly informative priors on the logit scale, particularly for occupancy models, refer to Gelman et al. (2008) and Northrup and Gerber (2018) or Broms et al. (2016). Our full set of priors are described in the supplement. In addition to the MSOM, we also fit SSOMs and a hybrid model. The hybrid model pooled data for $\alpha_{0k}$ and $\beta_{0k}$ (random effects) but not $\beta_{1k}$ (fixed effects) by excluding the community‐level hyperprior for the beta coefficient corresponding to treatment. We considered the hybrid model formulation based on the rationale that when $\beta_{1k}$ variance is large, species‐specific estimates might improve the most when intercepts are informed by community‐level effects, but treatment effects are modeled independently for each species. We fit all models in R version 4.5.0 (R Core Team 2025) using JAGS (Plummer 2003) and the jagsUI package (Kellner and Meredith 2024). Data simulation and model fitting were performed using U.S. Geological Survey Advanced Research Computing resources (Falgout et al., n.d.). Code to perform our full analysis is available in the accompanying software release (Cotterill and Graves 2025).

### 2.2 Data Simulation

**FIGURE 2:**
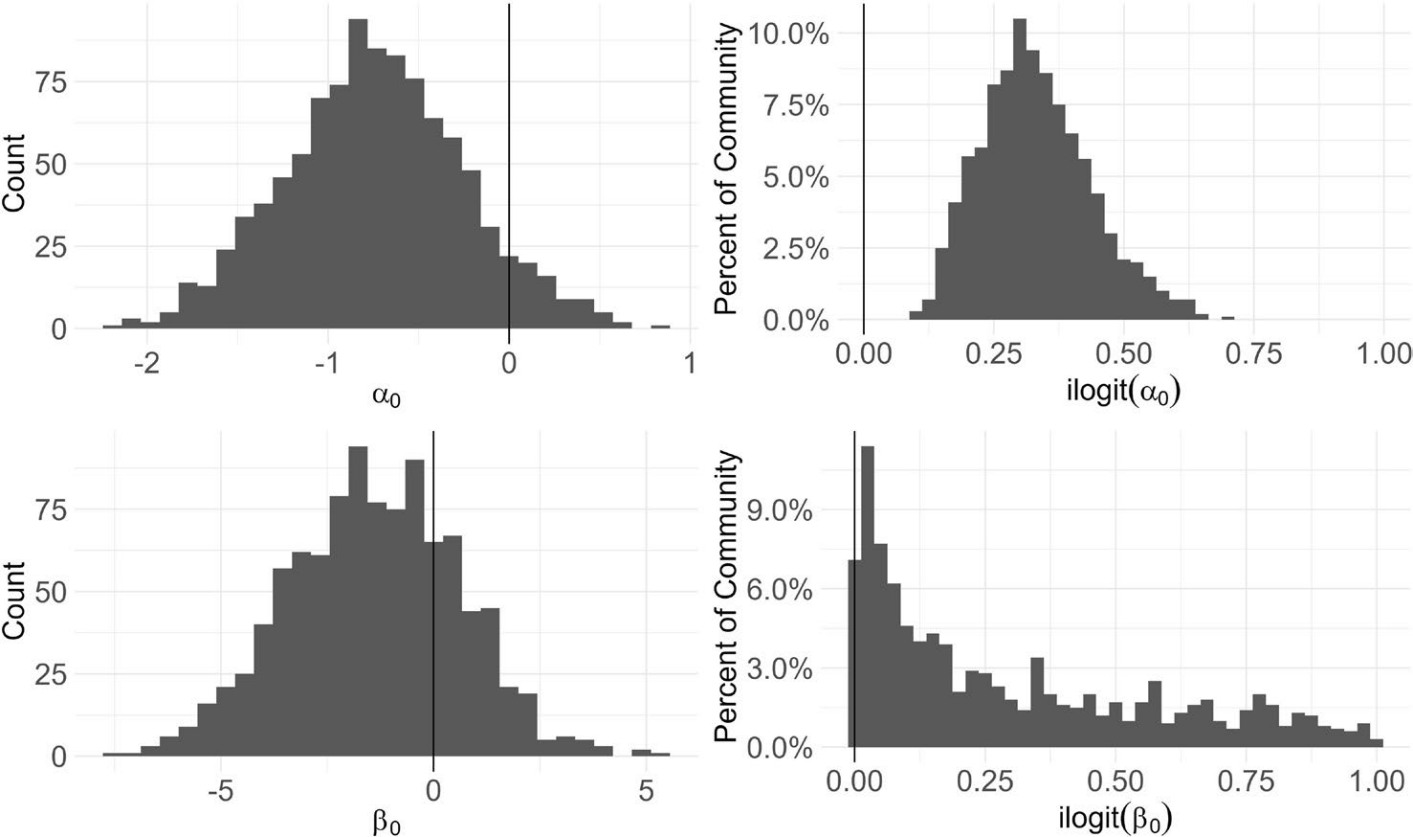
Histograms for 1000 draws of occupancy intercepts (top row) and detection intercepts (bottom row). Left, intercept values on the logit scale ($\beta_{0k}$ ~ Normal (−1.5, 2), $\alpha_{0k}$ ~ Normal (−0.75, 0.5)). Right, species‐specific intercept values on the probability scale represented as percentages of species within the community.

We simulated communities that included 25 or 50 species (or taxonomic units). This level of diversity reflects some real‐world pollinator communities (e.g., Kearns and Oliveras 2009), as well as resource bottlenecks (Woodard et al. 2020) that could constrain the size of communities monitored, while making the study more computationally tractable. Reported occupancy probabilities for pollinators tend to range more widely than detection probabilities (Bergman et al. 2004; Janousek et al. 2023; MacIvor and Packer 2016; McNeil et al. 2019; Nunes et al. 2024; van Strien et al. 2011). Therefore, species‐specific occupancy intercepts ($\beta_{0k}$) were drawn from a normal distribution with a mean of −1.5 and standard deviation of 2 (Figure 2). On the probability scale, this corresponds roughly to a negative exponential distribution comprised of many rare species and relatively fewer common species, which is a general pattern observed in community composition studies (Verberk 2011). Species‐specific detection intercepts ($\alpha_{0k}$) were drawn from a more constrained normal distribution with a mean of −0.75 and standard deviation of 0.5 which produced approximately normally distributed detection probabilities with a mean near 0.3 (Figure 2).

We evaluated two components of sample design—the numbers of sites and surveys—where surveys could represent sampling occasions (e.g., using human observers) or the number of samples collected (e.g., using eDNA samples) depending on the data collection method. We simulated 6, 8, 10, 20, 30, or 40 spatial replicates divided equally into treatment and control sites (e.g., 3 and 3 for 6 sites). The number of survey events was 2, 3, 4, 6, or 8, and all sites were surveyed the same number of times within each simulation.

**FIGURE 3:**
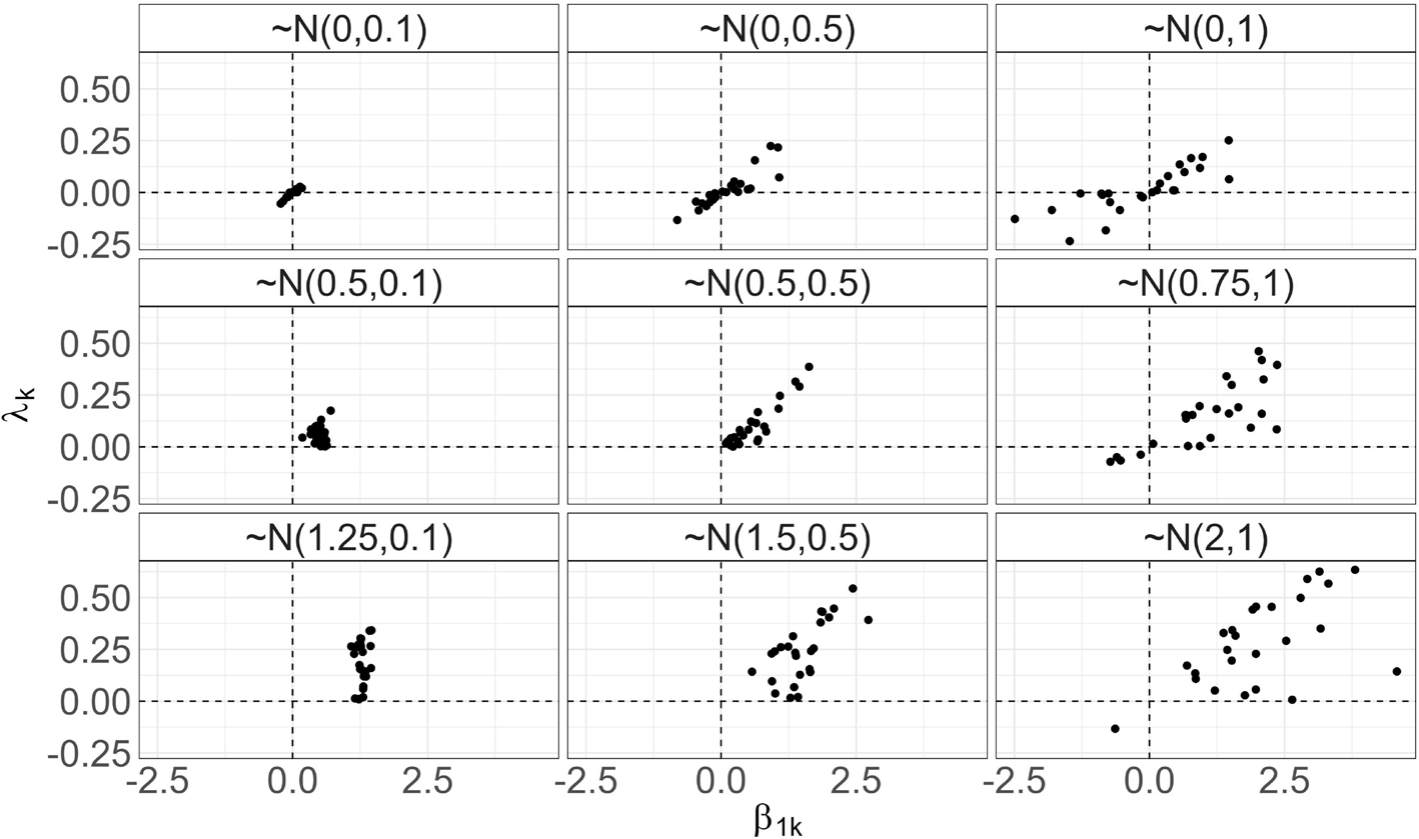
One realization of the nine treatment response scenarios for a community of 25 species. Each point represents one species. The derived quantity of interest, species‐specific treatment responses on the probability scale ($\lambda_{k}$, y‐axis), varies as a function of $\beta_{1k}$ (logit‐scale treatment effect) and each species' occupancy intercept ($\beta_{0k}$, not shown). $\beta_{1k}$ distributions (panel labels) reflect negligible, intermediate and strong mean effects (top to bottom) and small, intermediate and large variance (left to right). Small effects and large variance simulations yield treatment responses including both negative and positive effects.

Under each combination of community size, number of sites and number of surveys, we simulated nine treatment response scenarios. In each scenario, beta coefficient values corresponding to treatment effect ($\beta_{1k}$) were drawn from a normal distribution with varying means and standard deviations (Figure 3). The nine distributions were selected to encompass possible real‐world situations and illustrate how model performance changes as the magnitude of the simulated treatment effect ranges from negligible to large, and as within‐community variance increases.

These conditions generated 540 unique community and design combinations that were each simulated 100 times yielding 54,000 datasets. We fit the three model types (MSOMs, hybrid MSOMs and SSOMs) for all species that were observed at least once in simulated datasets and calculated several error metrics (refer to Error calculations section) to assess model performance. All models were run on three chains, with 20,000 iterations after burn‐in (*n* = 4000) thinned to every tenth sample (i.e., a total of 4800 samples kept; 16,000 * 3/10). We stored effective sample sizes and Gelman–Rubin statistics ($\hat{R}$) (Gelman and Rubin 1992) for the derived quantity of interest ($\hat{\lambda_{k}}$, the species‐specific probability scale treatment effects) and assessed convergence by ensuring that all $\hat{R}$ values ≤ 1.1.

### 2.3 Error Calculations

We calculated several error metrics for the species‐specific estimates of treatment effect ($\hat{\lambda_{k}}$). These included the mean (for all species within a simulated community) root‐mean squared error (RMSE), coverage and confidence interval widths, type 1 and type 2 error risk, bias of the community‐level treatment effect of the MSOM and what we refer to as “percent correctly classified” (PCC), a simple metric representing the degree of confidence and correct classification of the *direction* of a treatment effect (i.e., whether the estimate accurately categorized the treatment as helping or harming a species). For each set of 100 replicates, we calculated RMSE as follows:

$$\text{RMSE}=\sqrt{\frac{\sum {\left(\hat{\lambda_{k}}-\lambda_{k}\right)}^{2}}{100*\text{nspp}}}$$

where *nspp* was the number of species observed at least once in the community dataset. Coverage was the proportion of times that $\lambda_{k}$ was contained in the 95% credible interval for $\hat{\lambda_{k}}$, and credible interval widths were the difference between the 0.975 and 0.025 percentiles.

For simulations where $-0.01\leq \lambda_{k}\leq 0.01$ (negligible change in occupancy resulting from treatment), type 1 error risk was the proportion of times where the credible intervals of $\hat{\lambda_{k}}$ did not overlap 0 (false positive). For all other simulations (when there was a change in occupancy resulting from treatment), type 2 error risk was the proportion of times that the credible intervals of $\hat{\lambda_{k}}$ overlapped zero (false negative).

Managers may not always have the resources to collect sufficiently large datasets that yield precise and accurate species‐specific estimates of a treatment effect. They may nevertheless derive value from stating how confident they are that there is *some* effect, and the direction of that effect (helping or harming species). We calculated the PCC of the direction of a treatment effect as the proportion of the posterior distributions for $\hat{\lambda_{k}}$ that were correctly classified as positive or negative.

Species of conservation concern might not always be the rarest species at sampling locations. However, we suspected that rare species—those with the fewest observations—would be most sensitive to shrinkage effects in the MSOM and sought to highlight these examples in comparing models' performance. Therefore, to evaluate sample designs needed for species of conservation concern within a larger community, we recalculated these error metrics on a subset of rare species. We used the rarest 10% of species in our simulated communities based on the distribution of occupancy intercepts ($\beta_{0k}\sim N\left(-1.5,2\right)$). The occupancy threshold corresponding to these ‘rare species’ was calculated on the logit scale by taking the inverse cumulative density of the occupancy intercept distribution at a 10% probability ($\beta_{0}=-4.06$). This corresponds to a 1.7% occupancy probability at control sites.

## 3 Results

### 3.1 Detection of Species in Simulated Data

**FIGURE 4:**
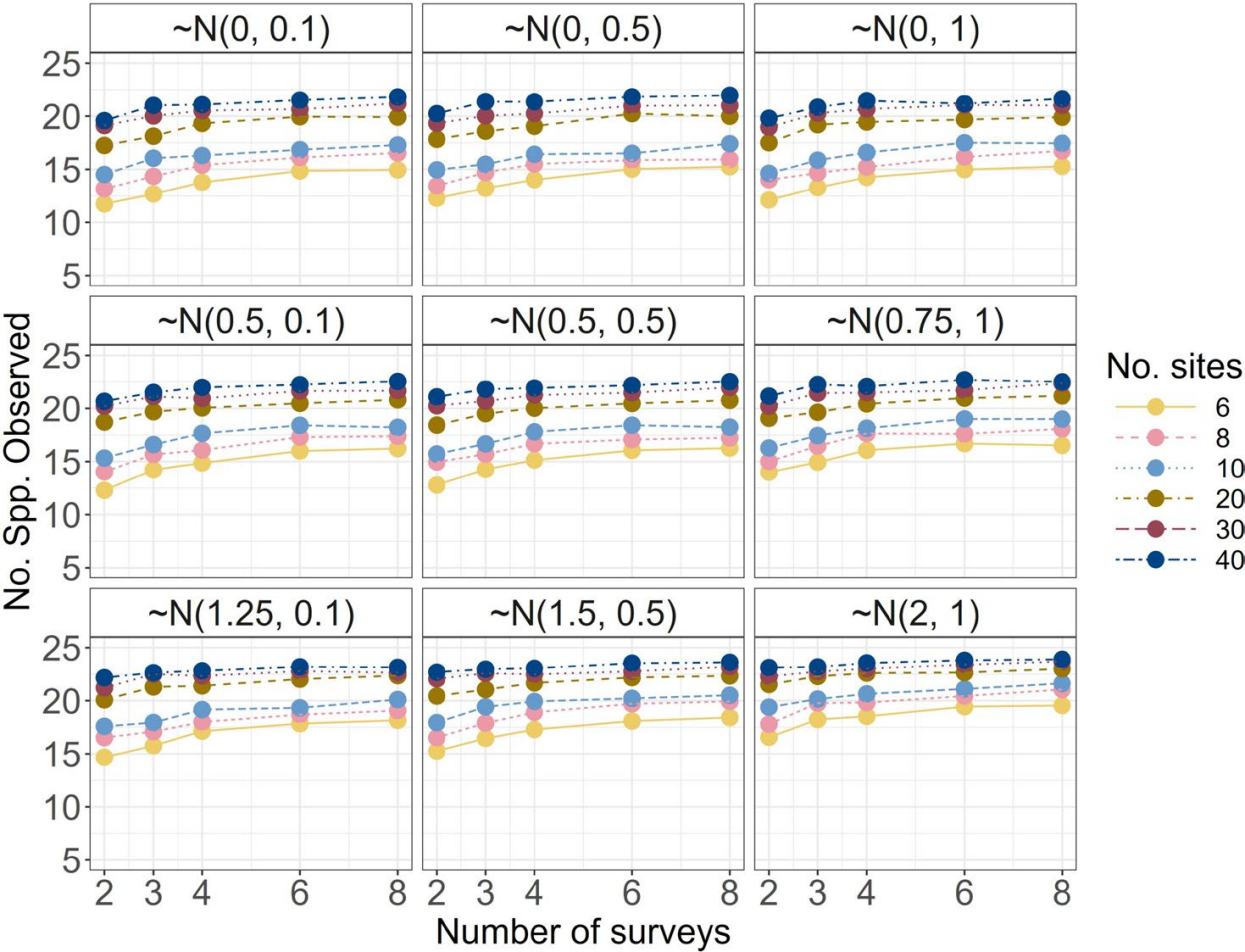
Average number of species observed across 100 simulated datasets at a given set of design parameters for a community of 25 species. Panels are the response scenarios ($\beta_{1k}$ distributions labeled).

**FIGURE 5:**
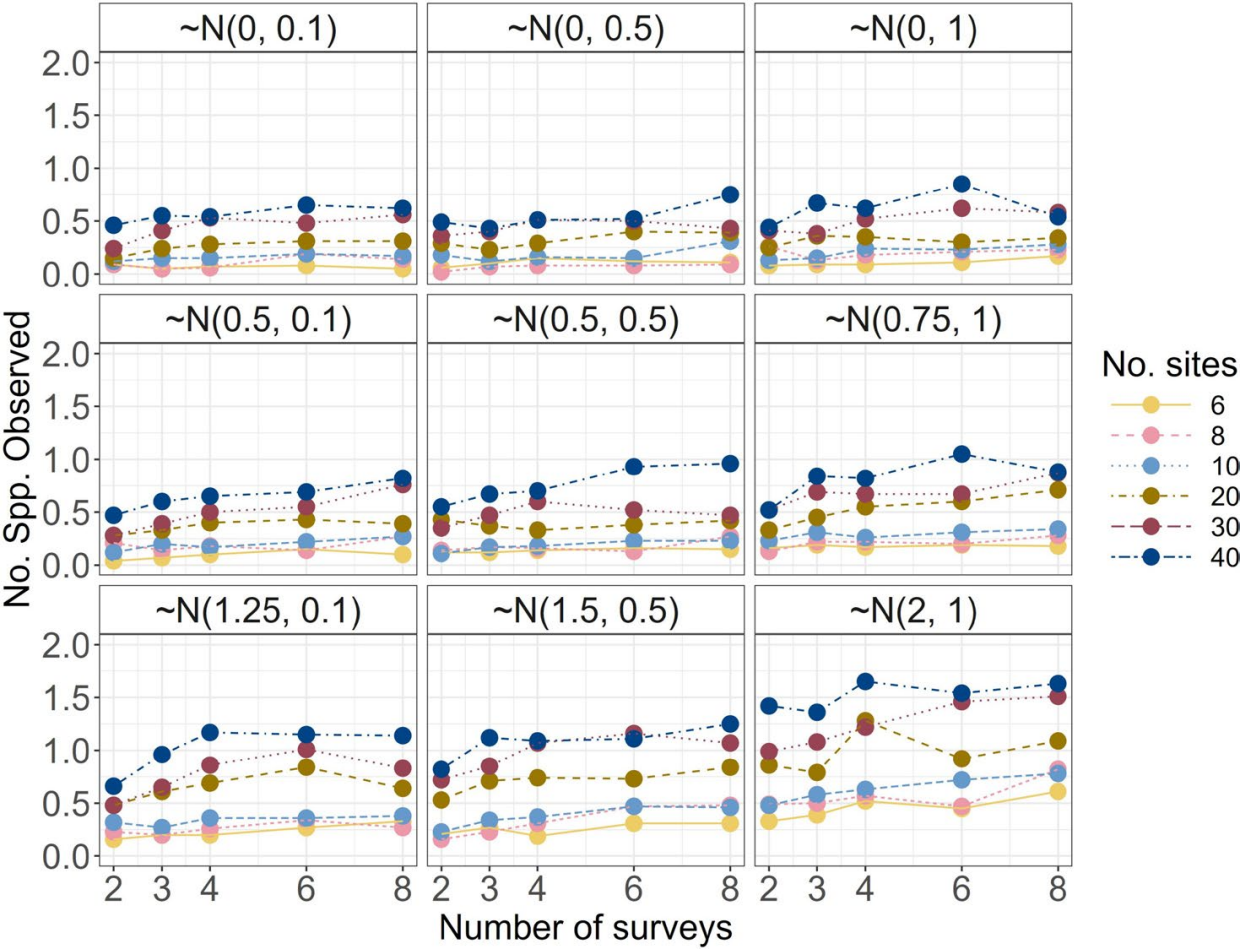
Average number of rare species (10th percentile lowest occupancy) observed across 100 simulated datasets at a given set of design parameters for a community of 25 species. Panels are the response scenarios ($\beta_{1k}$ distributions labeled).

As expected, the total number of species observed in simulations increased with higher sampling intensity and varied based on the chosen occupancy and detection intercepts (Figures 4 and S1a). On the basis of the distributions of our model parameters, the lowest sampling intensity (6 spatial replicates, 2 surveys) detected approximately half of the species in a community when there was a negligible habitat treatment effect. The highest level of sampling intensity increased species detections by approximately 30%–40%, depending also on the treatment effect scenario (Figure 4). A “rare species” (in the 10th percentile least likely to occupy sites) was detected in fewer than 50% of simulations (across all sites) unless sampling intensity was high and treatment effect magnitude was large for a community of 25 species (Figures 5 and S1b).

### 3.2 Model Comparison

We found little difference in any of the error metrics between the two community sizes considered (25 or 50 species; details in supplement). Poor convergence ($\hat{R}$ values exceeding 1.1) for species‐specific treatment effect estimates was rare. $\hat{R}$ values > 1.1 did not occur for SSOMs, but occurred in non‐overlapping instances in both the MSOM and hybrid model. Roughly half of these convergence issues occurred when species were observed fewer than three times in a dataset. In total, the convergence issues amounted to less than 1% (0.29%) of all simulated species. We removed this small number of individual estimates from all error metric calculations for all three model types.

**FIGURE 6:**
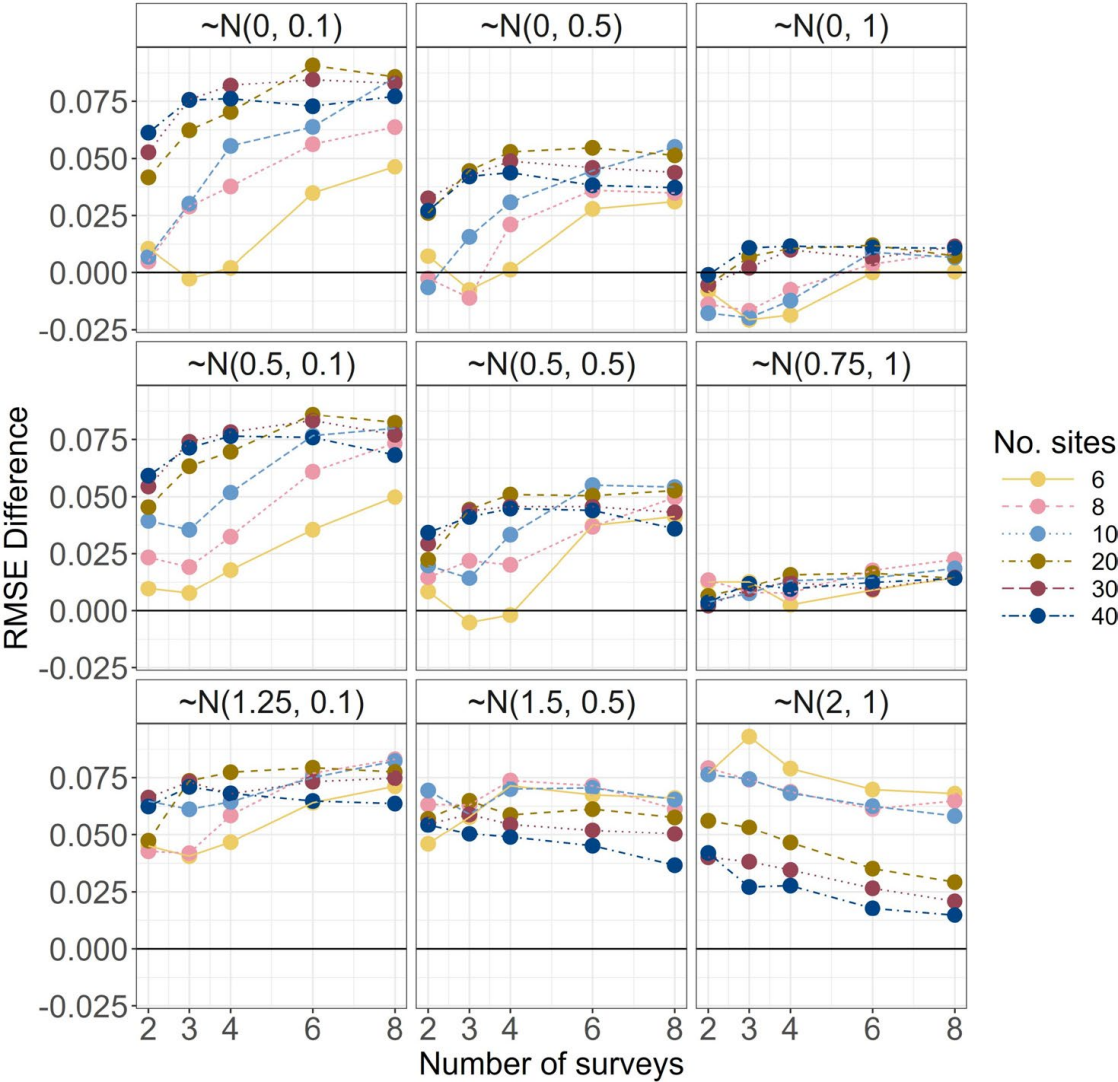
Differences in mean (across species) root‐mean squared error (RMSE; $\mathrm{RMS}E_{\text{diff}}=\mathrm{RMS}E_{\text{SSOM}}-\mathrm{RMS}E_{\text{MSOM}}$) across 100 simulations of each study design for nine treatment response scenarios (panels, $\beta_{1k}$ distributions labeled). Y‐axis values greater than zero indicate where the multispecies occupancy model outperformed single species occupancy models. Communities of 25 species shown.

**FIGURE 7:**
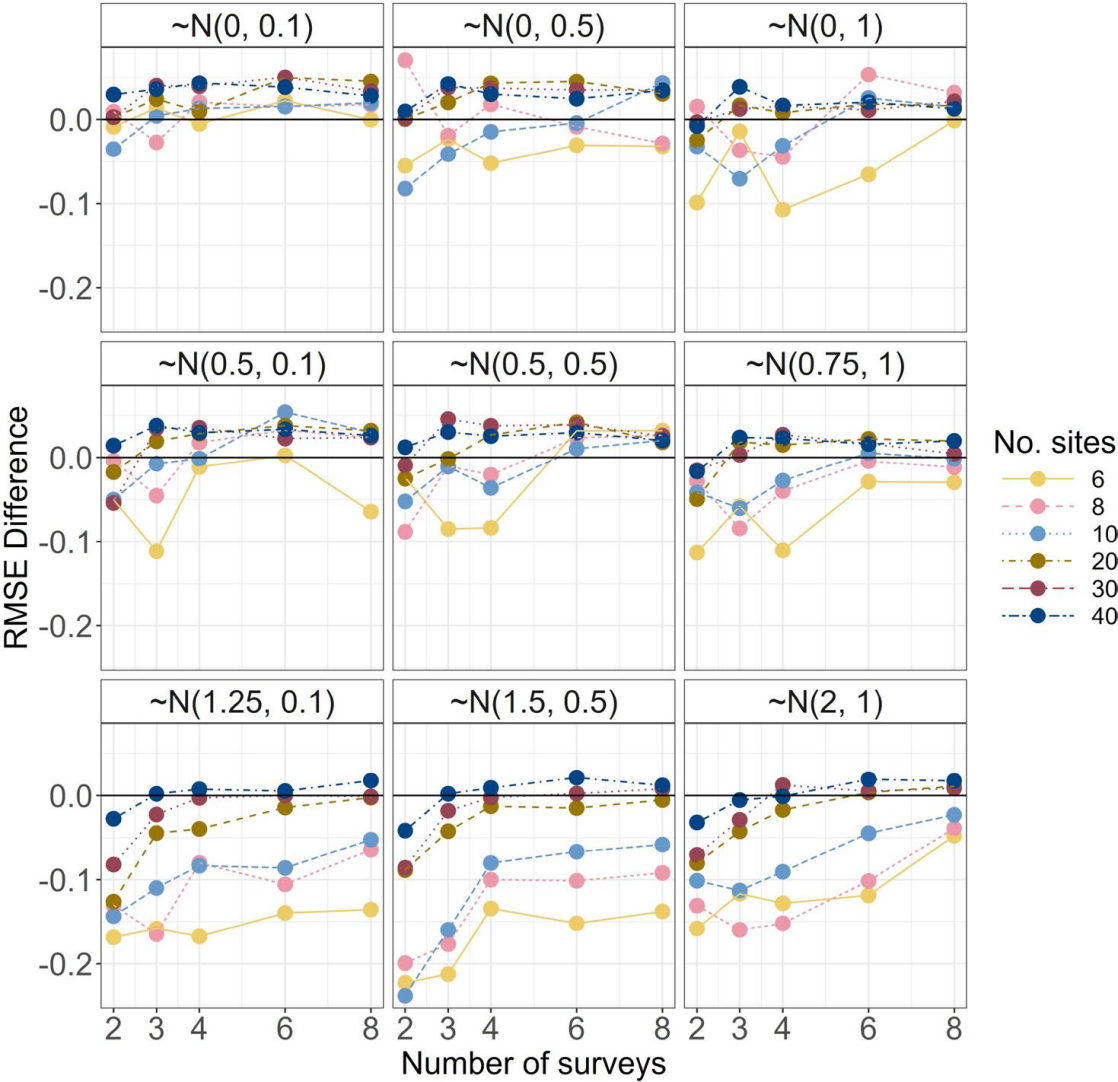
RMSE differences between SSOM and MSOM for “rare” species (10th percentile of the occupancy intercept distribution). Below zero indicates MSOM point estimates for treatment effect were less accurate than SSOM point estimates. Above about four surveys and 40 sites, MSOMs perform as well or better than SSOMs regardless of the treatment response scenario. The more similarly species respond to treatment (response scenarios with smaller standard deviations), the less sampling is required. However, for these rarely detected species, SSOMs generally produce more reliable estimates. Communities of 25 species shown.

The performance of the hybrid MSOM relative to the SSOM and a full MSOM was uncompelling. We therefore focus on summarizing results of the latter two models. For full hybrid model results, refer to the supplement. The degree of similarity to treatment response (positive vs. negative) influenced model performance as measured by mean root‐mean squared error (RMSE; refer to Figure S2a–f). RMSE was generally lower (indicating better model performance) using the multispecies occupancy model (MSOM) compared to modeling all species using single species occupancy models (SSOMs; Figure 6). However, increasing variation in species' treatment responses decreased MSOM model performance, as did smaller mean effect magnitudes (particularly those that were zero‐centered; Figure 6). Additionally, our simulations included some scenarios in which SSOMs outperformed MSOMs based on RMSE, particularly at lower levels of sampling intensity (Figure 6) and when considering rare species (Figure 7). For rare species, SSOMs had lower RMSEs except under both high levels of sampling intensity and low variance in response. In those scenarios, RMSE was marginally reduced using the MSOM.

**FIGURE 8:**
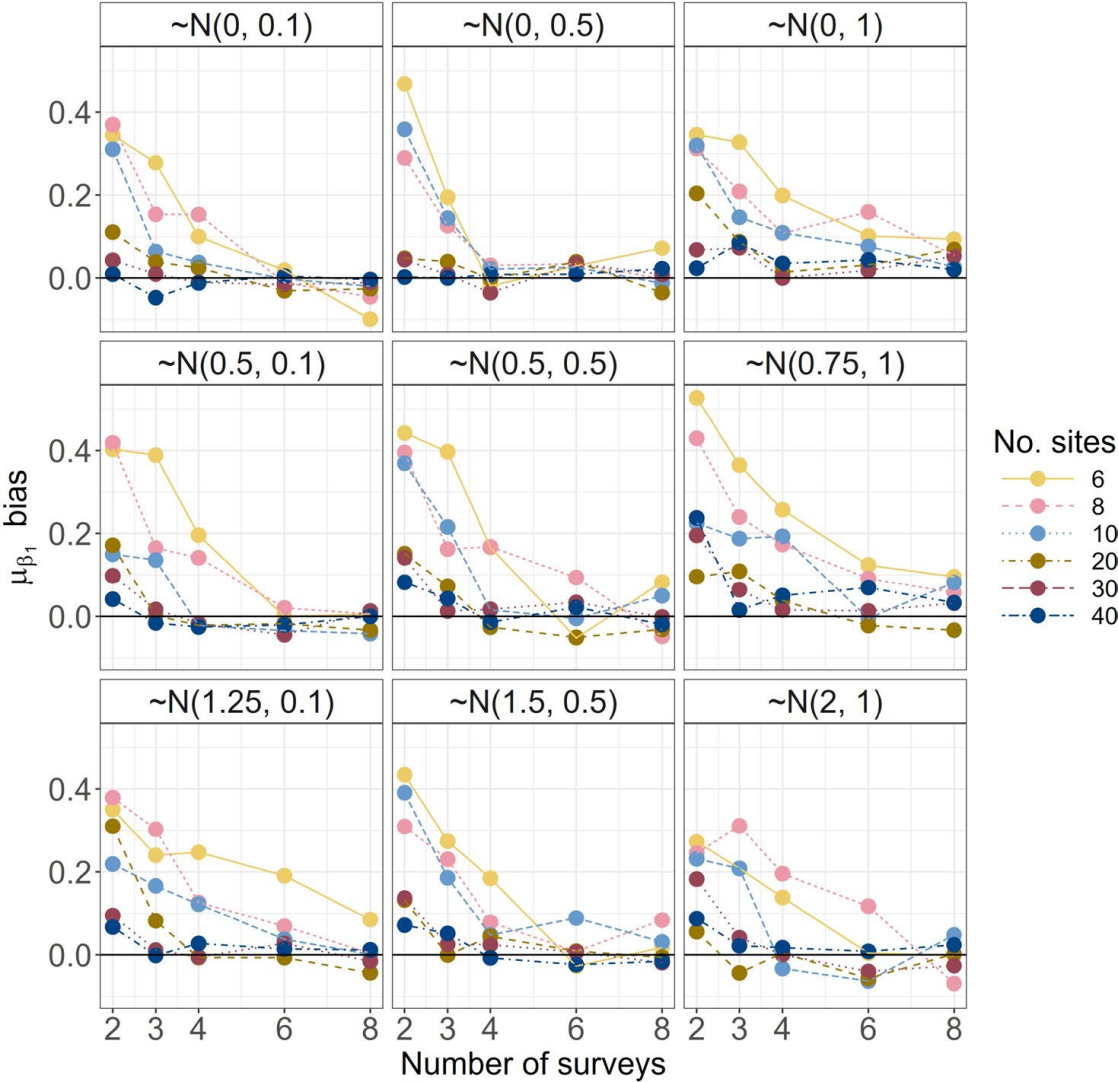
Bias in median community‐level estimates for the treatment effect beta coefficient ($\mu_{\beta_{1}}$) under the multispecies occupancy model (communities of 25 species shown). Model estimates were biased high below four surveys and 20 sites. Above this threshold estimates were relatively stable and unbiased regardless of the true distribution of $\beta_{1}$.

Calculated using the entire community, coverage (the proportion of times $\lambda_{k}$ was within the 95% confidence interval of $\hat{\lambda_{k}}$) was generally near 95% regardless of the model type or scenario (refer to Figure S3a–f). Exceptions occurred when limiting these calculations to rare species under the MSOM at low sampling intensities due, in part, to the relatively higher precision of $\hat{\lambda_{k}}$ estimates under the MSOM (refer to Figure S4a–f). In conjunction with the relatively lower precision of SSOMs compared to MSOMs, type 1 error risk was virtually non‐existent using SSOMs but high for MSOMs under large effect magnitude scenarios (refer to Figure S5a–f). In large effect magnitude scenarios, few species exhibit no (or negligible) treatment effect. Similarly, type 2 error risk was highest under the SSOM and lowest under the MSOM, especially for large effect magnitude scenarios (refer to Figure S6a–f). The community‐level treatment response estimates of the MSOM ($\mu_{\beta_{1}}$; the only model type including this hyperparameter) were biased high, regardless of the response scenario, when sampling intensity was less than 20 sites and four surveys (Figure 8).

**FIGURE 9:**
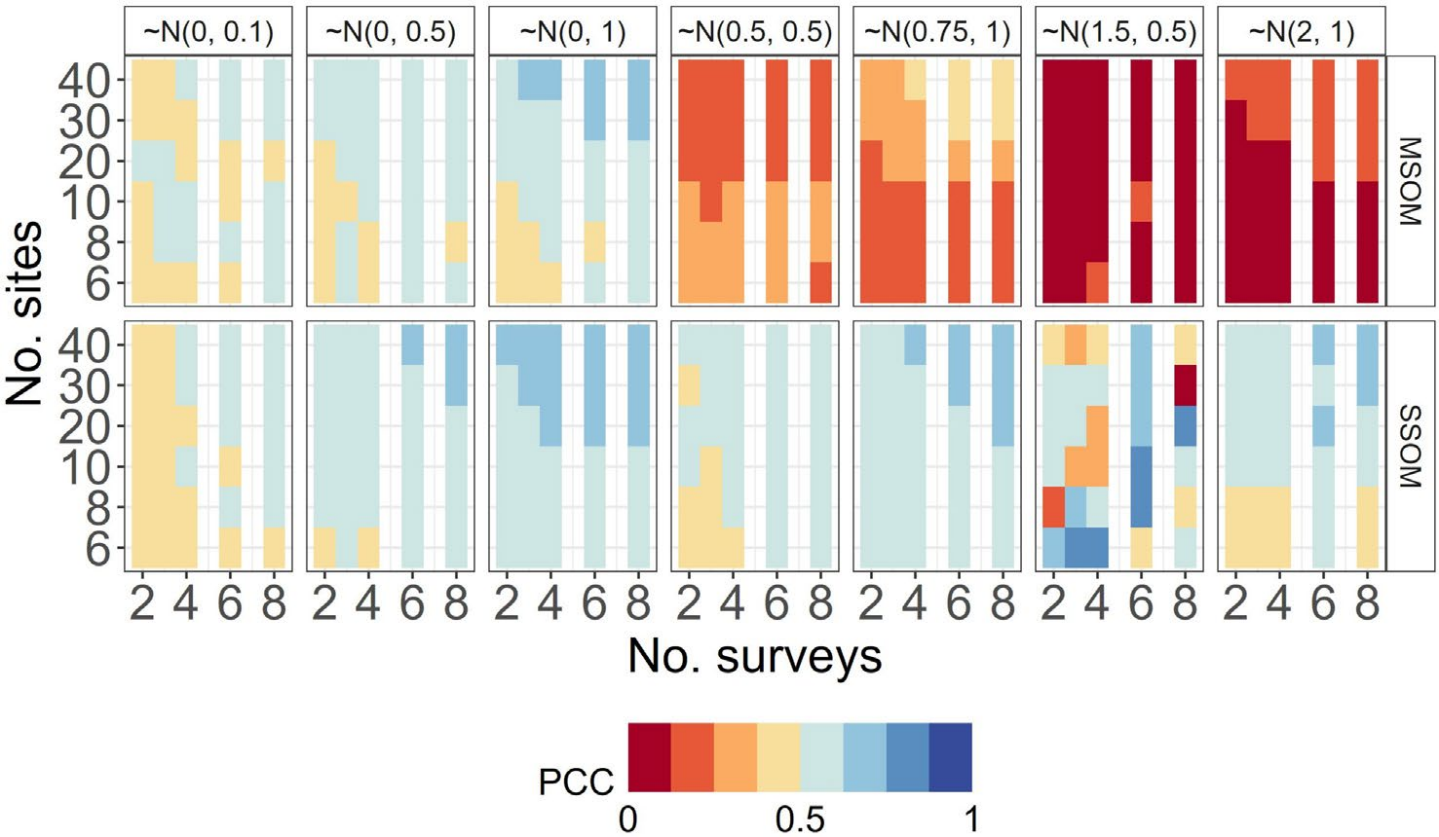
Mean percent of posterior distributions that were correctly classified as harming species (PCC) based on species‐specific estimates of treatment effects (for species where $\lambda_{k}$ < −0.01). As the mean effect magnitude increases (left to right), fewer species are negatively affected and not all scenarios are represented. However, the MSOM performs poorly (wrong 87.5–100% of the time under some scenarios), in part, because the hyperparameter is biased high at lower levels of sampling. At higher levels of sampling, the community‐level effect swamps the individual‐level effects, errantly estimating most of the posterior distribution > 0. SSOMs, although less precise and still imperfect, are more robust to these outlier species especially when there is wide variability in species‐level responses (e.g., SD = 1).

The “percent correctly classified” (PCC) provided an alternative method of quantifying model accuracy based on a binary characterization of treatment (positive or negative effect on occupancy) using the full posterior distributions of species‐specific estimates (refer to Figure S7a–f). However, PCC values for MSOMs could be misleading, especially for large effect magnitude scenarios, because of hyperparameter bias in the direction of the simulated effects. On the basis of the findings of hyperparameter bias, and using the precautionary principle as our guide, we calculated the mean percent of posterior distributions that were correctly classified as *harming* species (i.e., where $\lambda_{k}$ < −0.01). Harmed species occurred in simulation least frequently in scenarios with larger mean effect magnitudes but smaller within‐community variation. The MSOM performed poorly (wrong 87.5%–100% of the time under some conditions) in these rare circumstances (Figure 9).

**FIGURE 10:**
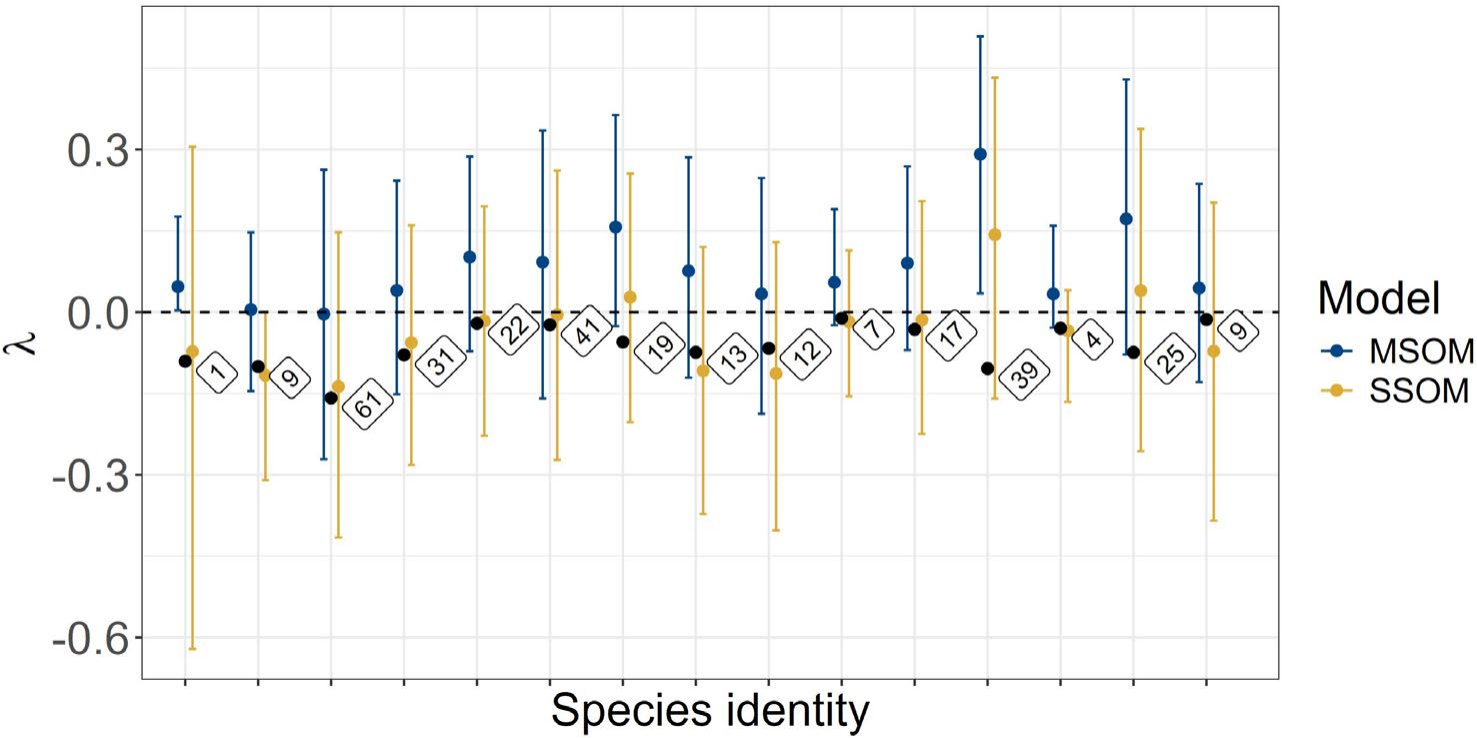
True simulated treatment effects (black dots) compared against MSOM and SSOM estimates for treatment effect (median and 95% credible intervals shown) for fifteen randomly sampled species that were harmed by treatment in the scenario where mean effect magnitude and variance were highest. Numeric labels show the number of times each species was observed in the simulated data.

To further contextualize the inferential differences between the models, we selected one simulated design with 50 species in the community, sampled at 40 sites, with eight surveys, and a strong but variable treatment effect ($\beta_{1k}∼N\left(2,1\right)$). Across 100 simulated replicates of this design, 95% of simulated species were detected (4742 of 5000). In this scenario, 1.5% (72/4742) of detected species were adversely affected by treatment ($\lambda_{k}\leq -0.01$). We then randomly sampled a subset of these species (Figure 10). For these outlier species, inference improved under SSOMs compared to the MSOM: point estimates were more often correctly categorized as negative, and uncertainty was appropriately higher when species were detected very few times. MSOM estimates always produced higher precision, even when a species was observed only once in the data.

## 4 Discussion

Our analysis indicated that when species‐level, rather than community‐level, processes are the main question of interest to researchers, single‐species occupancy models (SSOMs) outperform multispecies occupancy models (MSOMs) in some circumstances. The risk of incorrect species‐level inference under the MSOM increases with low sampling intensity and high within‐community effect variance. Addressing this risk will depend on the goals and scope of a study. Modelers can determine when to conduct SSOM analyses in addition to MSOMs by weighting their confidence in species‐specific effect estimates based on the number of observations of a species and our results: simply relying on posterior credible intervals can be insufficient (Figure 10). For community‐level monitoring across the range of our simulated scenarios, MSOMs generally produced greater accuracy and precision for evaluating habitat management actions on species. However, treatment effect estimates of MSOMs were biased below approximately 20 spatial replicates with four repeat surveys. This suggests a minimum sampling intensity threshold within the design considerations of our simulations, and with respect to the parameter distributions we selected for occupancy and detection intercepts. When focal species are rare or difficult to detect, it is noteworthy, albeit not unexpected, that they may never be observed at lower levels of sampling.

Under the MSOM, identifying which species should be modeled as a “community” ideally follows species' responses to covariates—in the context of our study, whether species respond positively (increased occupancy) or negatively (decreased occupancy) to habitat treatment. Yet, treatment response is often what practitioners seek to study, rather than information available a priori. For many taxa, including pollinators and rare species, published information to justify a priori groupings for treatment responses or other environmental covariates may be lacking. In statistical terminology, our simulated species responses technically met the exchangeability assumption in that their parameter values were drawn from shared distributions (Bernardo 1996; Gelman et al. 2013). In practical terms, the number of species modeled as a community comprises a relatively small sample from these distributions, which impedes MSOM performance as variation increases.

Scenarios that violate the exchangeability assumption entirely (that we did not simulate) may occur if distinct functional groups exist within the surveyed community. For example, butterflies (order *Lepidoptera*) and bumblebees (*Bombus* spp.) are pollinators, but modeling them together as a single community, rather than as distinct communities, may be counterproductive. Not only are there documented differences in their responses to some management actions like alternative grazing practices and prescribed burning (Leone et al. 2022), but their detection probability distributions may also wildly differ depending on the survey method and design (Boone et al. 2023; Kral‐O'Brien et al. 2020; McNeil et al. 2019; Patterson et al. 2023). In this example, one may expect a community definition encompassing both taxonomic groups to feature detection intercept and treatment response distributions that are strongly bimodal. As we demonstrate, even less severe violations of this principle have consequences within the scope of the sampling intensities we explored. As species' responses diverge and become more variable, MSOMs risk pulling species in the tails of response distributions too far in the direction of an estimated community mean effect, which can change the direction of the estimated effect for those species. Practitioners operating under severe sampling constraints may mitigate these risks by fitting both SSOMs and MSOMs to their data and evaluating whether species‐specific coefficients (or derived quantities) are so different as to influence their conclusions. We considered a hybrid MSOM with no treatment effect hyperprior as a potential intermediate solution to this problem, but in general its performance relative to the SSOMs and a full MSOM was uncompelling (refer to supplement). Although additional modeling may sound like much more work, moving from an MSOM to SSOM framework is straightforward: removal of the MSOM hyperpriors fits all SSOMs simultaneously.

Ideally, study design begins with a power analysis tailored to its goals (Devarajan et al. 2020). Yet, this becomes increasingly burdensome as the state‐space under consideration expands. Our state‐space comprised relatively small communities, relatively low sampling intensity and only three species‐level parameters. Even so, performing 100 replicates of each design and scenario combination took about 16 h per model type when distributed across 128 cores on a high‐performance computer. Each model run would have taken up to 2 weeks to run on a desktop computer with access to 16–32 cores. More efficient Bayesian samplers, such as those within NIMBLE (de Valpine et al. 2017), or Stan (Carpenter et al. 2017), could reduce computational time, but also require additional expertise.

Despite the ubiquity of habitat restoration and conservation actions, rigorous assessment of outcomes is lacking (Binley et al. 2025; Sutherland et al. 2004) and public land managers operate within severe resource constraint limitations (Smiley 2008). Among their various duties, they are tasked with monitoring rare and threatened species, evaluating the effects of experimental habitat treatments on species of concern and making adaptive management decisions (Moore et al. 2011). As such, identifying the minimum amount of data needed to estimate meaningful species‐specific effects supports management decisions about how to collect data for assessment of treatment effects. Our results and published code can help research support staff in these efforts (Cotterill and Graves 2025). Survey design tradeoffs often call for case‐by‐case consideration and MSOMs improve the precision of parameter estimates, which could reduce sampling requirements to achieve a certain level of confidence when researchers seek to understand mean effects on communities. Yet, our results show that when treatment effects on individual species are of primary importance, particularly with large and variable treatment effects and lower sampling efforts, additional modeling for those species may be needed.

Although we focused on pollinators in the western United States to inform regional goals, the conditions we selected can inform sample design and analysis across species groups (e.g., birds, bats, insects, or fish communities) and data collection methods (e.g., eDNA, netting, or timed observations). These results provide a benchmark for study design in systems where the goal is to efficiently evaluate treatment effects on species of conservation concern. The relative costs associated with specific projects can vary depending on sampling method, the accessibility of sites, or the availability of trained personnel. When approximate costs are known, these can be formally incorporated to develop an informative study design using optimization procedures (for an example, refer to Sanderlin et al. 2014). The results here could also be used to identify sampling options of varying site and survey numbers and assess the trade‐offs of those designs for the best logistics and costs for a particular manager.

## Author Contributions

**Gavin G. Cotterill:** conceptualization (equal), formal analysis (lead), methodology (lead), software (lead), visualization (lead), writing – original draft (lead), writing – review and editing (equal). **Douglas A. Keinath:** conceptualization (equal), formal analysis (supporting), funding acquisition (equal), project administration (supporting), supervision (supporting), writing – original draft (supporting), writing – review and editing (equal). **Tabitha A. Graves:** conceptualization (equal), formal analysis (supporting), funding acquisition (equal), methodology (supporting), project administration (lead), supervision (lead), visualization (supporting), writing – original draft (supporting), writing – review and editing (equal).

## Conflicts of Interest

The authors declare no conflicts of interest.

## Supporting information


**Appendix S1:** ece372315‐sup‐0001‐AppendixS1.docx.

## Data Availability

The code used in this study is available as a U.S. Geological Survey software release. Researchers interested in reproducing or building upon the findings can access the code through this link: https://doi.org/10.5066/P13JU6YW.
