# Supplementary material for: When Do Single‐Species Occupancy Models Outperform Multispecies Models?
Source: Ecol Evol. 2025 Nov 23;15(11):e72315. doi: 10.1002/ece3.72315 (PMC12640701; doi:10.1002/ece3.72315)
Supplement: Supplementary file 1 — Appendix S1: ece372315‐sup‐0001‐AppendixS1.docx. [file ECE3-15-e72315-s001.docx]

Supplement to: When do single species occupancy models outperform multispecies models?

2025-08-12

## Authors

Gavin G. Cotterill^1^, Douglas A. Keinath^2^, and Tabitha A. Graves^1^

^1^ U.S. Geological Survey, Northern Rocky Mountain Science Center, 38 Mather Drive, PO Box 169, West Glacier, MT 59936, USA

^2^ U.S. Fish and Wildlife Service, Wyoming Ecological Services Field Office, 334 Parsley Blvd, Cheyenne, WY 82007, USA

Corresponding Author: Gavin G. Cotterill, [gcotterill@usgs.gov](mailto:gcotterill@usgs.gov)

# Prior specification

## Single species occupancy models (SSOMs)

The species-specific priors were as follows:

$$\begin{matrix} \beta_{0k}\sim dnorm\left( 0,{2.25}^{-2} \right) \\ \beta_{1k}\sim dnorm\left( 0,{2.25}^{-2} \right) \\ \alpha_{0k}\sim dnorm\left( 0,{2.25}^{-2} \right) \end{matrix}$$

## Multispecies occupancy models (MSOMs)

The community level hyperpriors were as follows:

$$\begin{matrix} \mu_{\beta0}\sim dnorm\left( 0,{2.25}^{-2} \right) \\ \sigma_{\beta0}\sim halfCauchy\left( 2 \right) \\ \tau_{\beta0}=\frac{1}{\sigma_{\beta0}^{2}} \\ \mu_{\beta1}\sim dnorm\left( 0,{2.25}^{-2} \right) \\ \sigma_{\beta1}\sim halfCauchy\left( 2 \right) \\ \tau_{\beta1}=\frac{1}{\sigma_{\beta1}^{2}} \\ \mu_{\alpha0}\sim dnorm\left( 0,{2.25}^{-2} \right) \\ \sigma_{\alpha0}\sim halfCauchy\left( 2 \right) \\ \tau_{\alpha0}=\frac{1}{\sigma_{\alpha0}^{2}} \end{matrix}$$

The species-specific priors were as follows:

$$\begin{matrix} \beta_{0k}\sim dnorm\left( \mu_{\beta0},\tau_{\beta0} \right) \\ \beta_{1k}\sim dnorm\left( \mu_{\beta1},\tau_{\beta1} \right) \\ \alpha_{0k}\sim dnorm\left( \mu_{\alpha0},\tau_{\alpha0} \right) \end{matrix}$$

## Hybrid model

The community-level hyperpriors were as follows:

$$\begin{matrix} \mu_{\beta0}\sim dnorm\left( 0,{2.25}^{-2} \right) \\ \sigma_{\beta0}\sim halfCauchy\left( 2 \right) \\ \tau_{\beta0}=\frac{1}{\sigma_{\beta0}^{2}} \\ \mu_{\alpha0}\sim dnorm\left( 0,{2.25}^{-2} \right) \\ \sigma_{\alpha0}\sim halfCauchy\left( 2 \right) \\ \tau_{\alpha0}=\frac{1}{\sigma_{\alpha0}^{2}} \\ \end{matrix}$$

The species-level priors were as follows:

$$\begin{matrix} \beta_{0k}\sim dnorm\left( \mu_{\beta0},\tau_{\beta0} \right) \\ \beta_{1k}\sim dnorm\left( 0,{2.25}^{-2} \right) \\ \alpha_{0k}\sim dnorm\left( \mu_{\alpha0},\tau_{\alpha0} \right) \end{matrix}$$

# Results

‘Rare’ species were defined as those whose true occupancy intercept values were in the 10th percentile of the occupancy intercept distributions.

## The average number of species observed per design

### All species


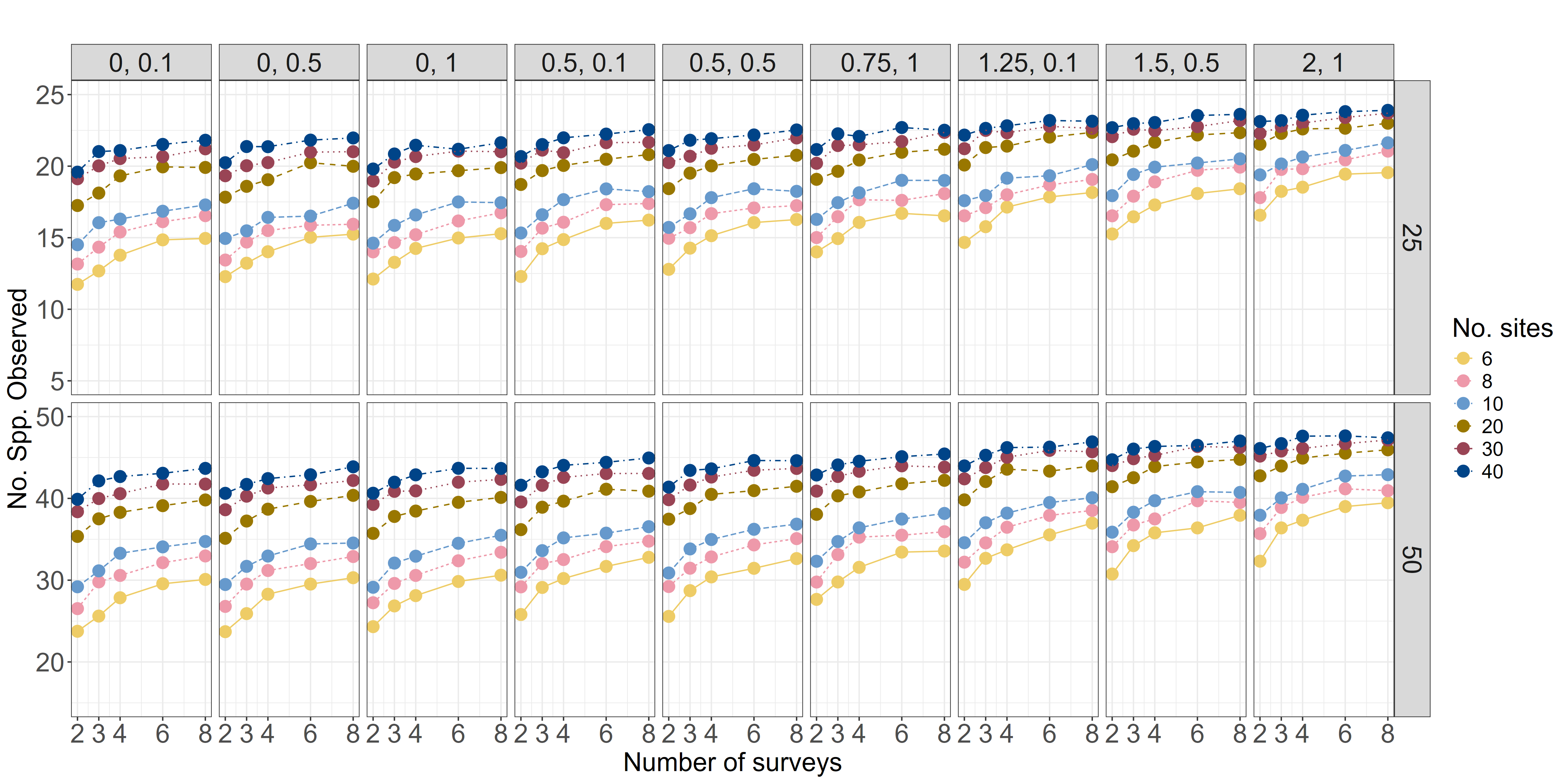


Figure 1a. More intensive sampling increases the total number of species detected. Columns vary by the normal distribution parameters governing the treatment effect scenario. Rows vary by the number of species in the community.

### Only rare species


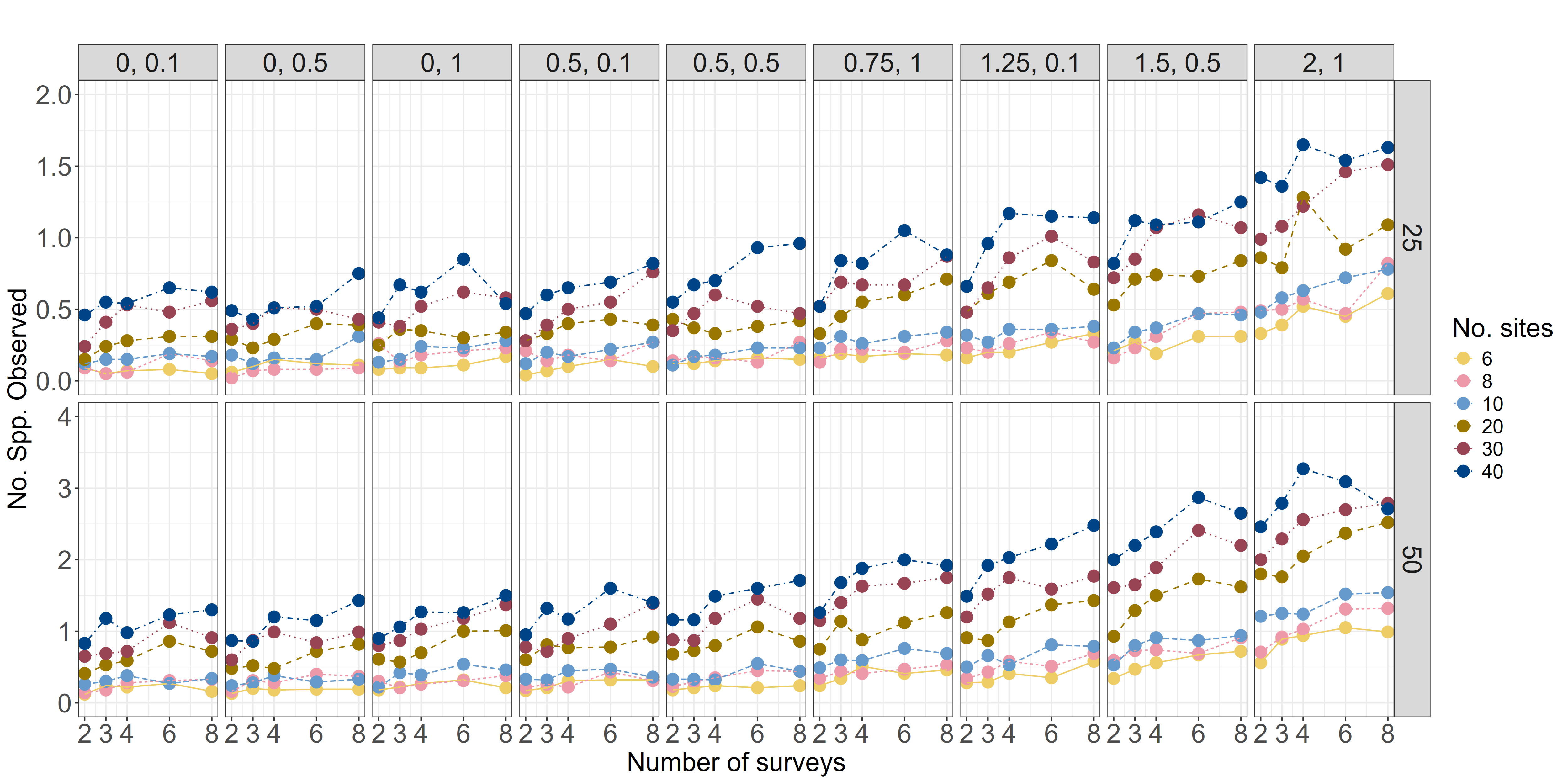


Figure 1b. An average of <1 ’rare’species (in the 10th percentile of occupancy intercept distribution) was detected per simulation. Larger communities and larger average treatment effects increased rare species detections. Columns vary by the normal distribution parameters governing the treatment effect scenario. Rows vary by the number of species in the community.

## Convergence

Across 54,000 simulations, SSOMs returned zero instances where $\hat{\lambda_{k}}$ values were $\hat{R}>1.1$. The MSOM returned 4,352 such values. The hybrid model returned 188 such values. Across the latter two models, none of the problematic simulated species were identical. Thus, we omitted a total of 4,540 simulated species when calculating error metrics. This amounted to 0.29% (less than one percent) of all simulated and observed species. Roughly half of these had three or fewer observations in simulated datasets. We calculated all error metrics with and without the species-specific values which failed to converge to ensure they did not impact inference. We present the results omitting these species.

## Mean root-mean squared error (RMSE)

RMSE varied by model type (SSOM/MSOM/hybrid), treatment effect size and variance, and study design (number of sites and surveys).

$$RMSE=\surd\frac{\sum\left( \hat{\lambda}_{k}-\lambda_{k} \right)^{2}}{100*nspp}$$

Where *nspp* was the number of species detected in a simulated dataset with $\hat{R}<=1.1$ and there were 100 replicates for each study combination.

### All species - SSOM


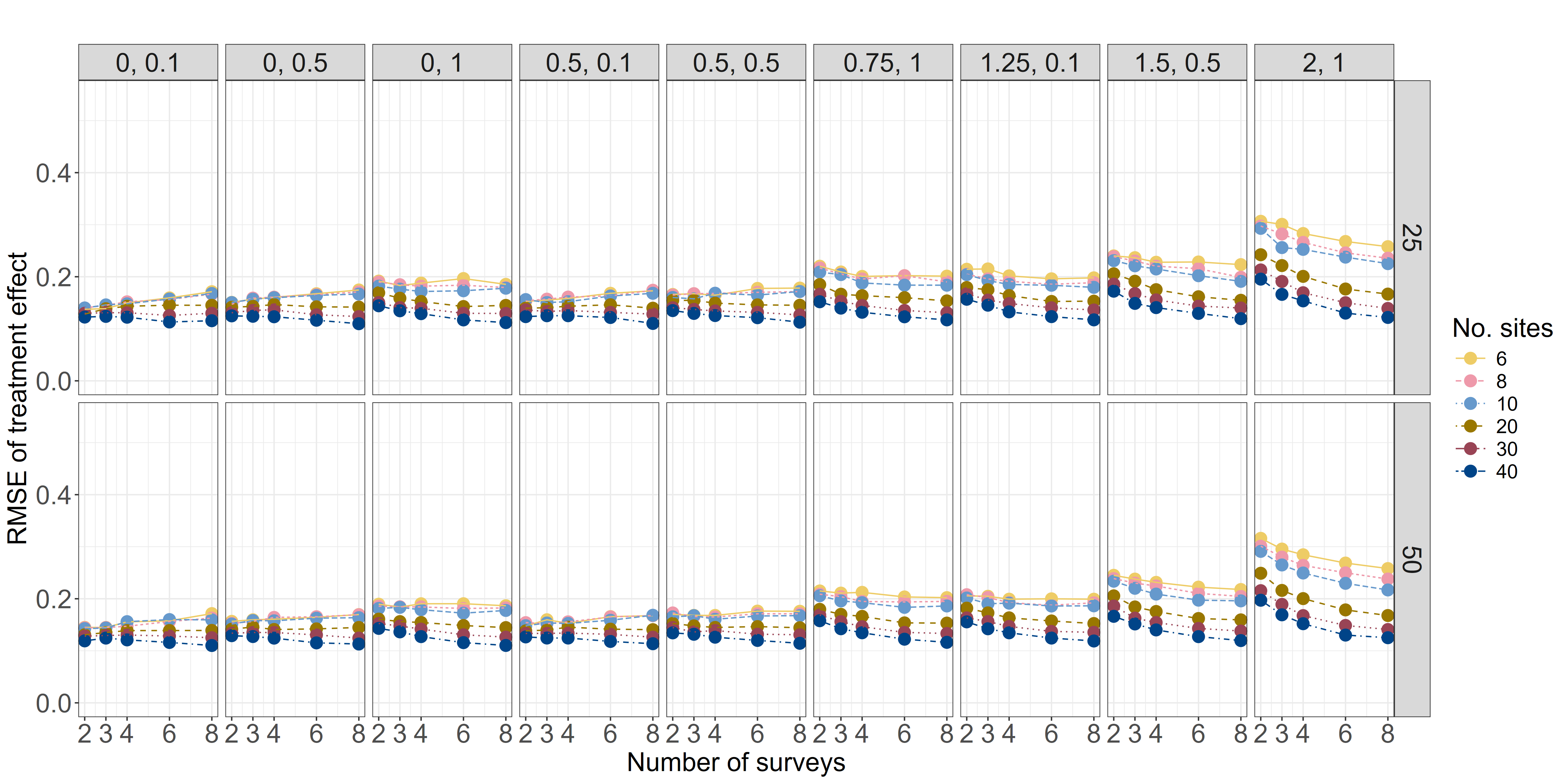


Figure 2a. Comparing root-mean squared error (RMSE) of estimated treatment effect for 100 replicates at each design X simulation combination when single-species occupancy models were fit for all species in the community. Columns vary by the normal distribution parameters governing the treatment effect scenario. Rows vary by the number of species in the community. RMSE increased with larger treatment effect variance.

### All species - MSOM


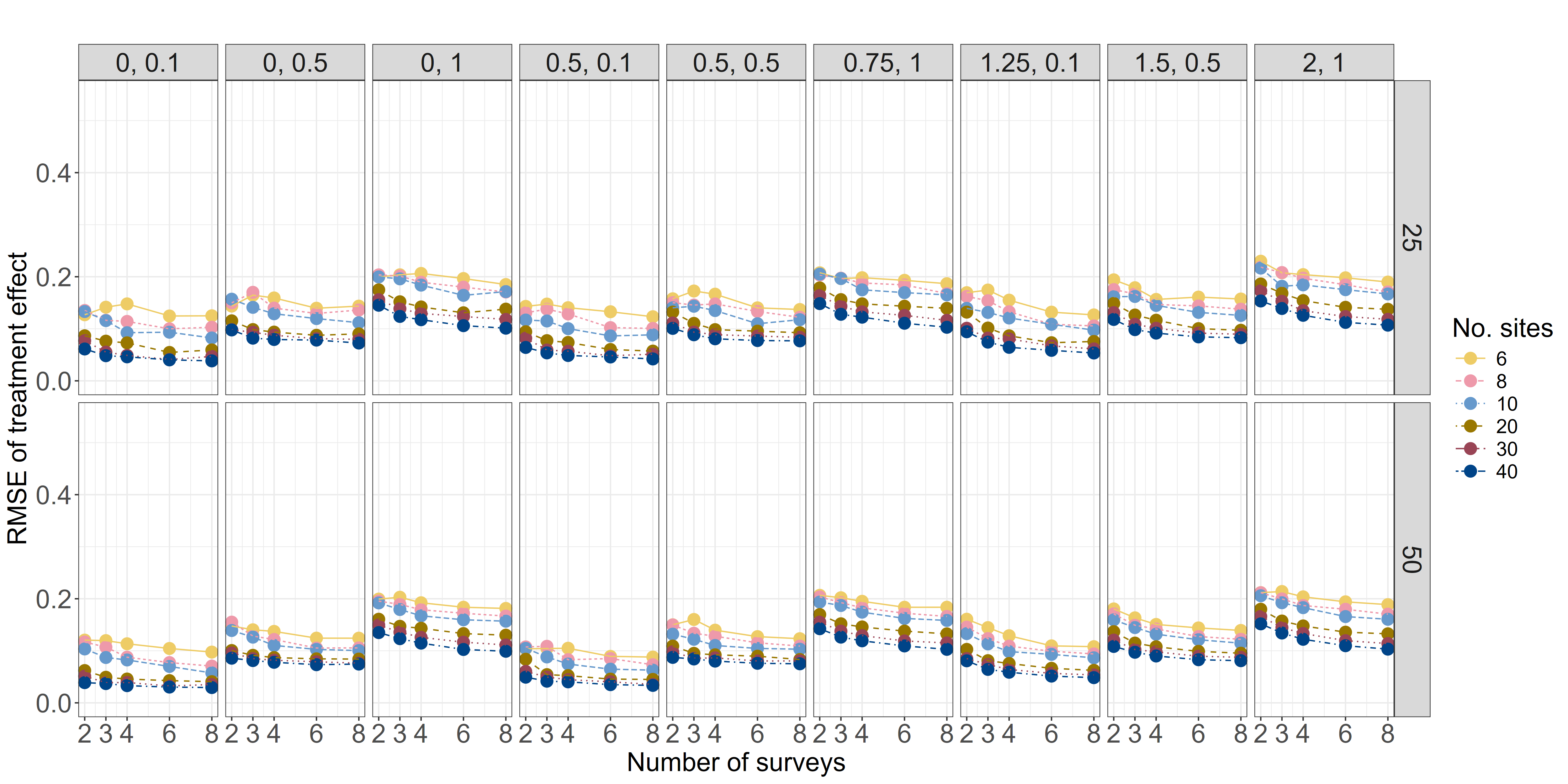


Figure 2b. Comparing root-mean squared error (RMSE) of estimated treatment effect for 100 replicates at each design X simulation combination under the multispecies occupancy model. Columns vary by the normal distribution parameters governing the treatment effect scenario. Rows vary by the number of species in the community. RMSE increased with larger treatment effect variance.

### All species - Hybrid


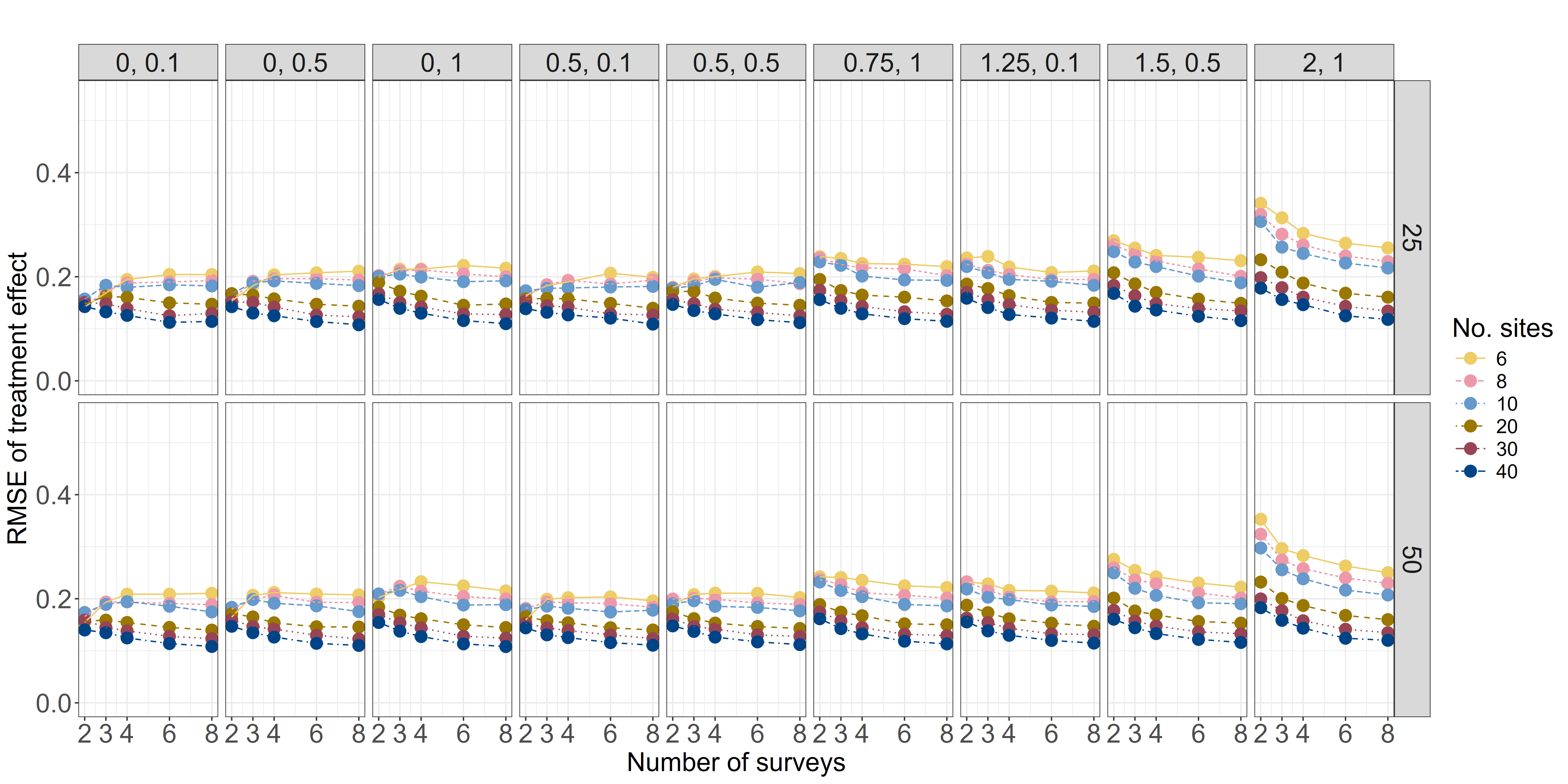


Figure 2c. Comparing root-mean squared error (RMSE) of estimated treatment effect for 100 replicates at each design X simulation combination under the hybrid model. Columns vary by the normal distribution parameters governing the treatment effect scenario. Rows vary by the number of species in the community. RMSE increased with larger treatment effect variance.

### Only rare species - SSOM


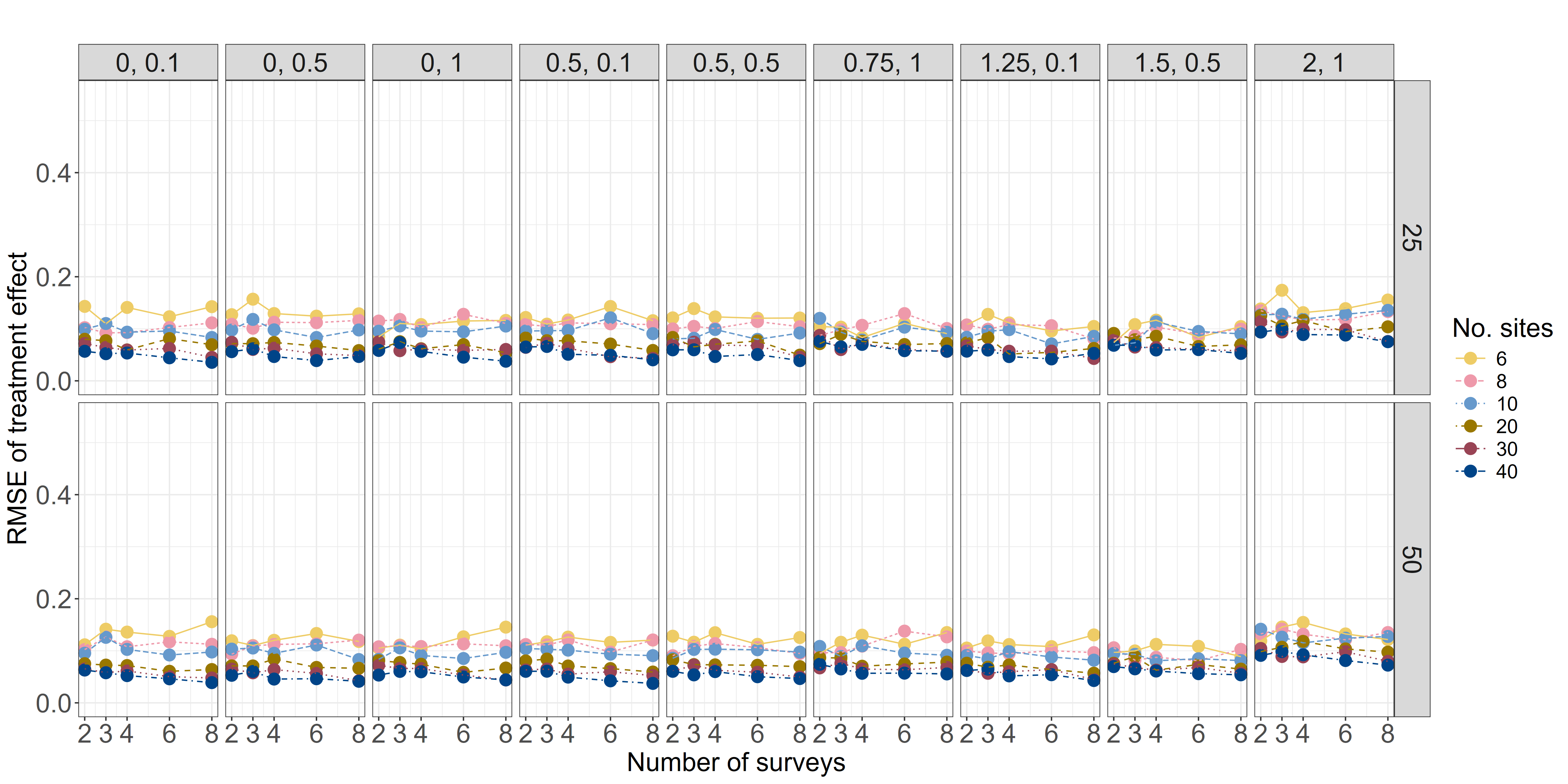


Figure 2d. Comparing root-mean squared error (RMSE) of estimated treatment effect for 100 replicates at each design X simulation combination when single-species occupancy models were fit for all species in the community (rare species only). Columns vary by the normal distribution parameters governing the treatment effect scenario. Rows vary by the number of species in the community. RMSE increased with larger treatment effect variance.

### Only rare species - MSOM


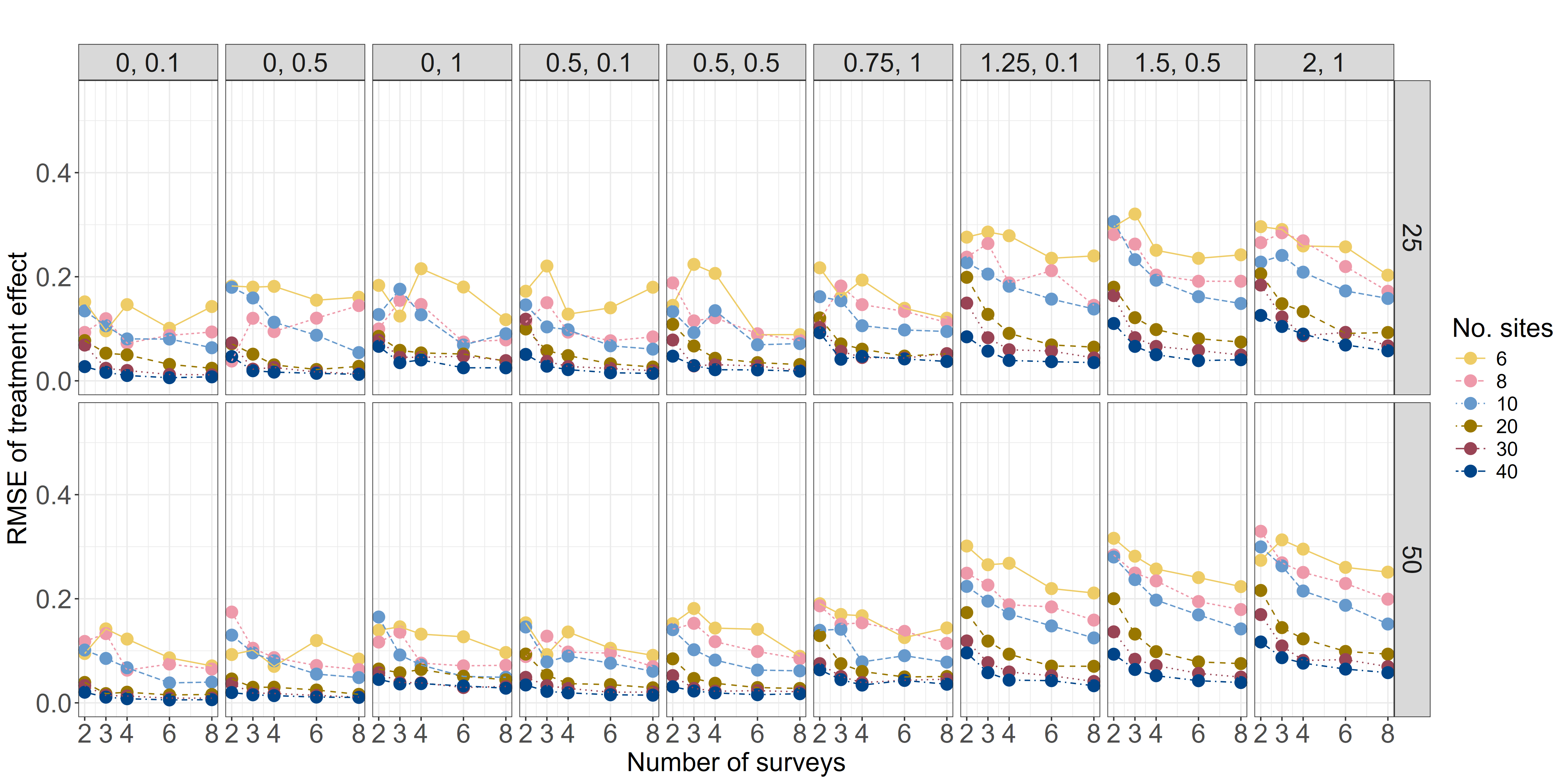


Figure 2e. Comparing root-mean squared error (RMSE) of estimated treatment effect for 100 replicates at each design X simulation combination under the multispecies occupancy model (rare species only). Columns vary by the normal distribution parameters governing the treatment effect scenario. Rows vary by the number of species in the community. RMSE increased with larger treatment effect variance.

### Only rare species - Hybrid


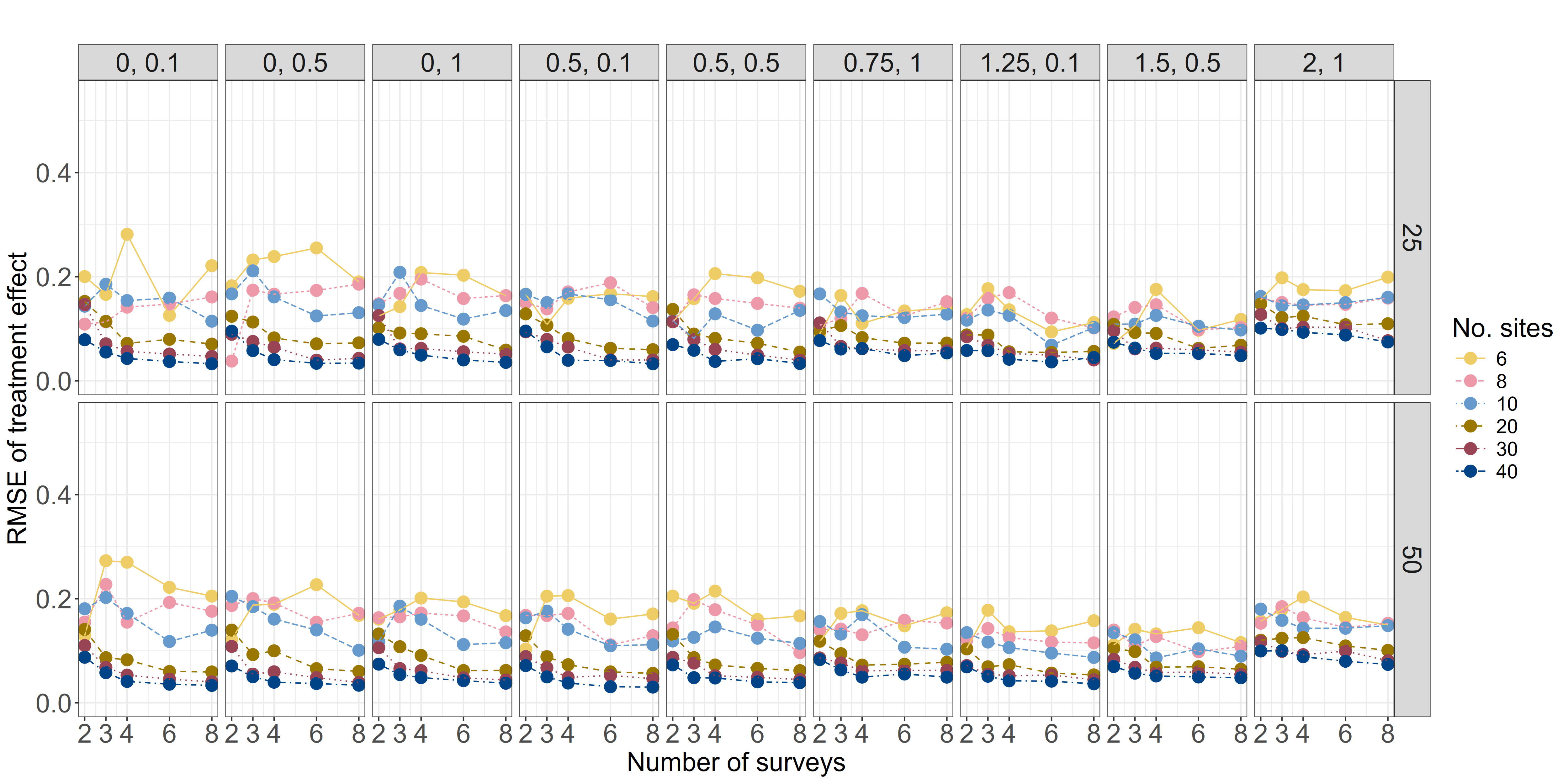


Figure 2f. Comparing root mean squared error of estimated treatment effect for 100 replicates at each design X simulation combination under the hybrid model (rare species only). Columns vary by the normal distribution parameters governing the treatment effect scenario. Rows vary by the number of species in the community.

## Coverage

Coverage was defined as the proportion of the time that the true treatment effect ($\hat{\lambda}_{k}$) fell within the 95% CI of treatment effect estimates ($\lambda_{k}$). Coverage was generally high, but decreased under the MSOM as variance increased. Coverage was low under the MSOM when considering only rare species, especially as mean effect magnitude increased.

### All species - SSOM


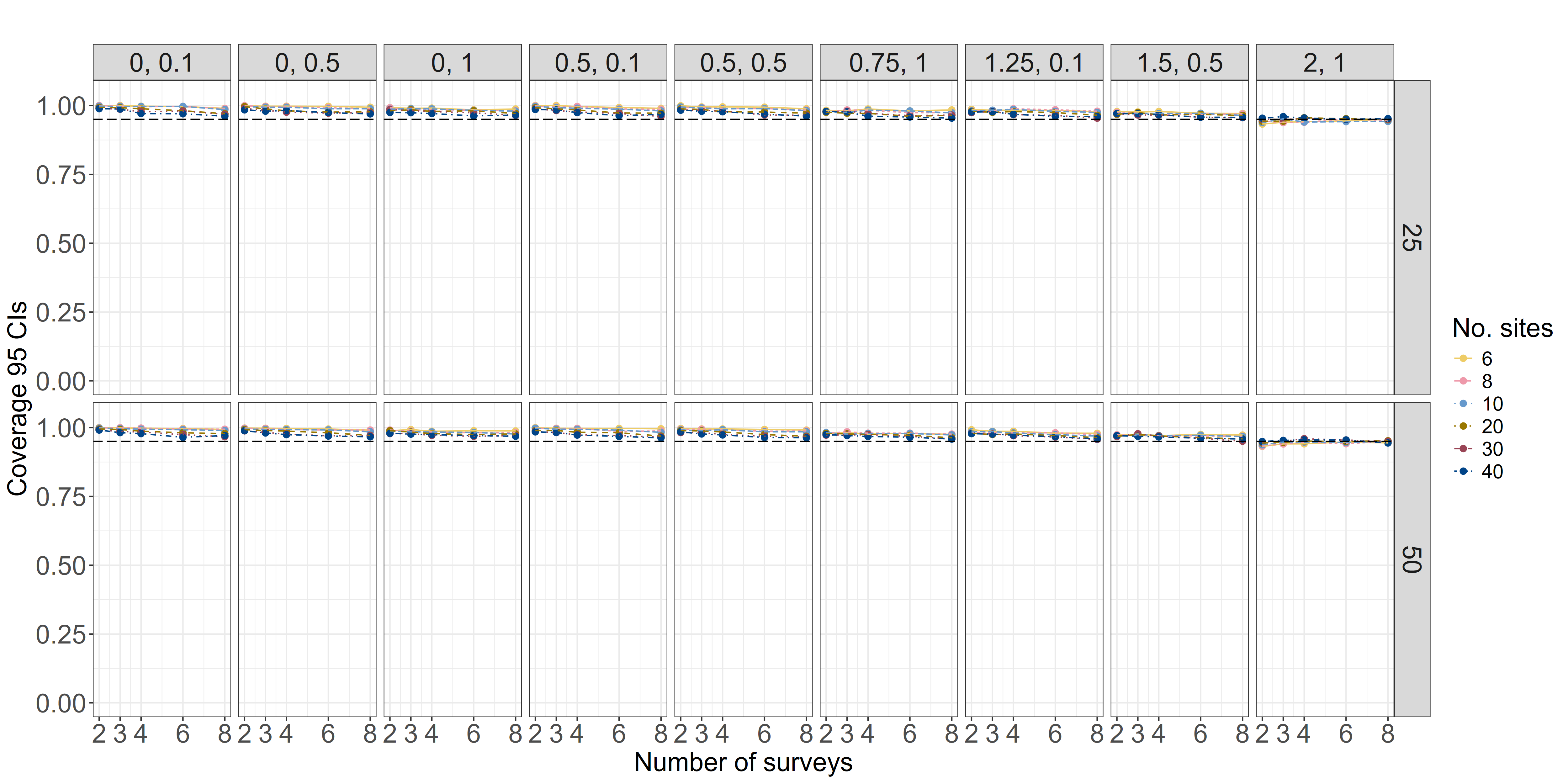


Figure 3a. 95% confidence interval coverage of treatment effect estimates under the single species occupancy model (SSOM). Columns vary by the normal distribution parameters governing the treatment effect scenario. Rows vary by the number of species in the community. Coverage tends to exceed the 95% threshold (dashed horizontal line) when estimates have low precision.

### All species - MSOM


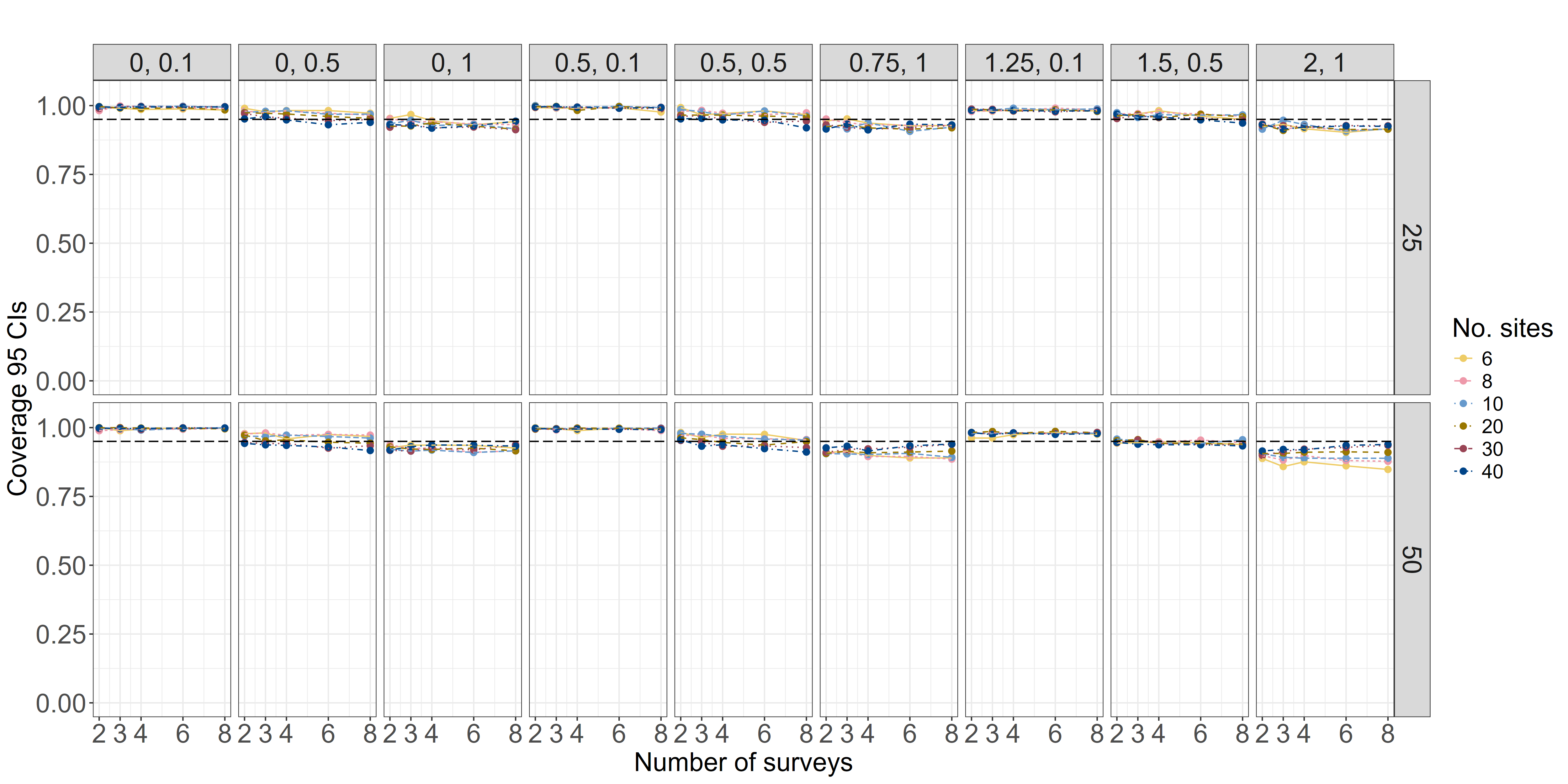
 Figure 3b. 95% confidence interval coverage of treatment effect estimates under the multispecies occupancy model (MSOM). Columns vary by the normal distribution parameters governing the treatment effect scenario. Rows vary by the number of species in the community. Coverage tends to exceed the 95% threshold (dashed horizontal line) when estimates have low precision.

### All species - Hybrid


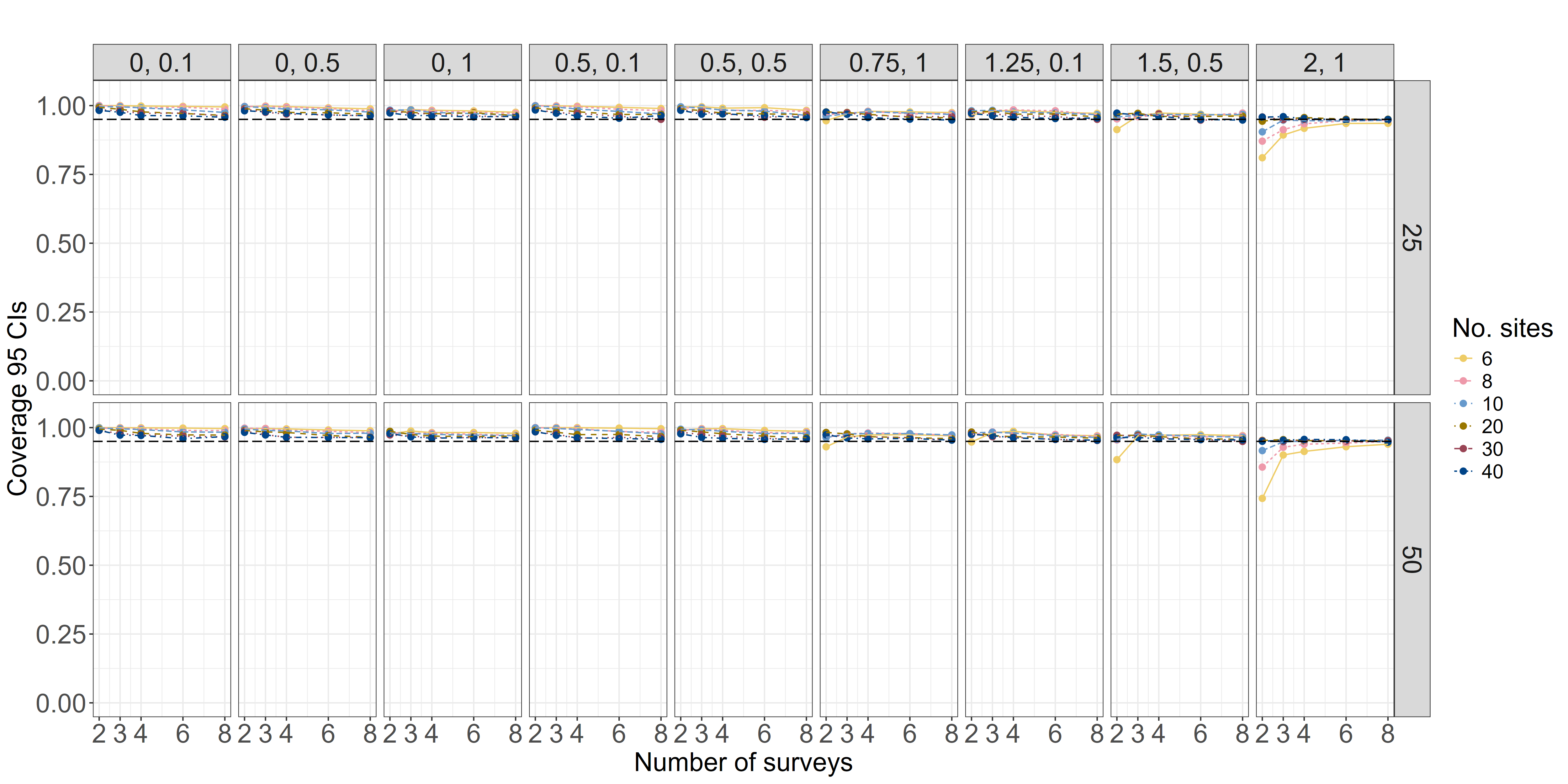
 Figure 3c. 95% confidence interval coverage of treatment effect estimates under the hybrid occupancy model. Columns vary by the normal distribution parameters governing the treatment effect scenario. Rows vary by the number of species in the community. Coverage tends to exceed the 95% threshold (dashed horizontal line) when estimates have low precision.

### Only rare species - SSOM


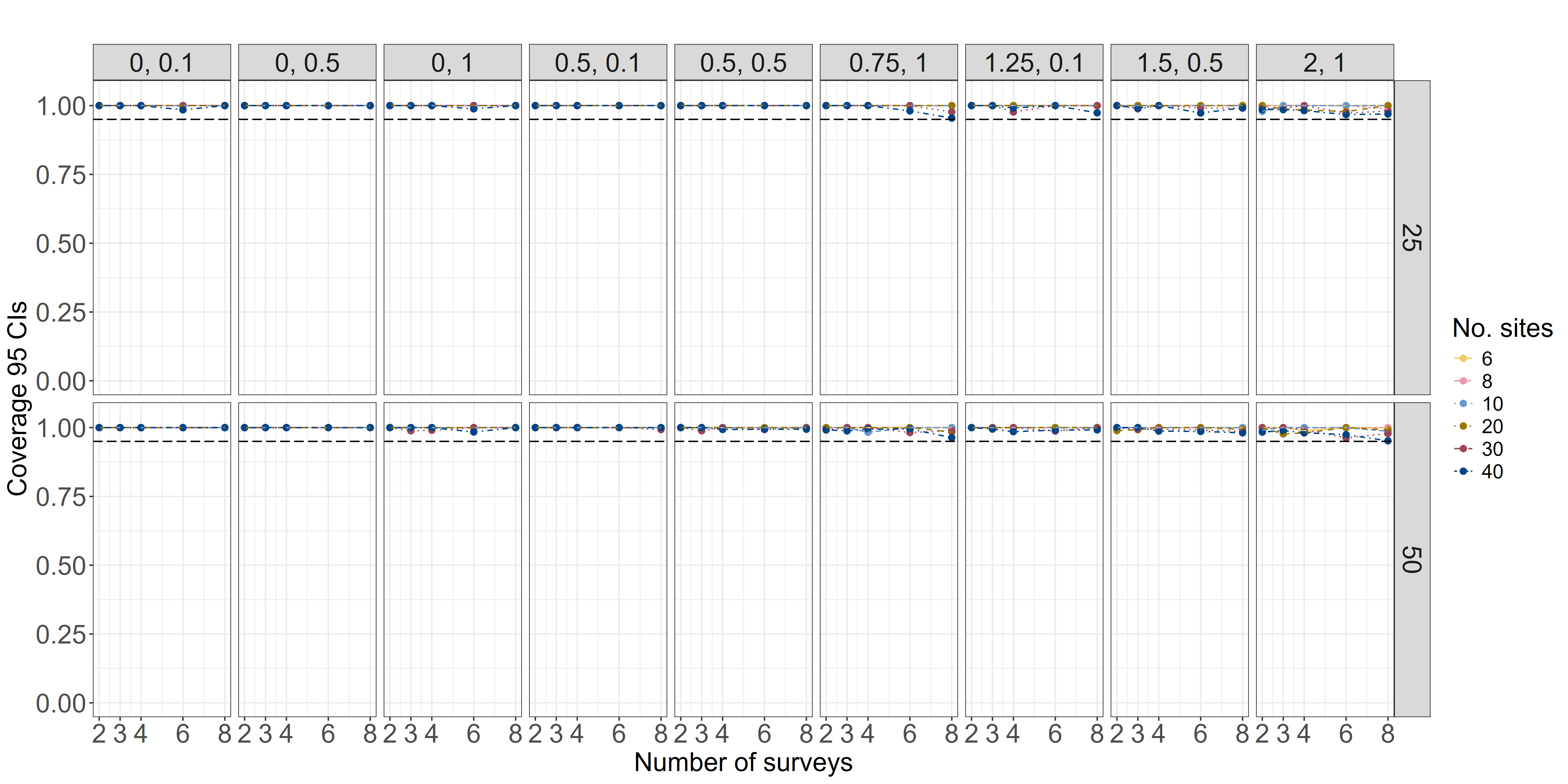


Figure 3d. 95% confidence interval coverage of treatment effect estimates under the single species occupancy model (SSOM) when only species of concern were considered. Columns vary by the normal distribution parameters governing the treatment effect scenario. Rows vary by the number of species in the community. Coverage tends to exceed the 95% threshold (dashed horizontal line) when estimates have low precision.

### Only rare species - MSOM


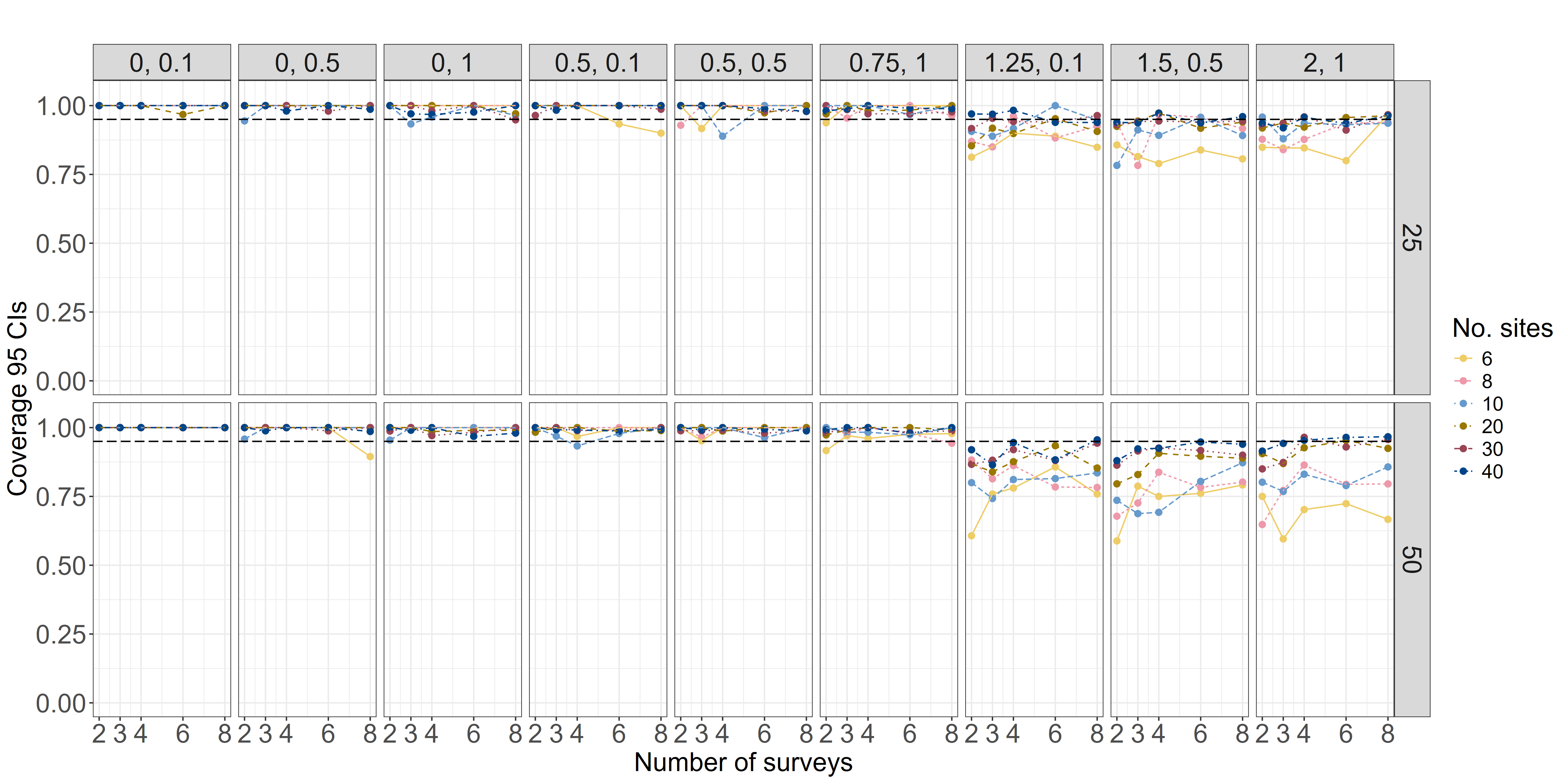


Figure 3e. 95% confidence interval coverage of treatment effect estimates under the multispecies occupancy model (MSOM) when only species of concern were considered. Columns vary by the normal distribution parameters governing the treatment effect scenario. Rows vary by the number of species in the community. Coverage tends to exceed the 95% threshold (dashed horizontal line) when estimates have low precision.

### Only rare species - Hybrid


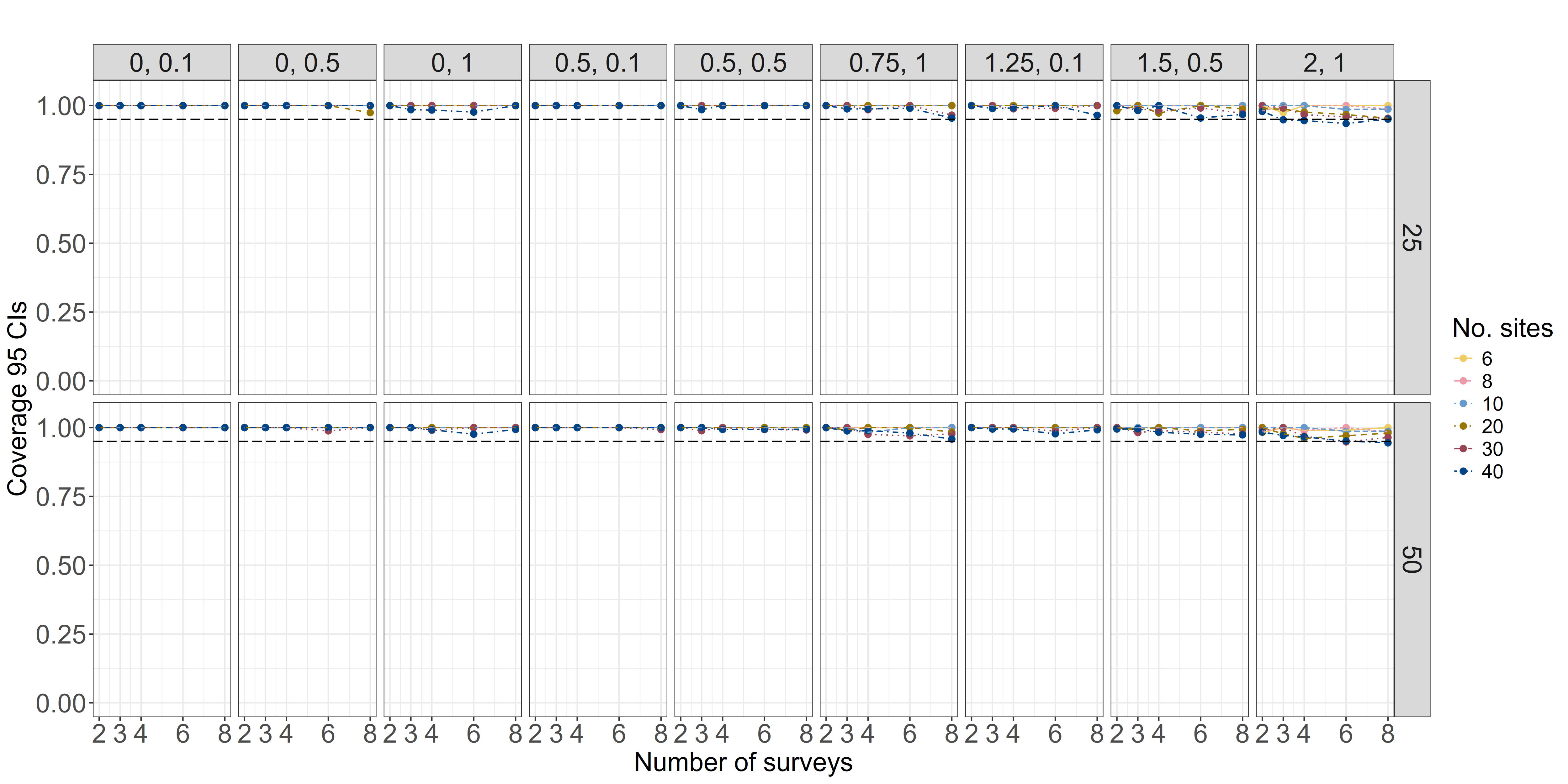


Figure 3f. 95% confidence interval coverage of treatment effect estimates under the hybrid occupancy model when only species of concern were considered. Columns vary by the normal distribution parameters governing the treatment effect scenario. Rows vary by the number of species in the community. Coverage tends to exceed the 95% threshold (dashed horizontal line) when estimates have low precision.

## CI widths

Coverage values were high because the confidence intervals were very wide. The widths mostly change as a function of sampling effort, and change less as a function of effect magnitude.

### All species - SSOM


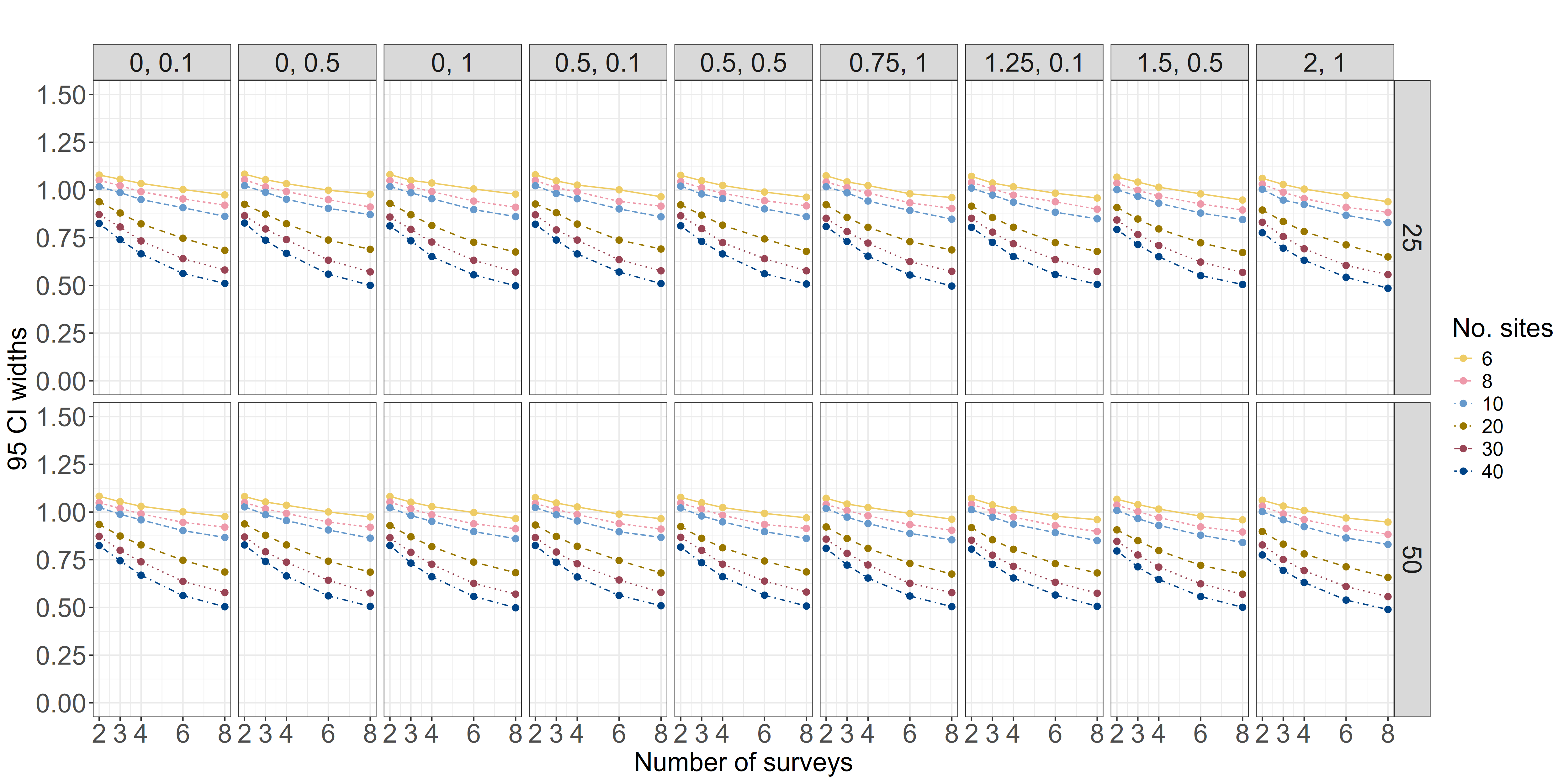


Figure 4a. 95% confidence interval widths (probability scale) for treatment effect estimates under the single species occupancy model (SSOM). Columns vary by the normal distribution parameters governing the treatment effect scenario. Rows vary by the number of species in the community.

### All species - MSOM


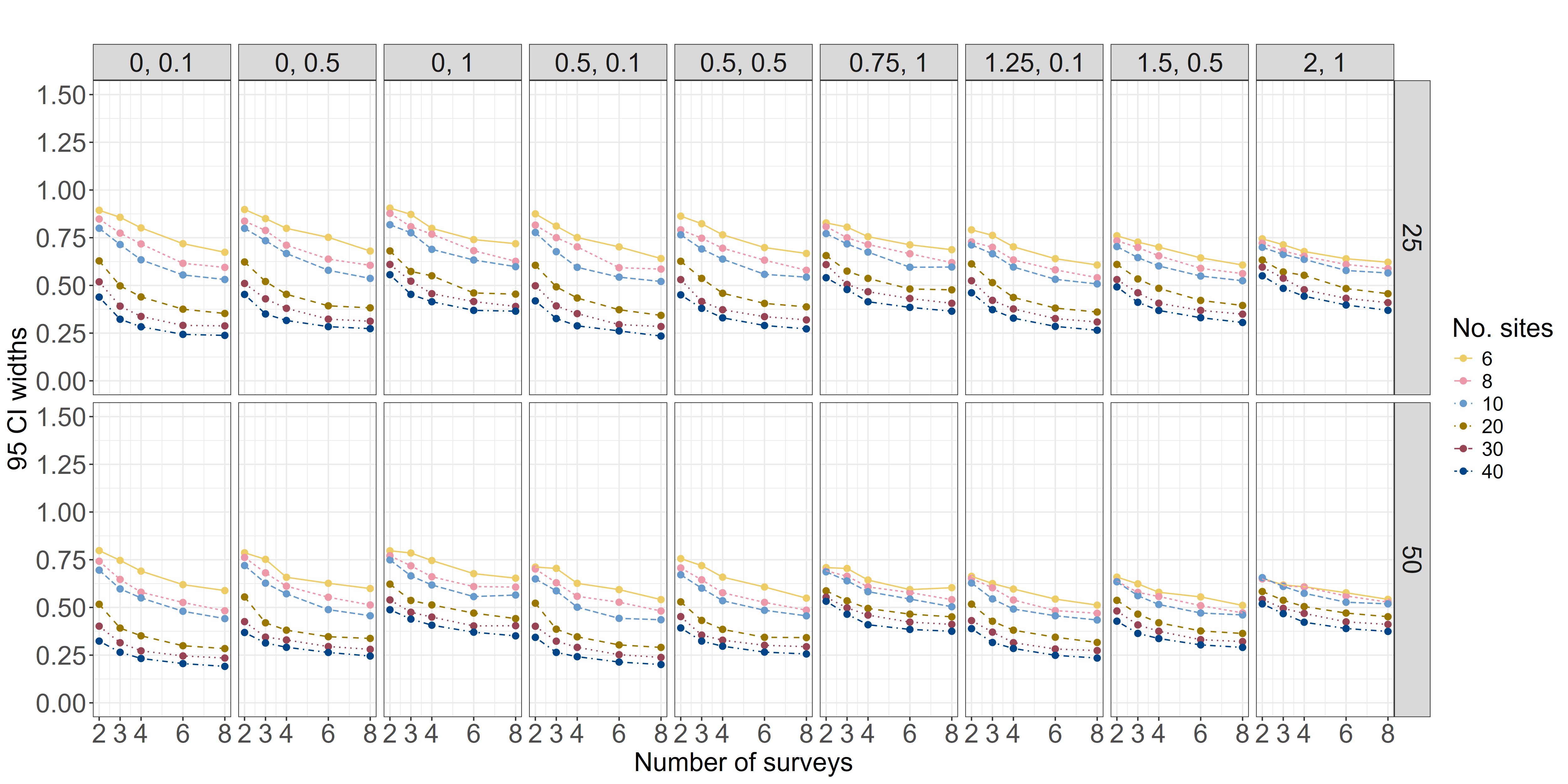


Figure 4b. 95% confidence interval widths (probability scale) for treatment effect estimates under the multispecies occupancy model (MSOM). Columns vary by the normal distribution parameters governing the treatment effect scenario. Rows vary by the number of species in the community.

### All species - Hybrid


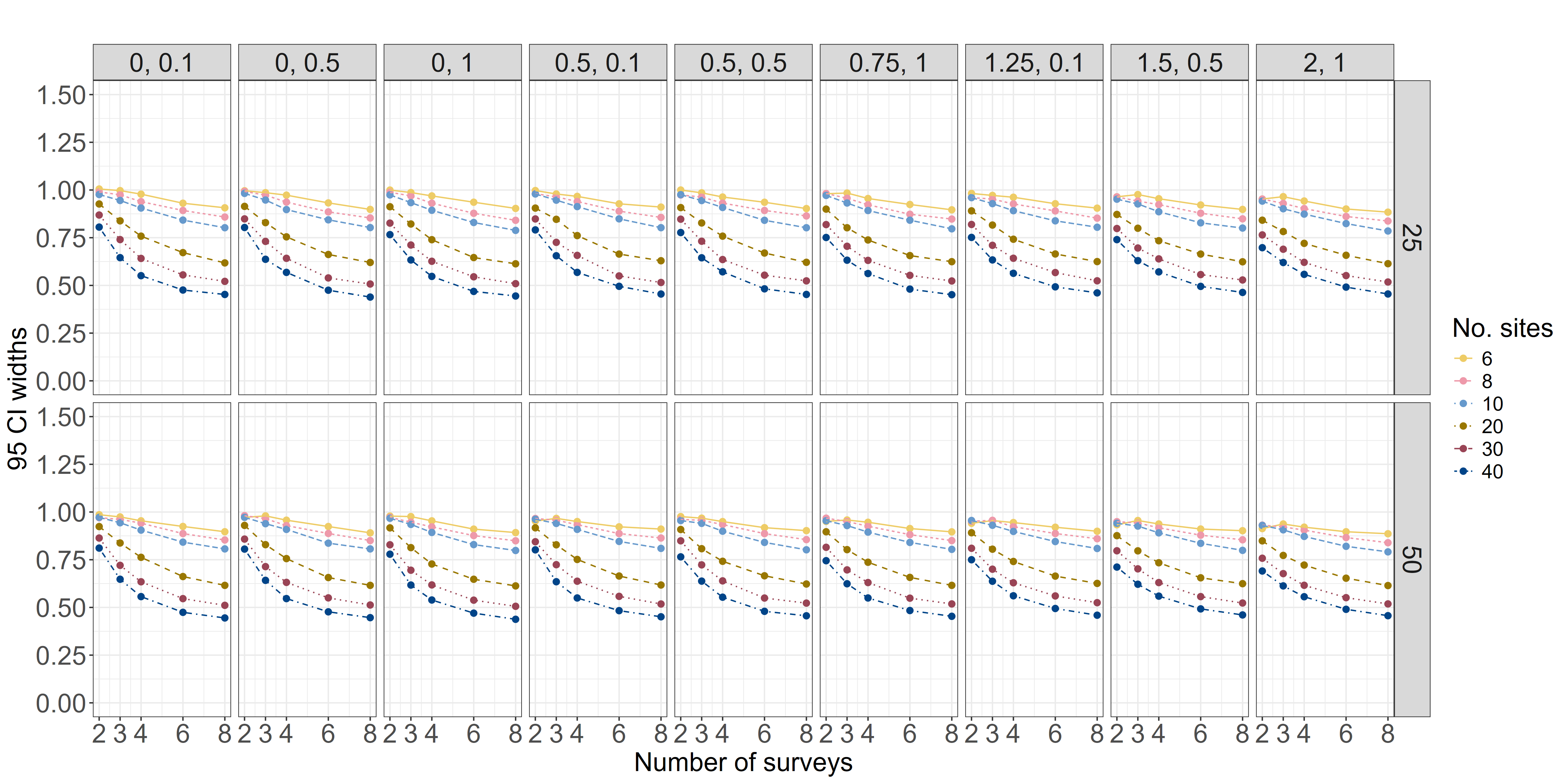


Figure 4c. 95% confidence interval widths (probability scale) for treatment effect estimates under the hybrid occupancy model. Columns vary by the normal distribution parameters governing the treatment effect scenario. Rows vary by the number of species in the community.

### Only rare species - SSOM


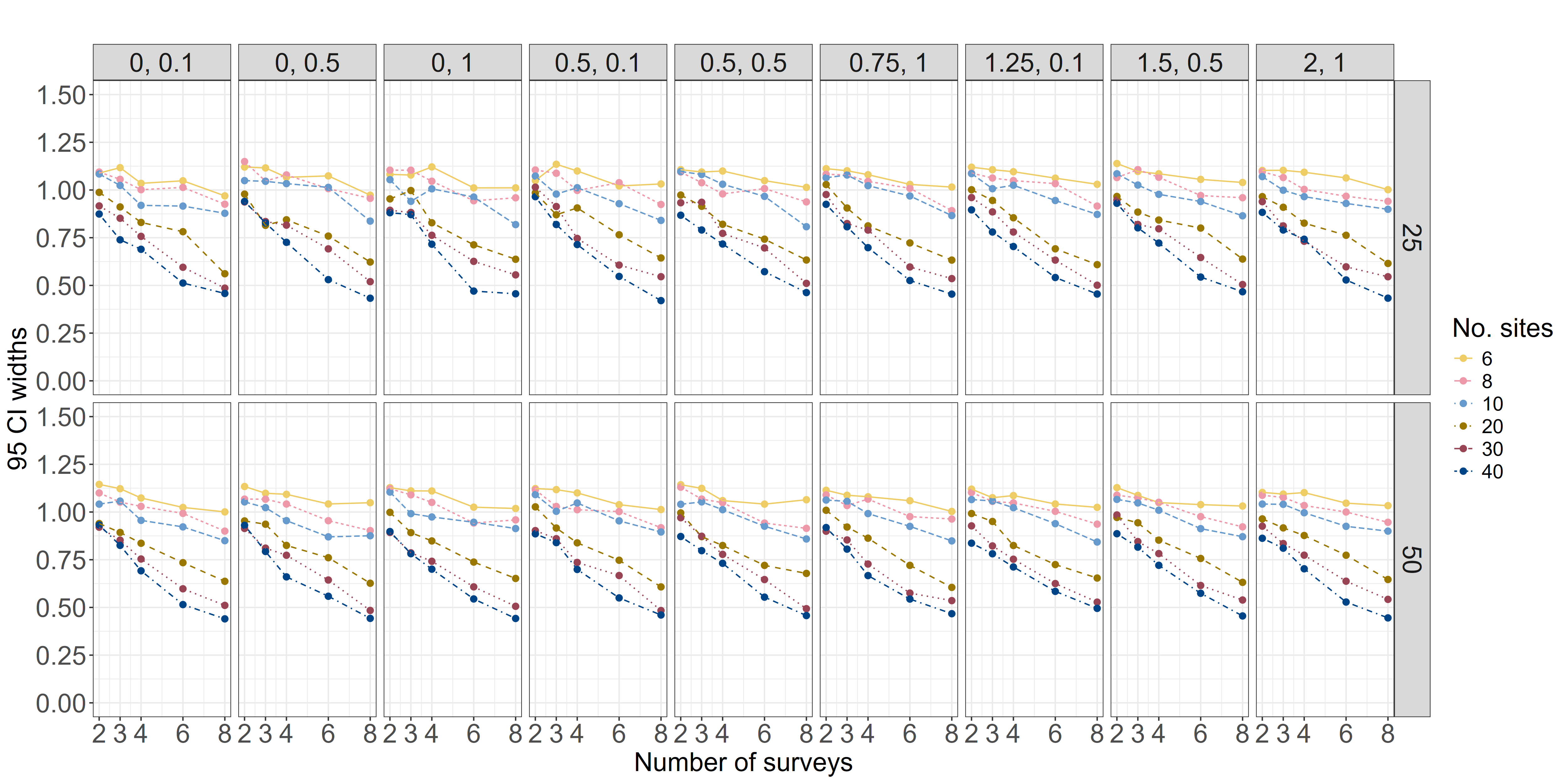


Figure 4d. 95% confidence interval widths (probability scale) for treatment effect estimates under the single species occupancy model (SSOM) when only species of concern were considered. Columns vary by the normal distribution parameters governing the treatment effect scenario. Rows vary by the number of species in the community.

### Only rare species - MSOM


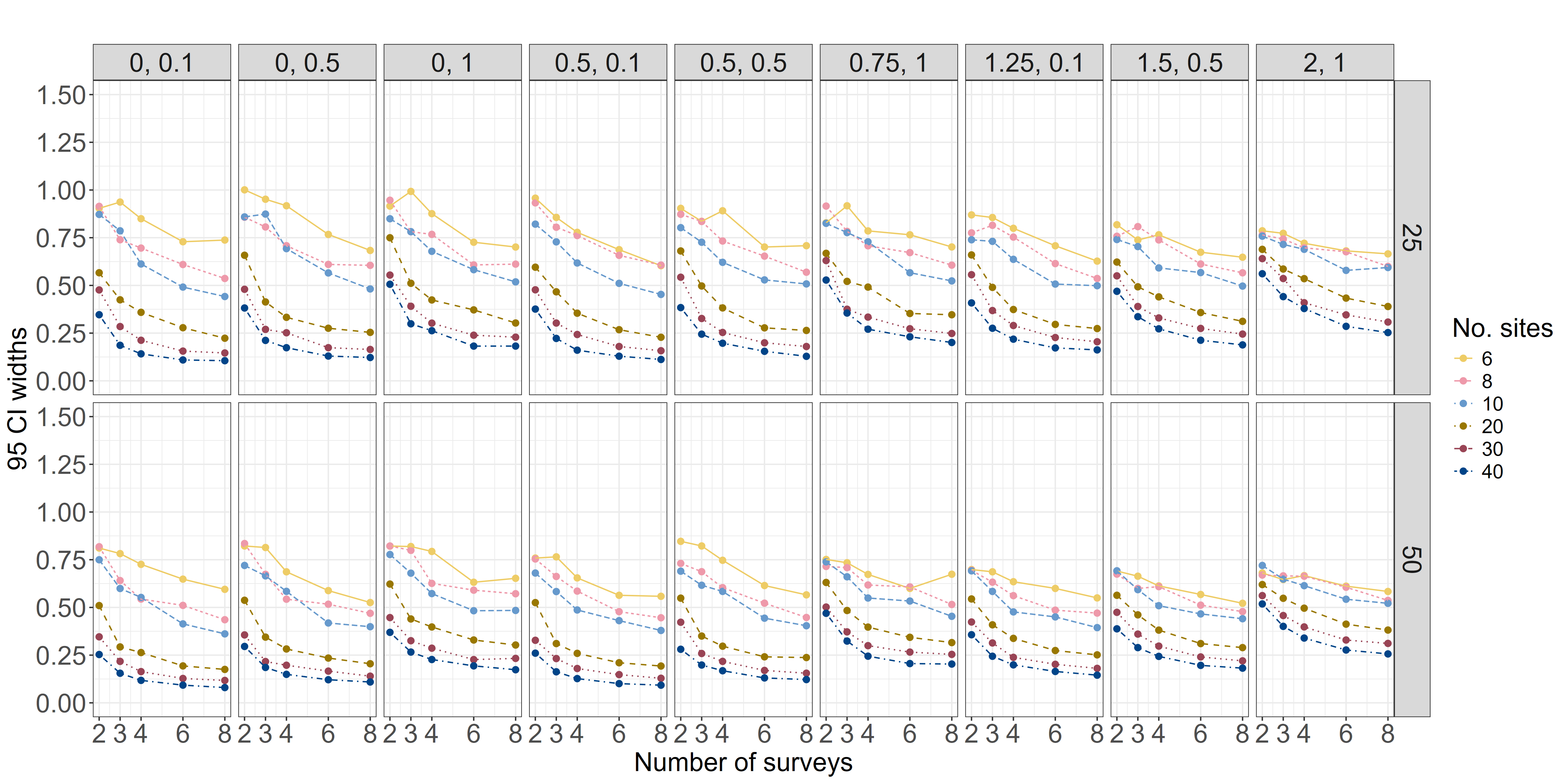


Figure 4e. 95% confidence interval widths (probability scale) for treatment effect estimates under the multispecies occupancy model (MSOM) when only species of concern were considered. Columns vary by the normal distribution parameters governing the treatment effect scenario. Rows vary by the number of species in the community.

### Only rare species - Hybrid


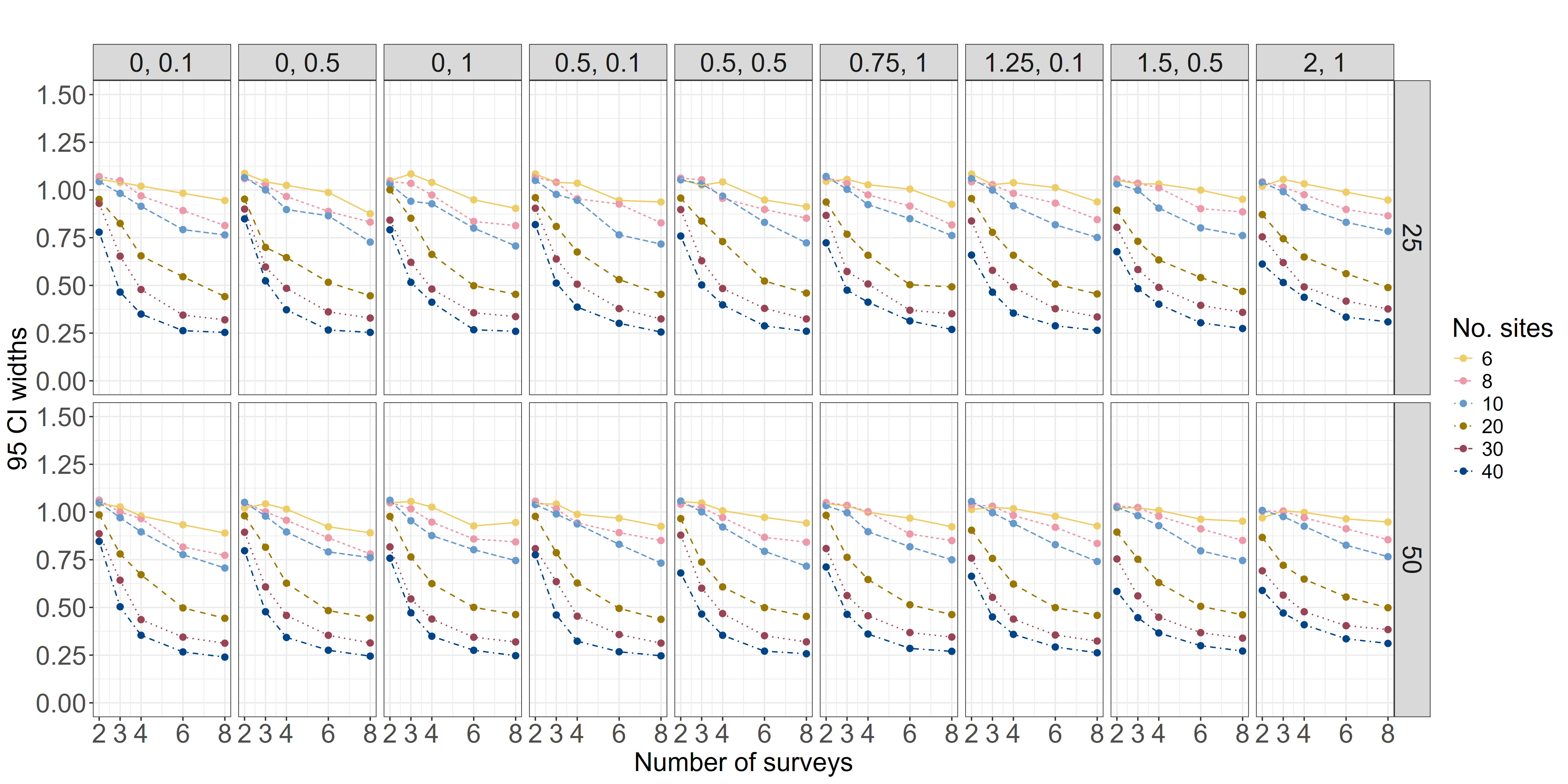


Figure 4f. 95% confidence interval widths (probability scale) for treatment effect estimates under the hybrid occupancy model when only species of concern were considered. Columns vary by the normal distribution parameters governing the treatment effect scenario. Rows vary by the number of species in the community.

## Type 1 error risk at 95% CI threshold

The proportion of the time that the 95% CI of estimated treatment effect ($\hat{\lambda}_{k}$) does not overlap zero when the true species-specific treatment effects were negligble ($-0.01<\lambda_{k}<0.01$). Type 1 error risk is low when estimates are imprecise. In large average effect magnitude scenarios, fewer simulated species experience a negligible treatment effect. In these scenarios under the MSOM, but not the SSOM or hybrid models, type 1 error increased.

### All species - SSOM


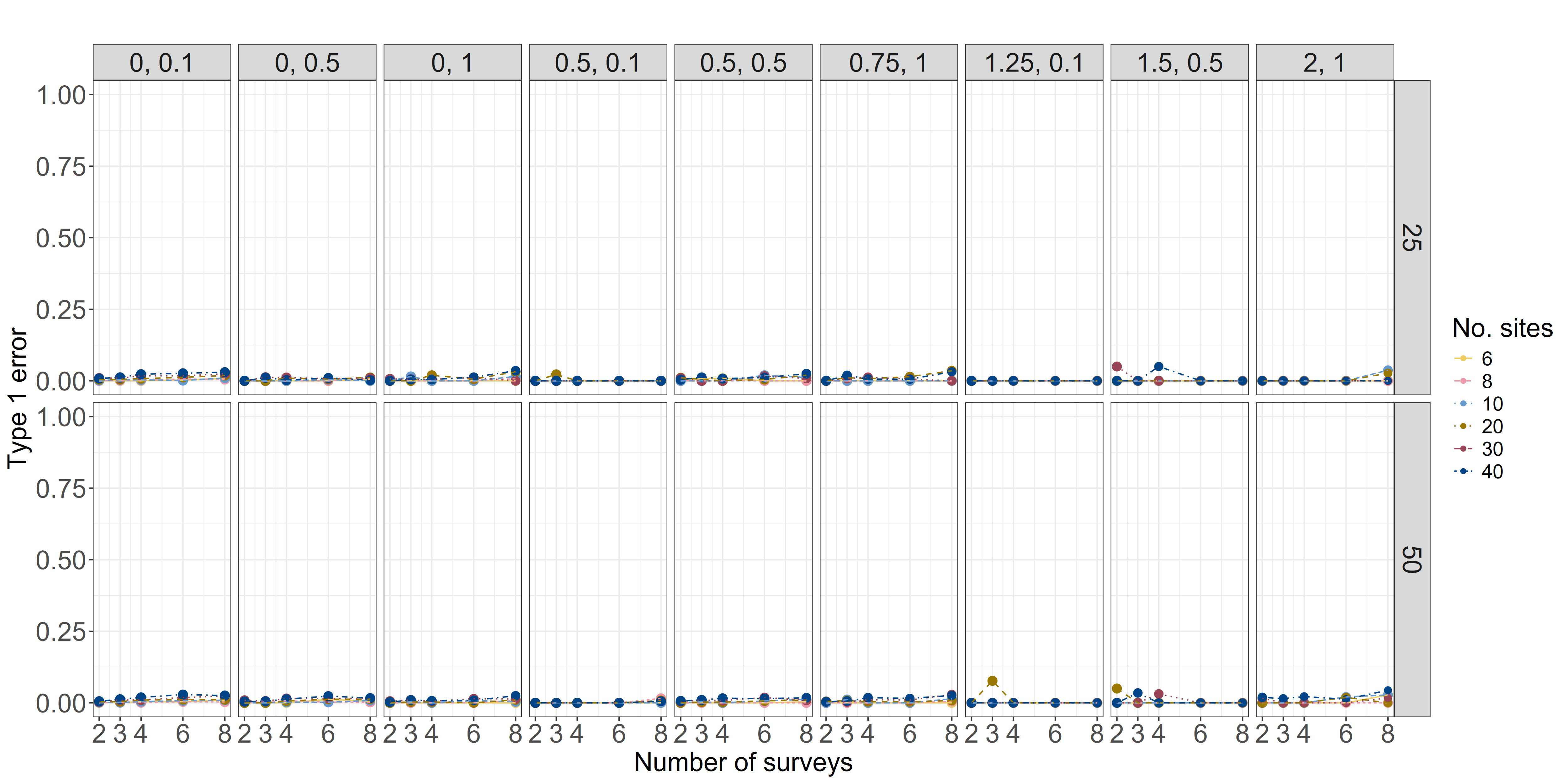


Figure 5a. Type 1 error risk under the single species occupancy model (SSOM). Columns vary by the normal distribution parameters governing the treatment effect scenario. Rows vary by the number of species in the community. The number of species experiencing no treatment effect decreased as the mean effect magnitude increased (left to right). Point sizes reflect the relative proportion of the sample experiencing no treatment effect.

### All species - MSOM


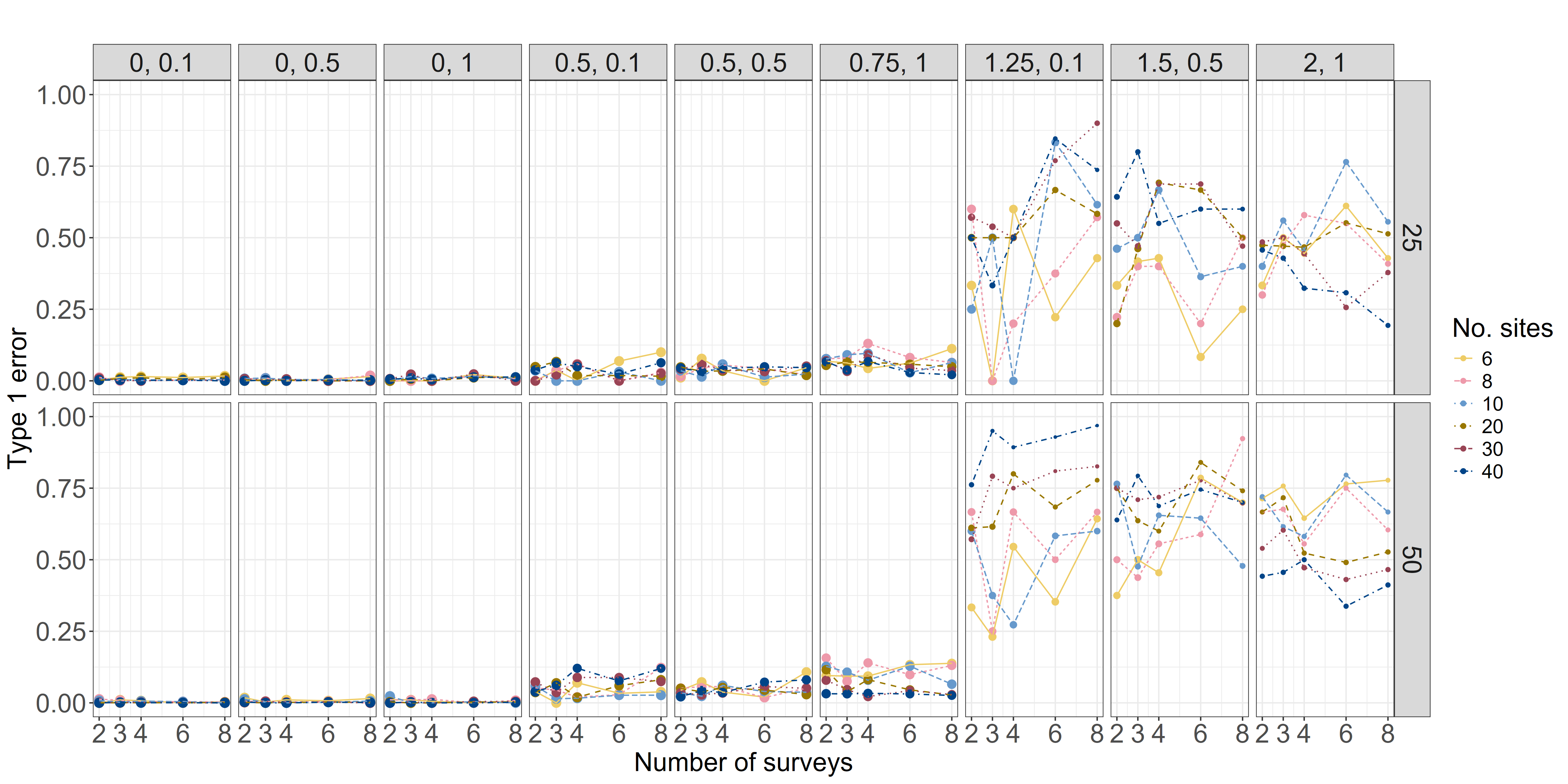


Figure 5b. Type 1 error risk under the multispecies occupancy model (MSOM). Columns vary by the normal distribution parameters governing the treatment effect scenario. Rows vary by the number of species in the community. The number of species experiencing no treatment effect decreased as the mean effect magnitude increased (left to right). Point sizes reflect the relative proportion of the sample experiencing no treatment effect.

### All species - Hybrid


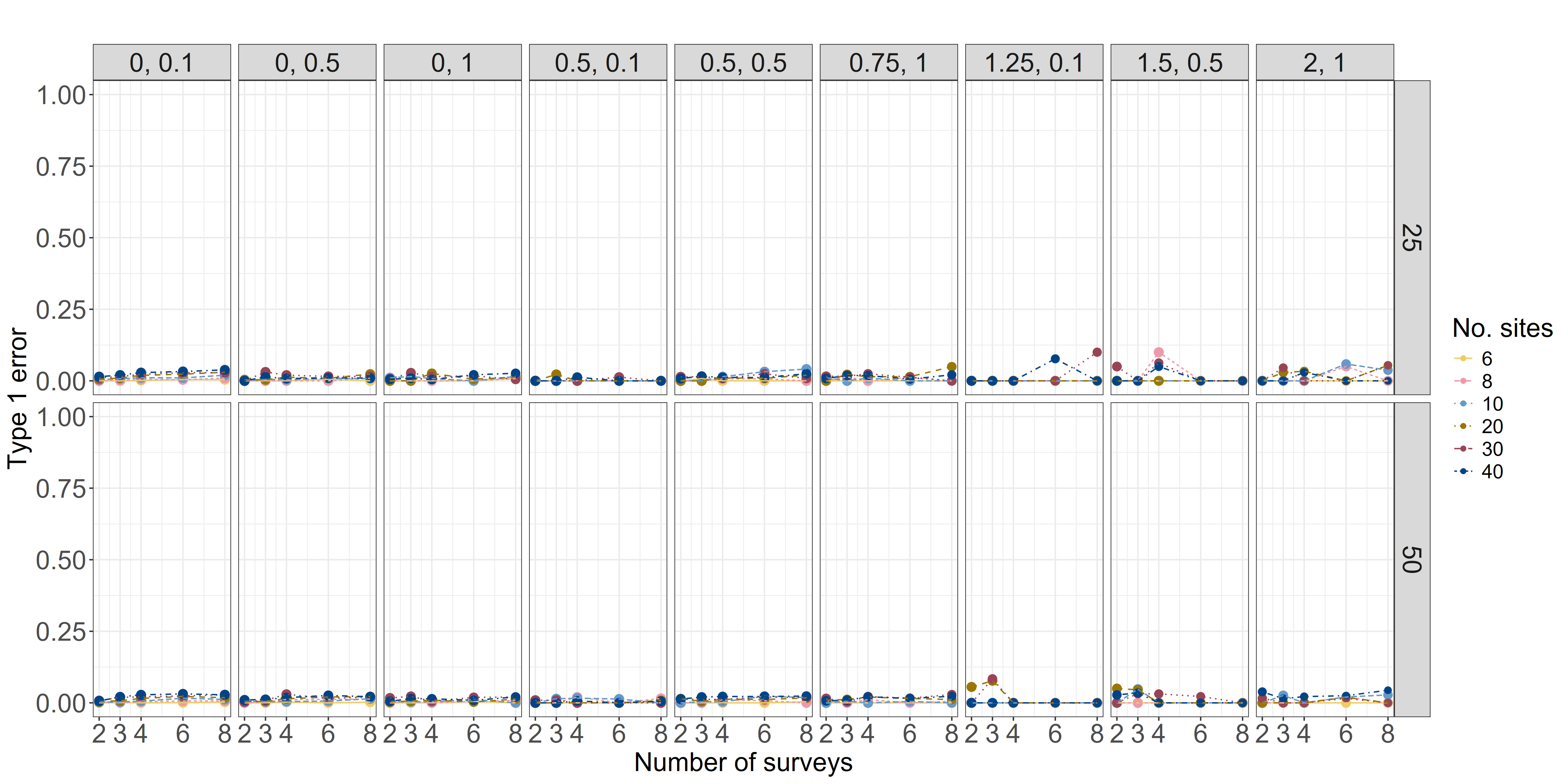


Figure 5c. Type 1 error risk under the hybrid occupancy model. Columns vary by the normal distribution parameters governing the treatment effect scenario. Rows vary by the number of species in the community. The number of species experiencing no treatment effect decreased as the mean effect magnitude increased (left to right). Point sizes reflect the relative proportion of the sample experiencing no treatment effect.

### Only rare species - SSOM


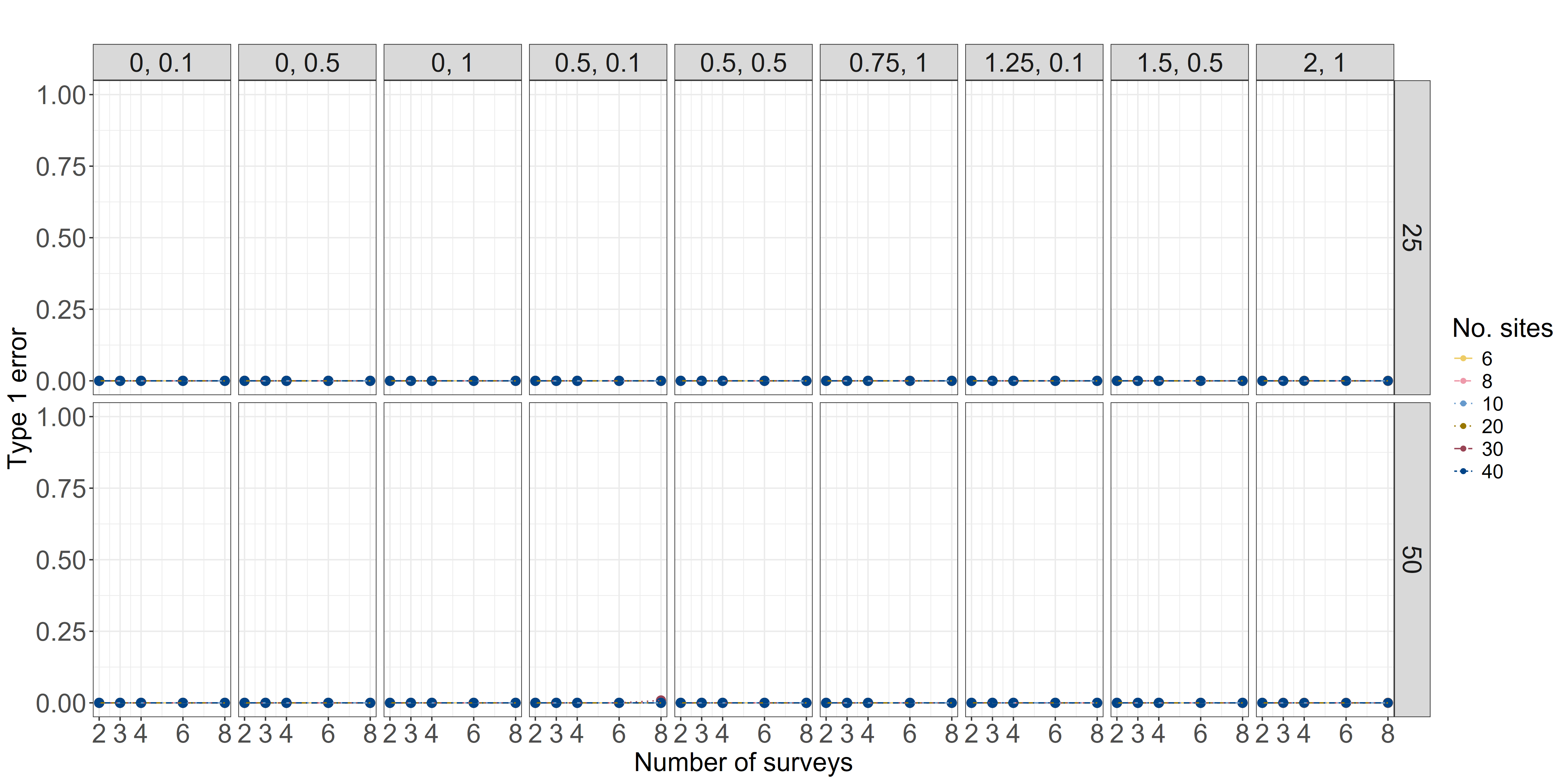


Figure 5d. Type 1 error risk under the single species occupancy model (SSOM) when only species of concern were considered. Columns vary by the normal distribution parameters governing the treatment effect scenario. Rows vary by the number of species in the community. The number of species experiencing no treatment effect decreased as the mean effect magnitude increased (left to right). Point sizes reflect the relative proportion of the sample experiencing no treatment effect.

### Only rare species - MSOM


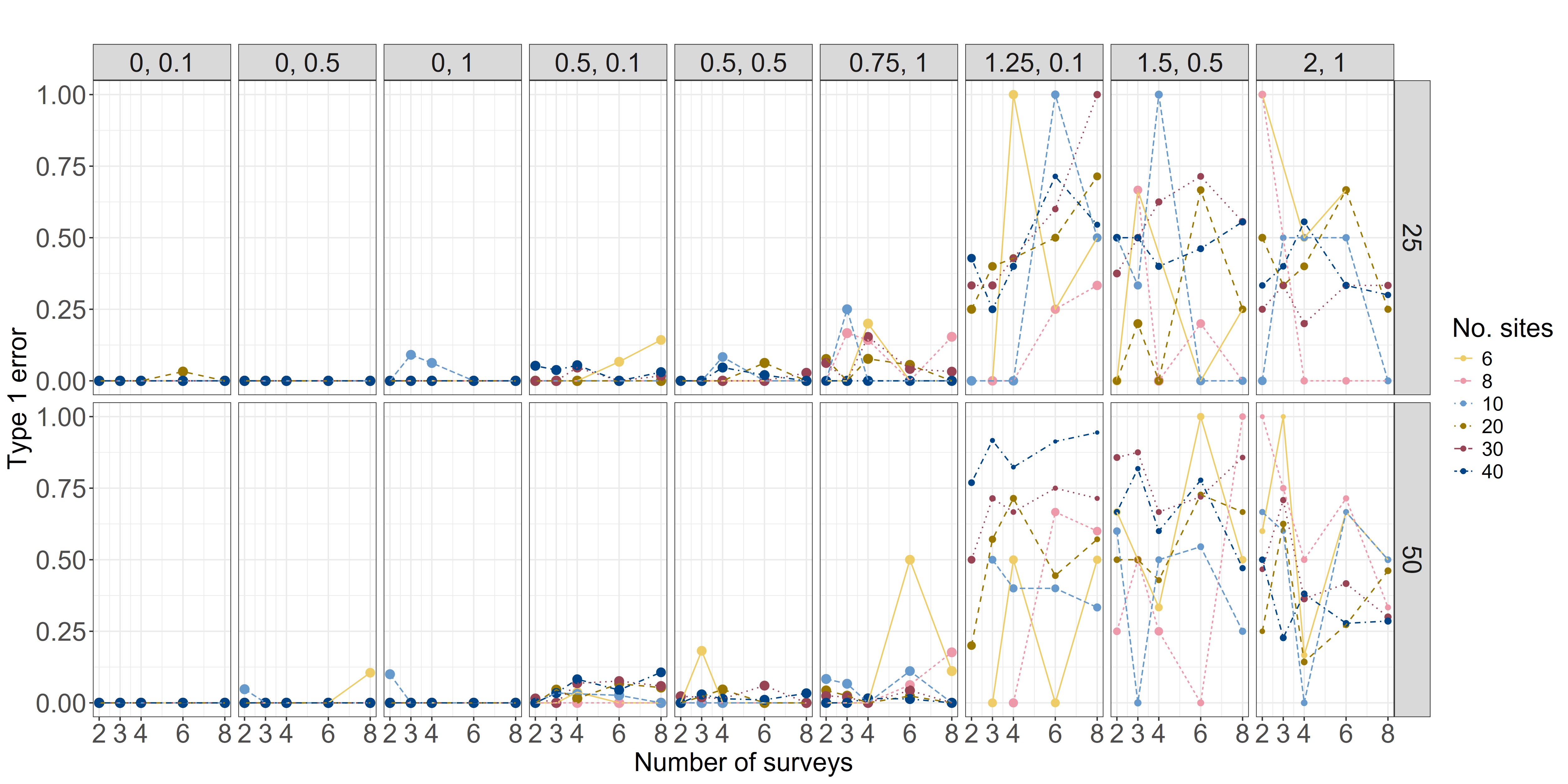


Figure 5e. Type 1 error risk under the multispecies occupancy model (MSOM) when only species of concern were considered. Columns vary by the normal distribution parameters governing the treatment effect scenario. Rows vary by the number of species in the community. The number of species experiencing no treatment effect decreased as the mean effect magnitude increased (left to right). Point sizes reflect the relative proportion of the sample experiencing no treatment effect.

### Only rare species - Hybrid


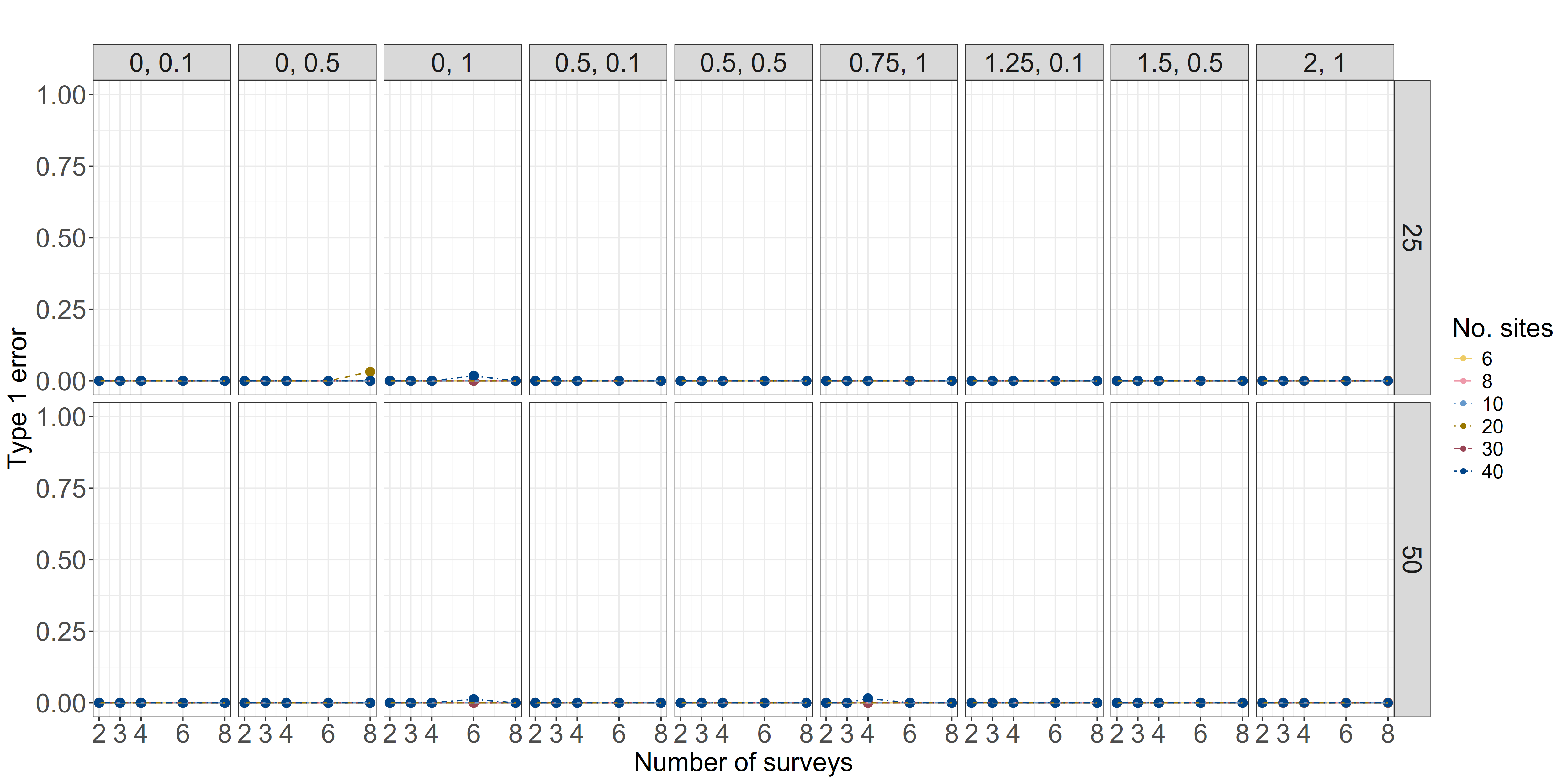


Figure 5f. Type 1 error risk under the hybrid occupancy model when only species of concern were considered. Columns vary by the normal distribution parameters governing the treatment effect scenario. Rows vary by the number of species in the community. The number of species experiencing no treatment effect decreased as the mean effect magnitude increased (left to right). Point sizes reflect the relative proportion of the sample experiencing no treatment effect.

## Type 2 error risk at 95% CI threshold

Type 2 error reflects the proportion of the time that the 95% CI of estimated treatment effect ($\hat{\lambda}_{k}$) overlaps zero when there is a true effect ($\hat{\lambda}_{k}<-0.01$ or $\hat{\lambda}_{k}>0.01$) in the simulated data. More precise species-specific estimates result in lower type 2 error, which is lowest under the MSOM. Type 2 error tends to be lowest when type 1 error is highest, based on the precision of estimates.

### All species - SSOM


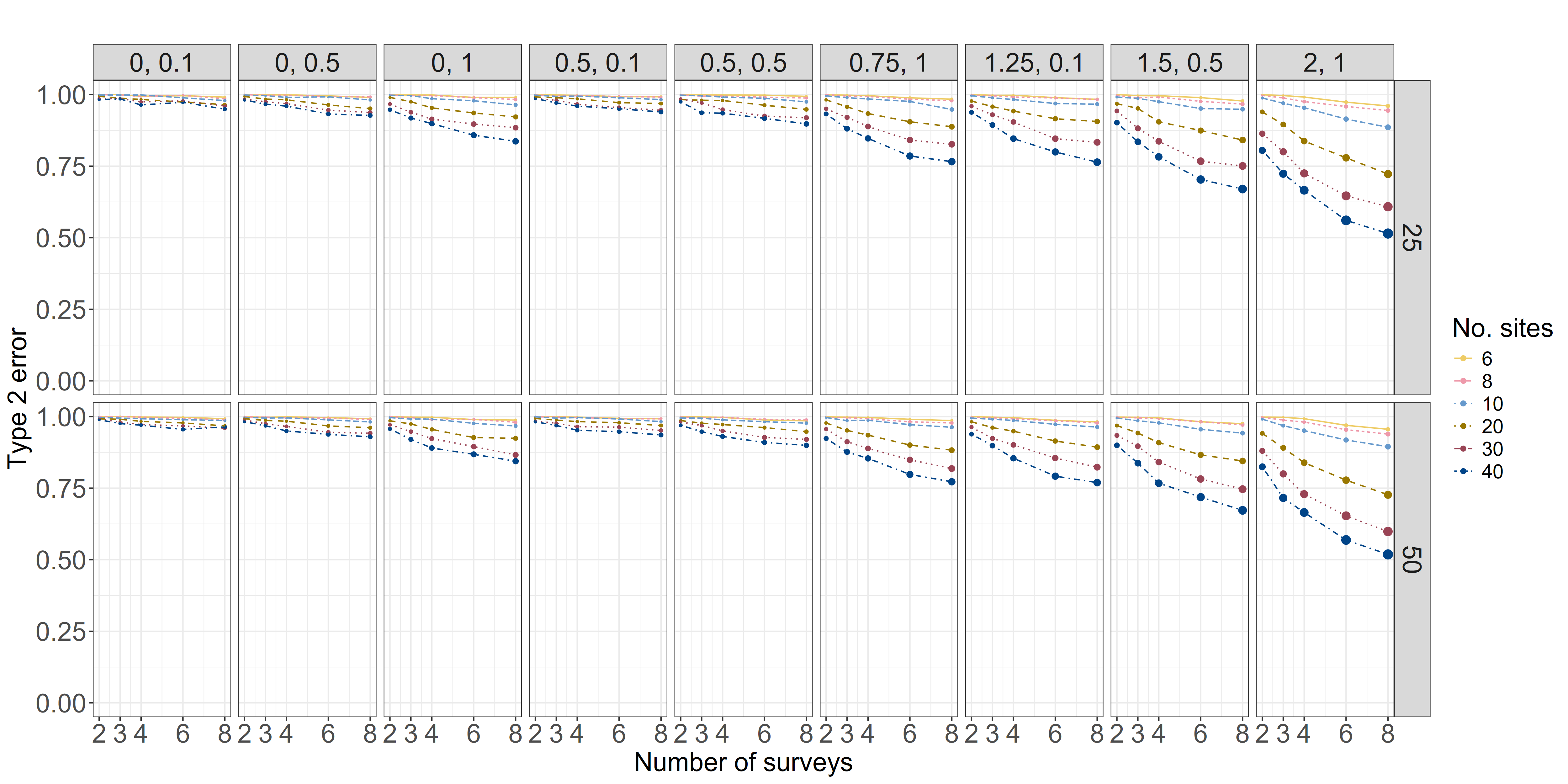


Figure 6a. Type 2 error risk under the single species occupancy model (SSOM). Columns vary by the normal distribution parameters governing the treatment effect scenario. Rows vary by the number of species in the community. The number of species experiencing non-negligble treatment effect increased as the mean effect magnitude increased (left to right). Point sizes reflect the relative proportion of the sample experiencing some treatment effect.

### All species - MSOM


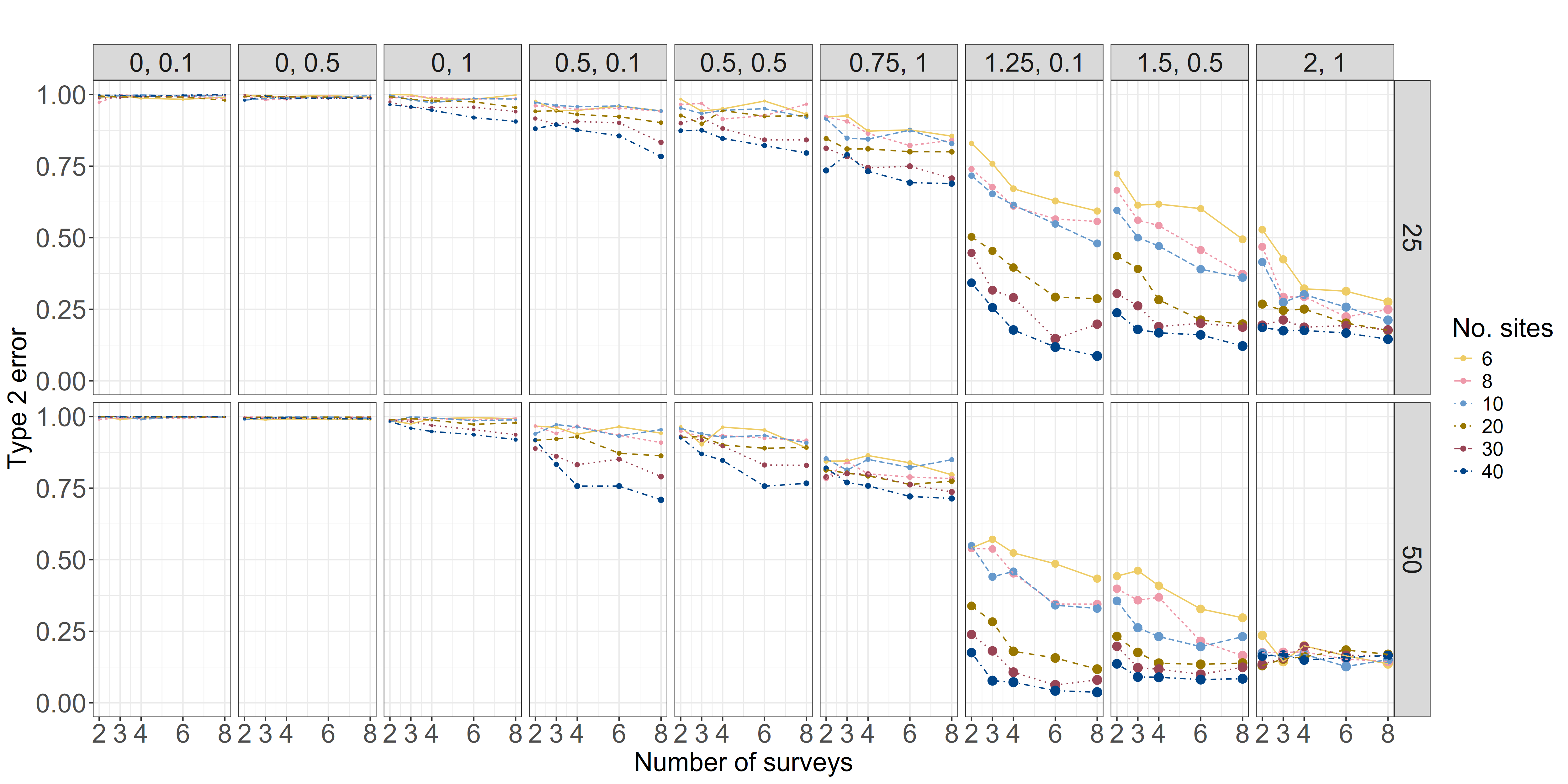


Figure 6b. Type 2 error risk under the multispecies occupancy model (MSOM). Columns vary by the normal distribution parameters governing the treatment effect scenario. Rows vary by the number of species in the community. The number of species experiencing non-negligble treatment effect increased as the mean effect magnitude increased (left to right). Point sizes reflect the relative proportion of the sample experiencing some treatment effect.

### All species - Hybrid


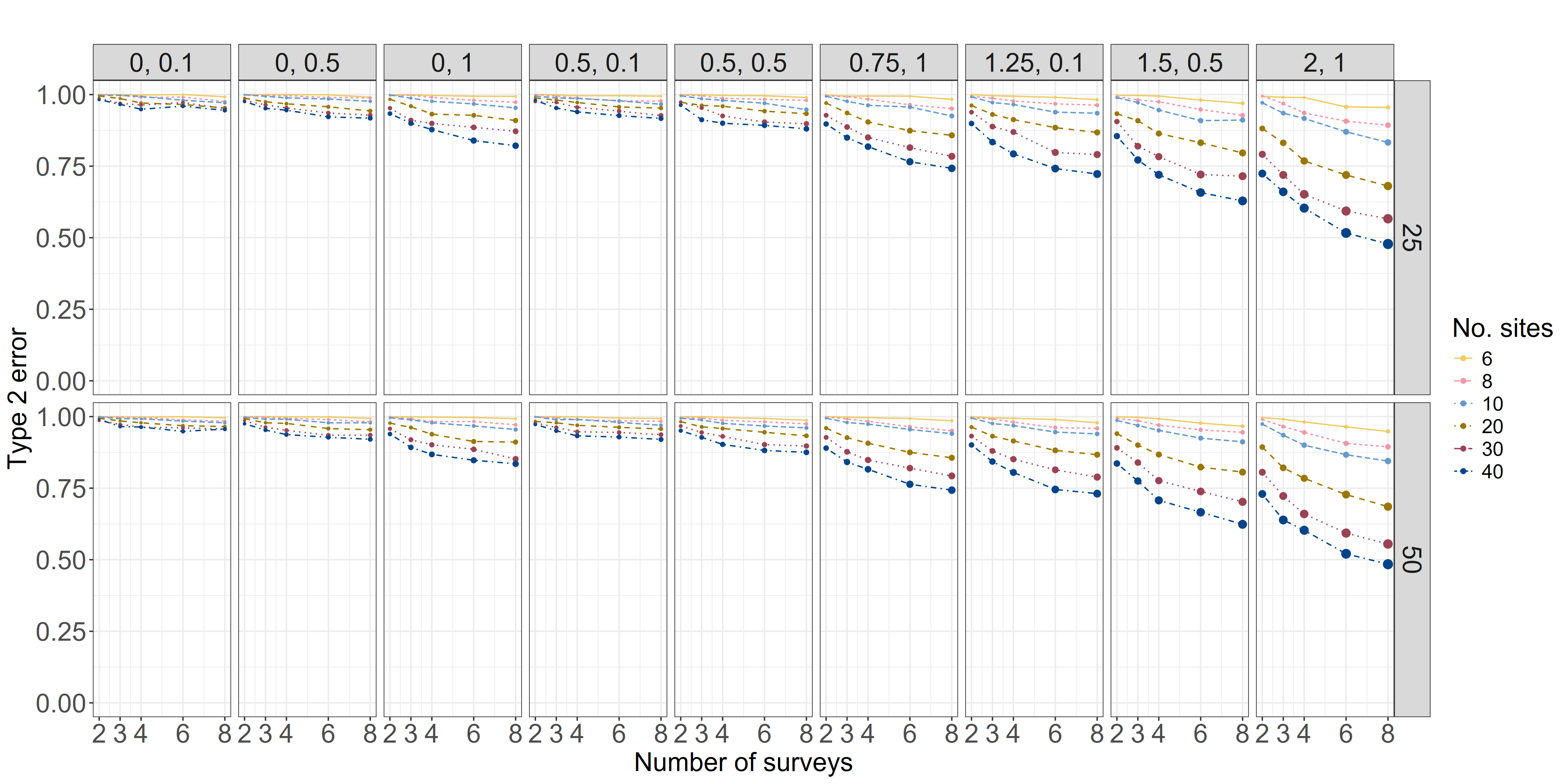


Figure 6c. Type 2 error risk under the hybrid occupancy model. Columns vary by the normal distribution parameters governing the treatment effect scenario. Rows vary by the number of species in the community. The number of species experiencing non-negligble treatment effect increased as the mean effect magnitude increased (left to right). Point sizes reflect the relative proportion of the sample experiencing some treatment effect.

### Only rare species - SSOM


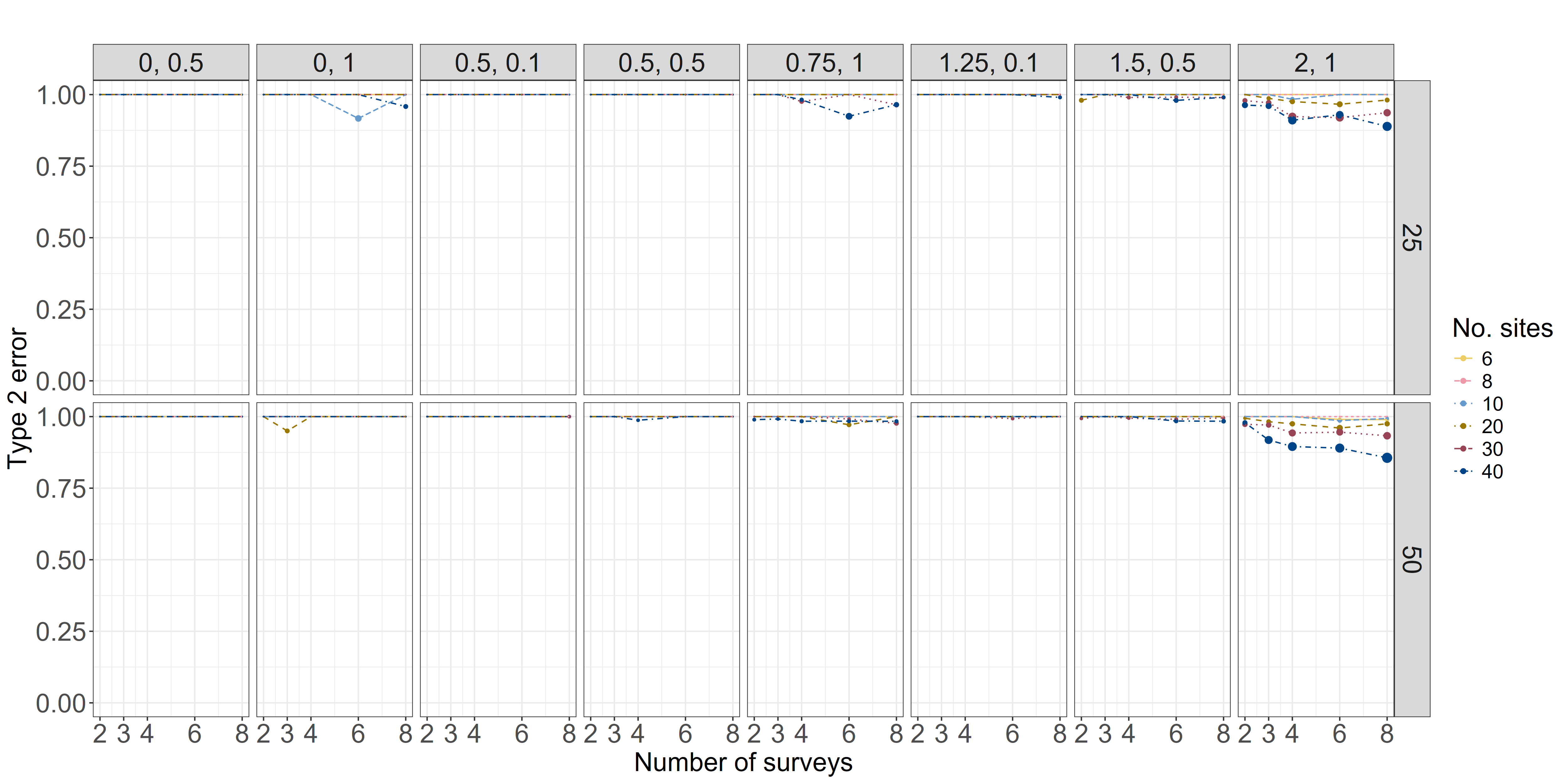


Figure 6d. Type 2 error risk under the single species occupancy model (SSOM) when only species of concern were considered. Columns vary by the normal distribution parameters governing the treatment effect scenario. Rows vary by the number of species in the community. The number of species experiencing non-negligble treatment effect increased as the mean effect magnitude increased (left to right). Point sizes reflect the relative proportion of the sample experiencing some treatment effect.

### Only rare species - MSOM


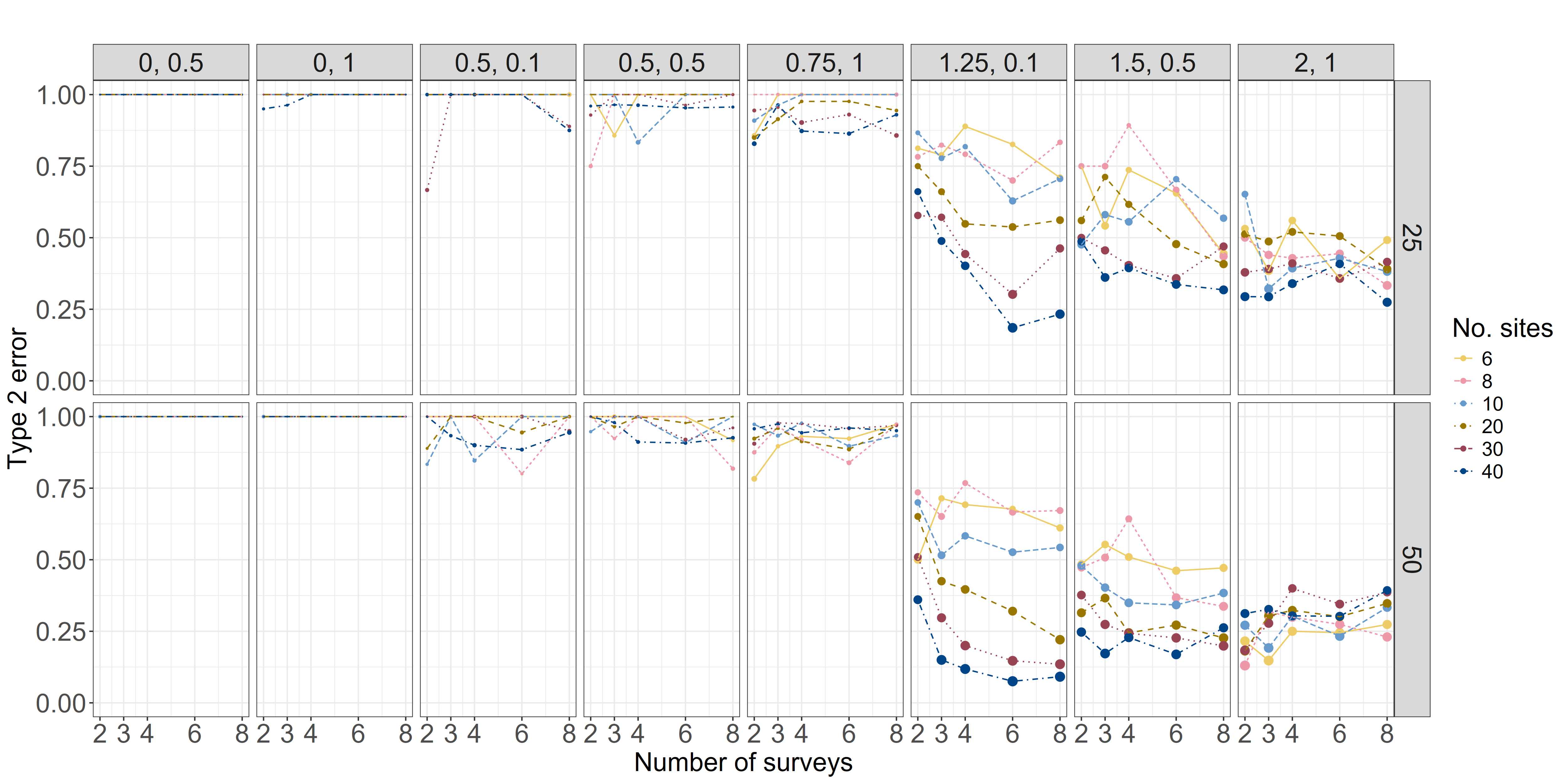


Figure 6e. Type 2 error risk under the multispecies occupancy model (MSOM) when only species of concern were considered. Columns vary by the normal distribution parameters governing the treatment effect scenario. Rows vary by the number of species in the community. The number of species experiencing non-negligble treatment effect increased as the mean effect magnitude increased (left to right). Point sizes reflect the relative proportion of the sample experiencing some treatment effect.

### Only rare species - Hybrid


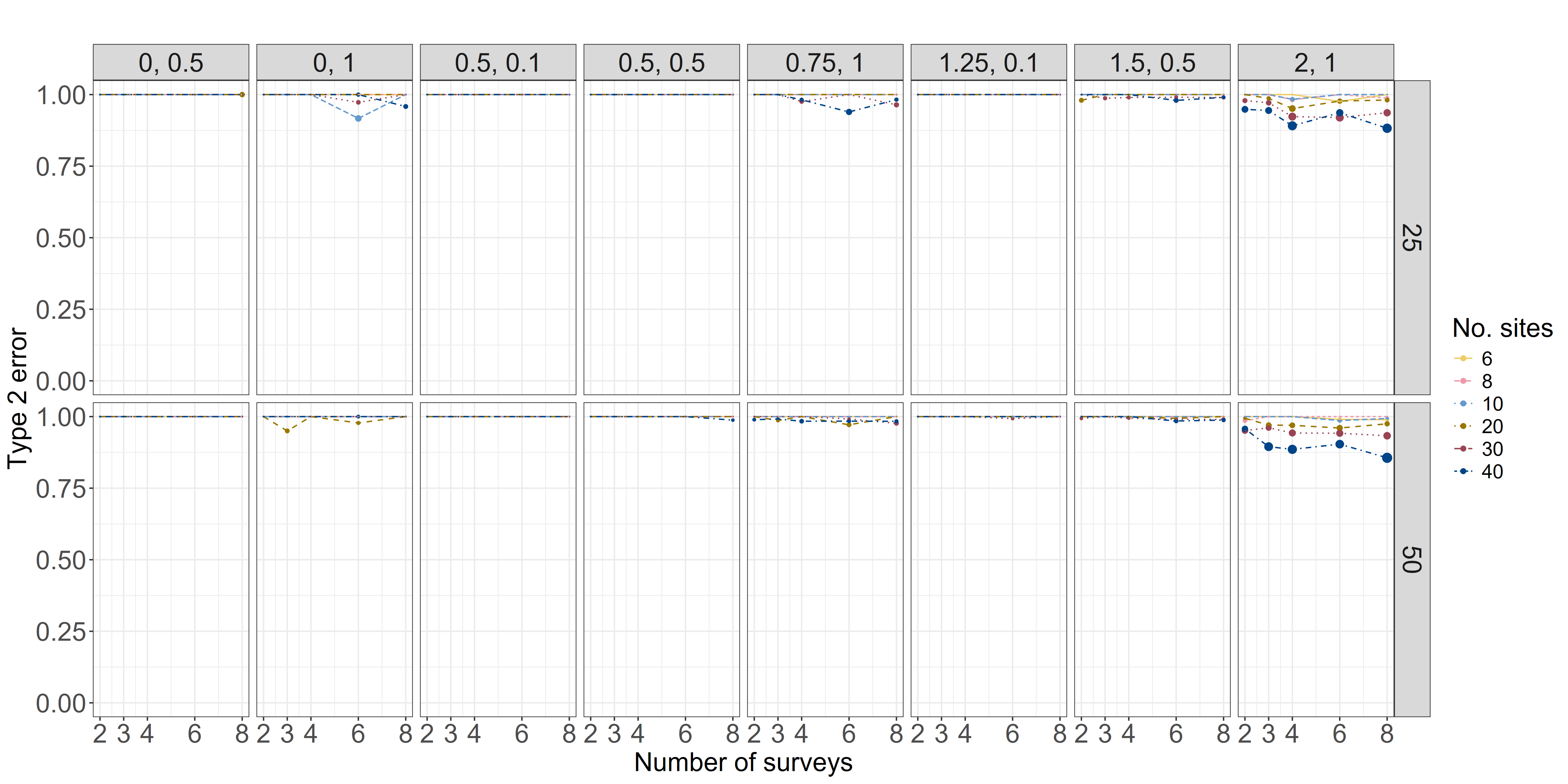


Figure 6f. Type 2 error risk under the hybrid occupancy model when only species of concern were considered. Columns vary by the normal distribution parameters governing the treatment effect scenario. Rows vary by the number of species in the community. The number of species experiencing non-negligble treatment effect increased as the mean effect magnitude increased (left to right). Point sizes reflect the relative proportion of the sample experiencing some treatment effect.

## Percent correctly classified

For each species-specific treatment effect estimate in each model run, the proportions of the posterior distributions falling on the ‘correct side’ of zero were averaged across the community and 100 iterations. More sampling improves PCC and this metric improves with large treatment effects, but can also be positively influenced by community-level estimate bias under the MSOM.

### All species - SSOM


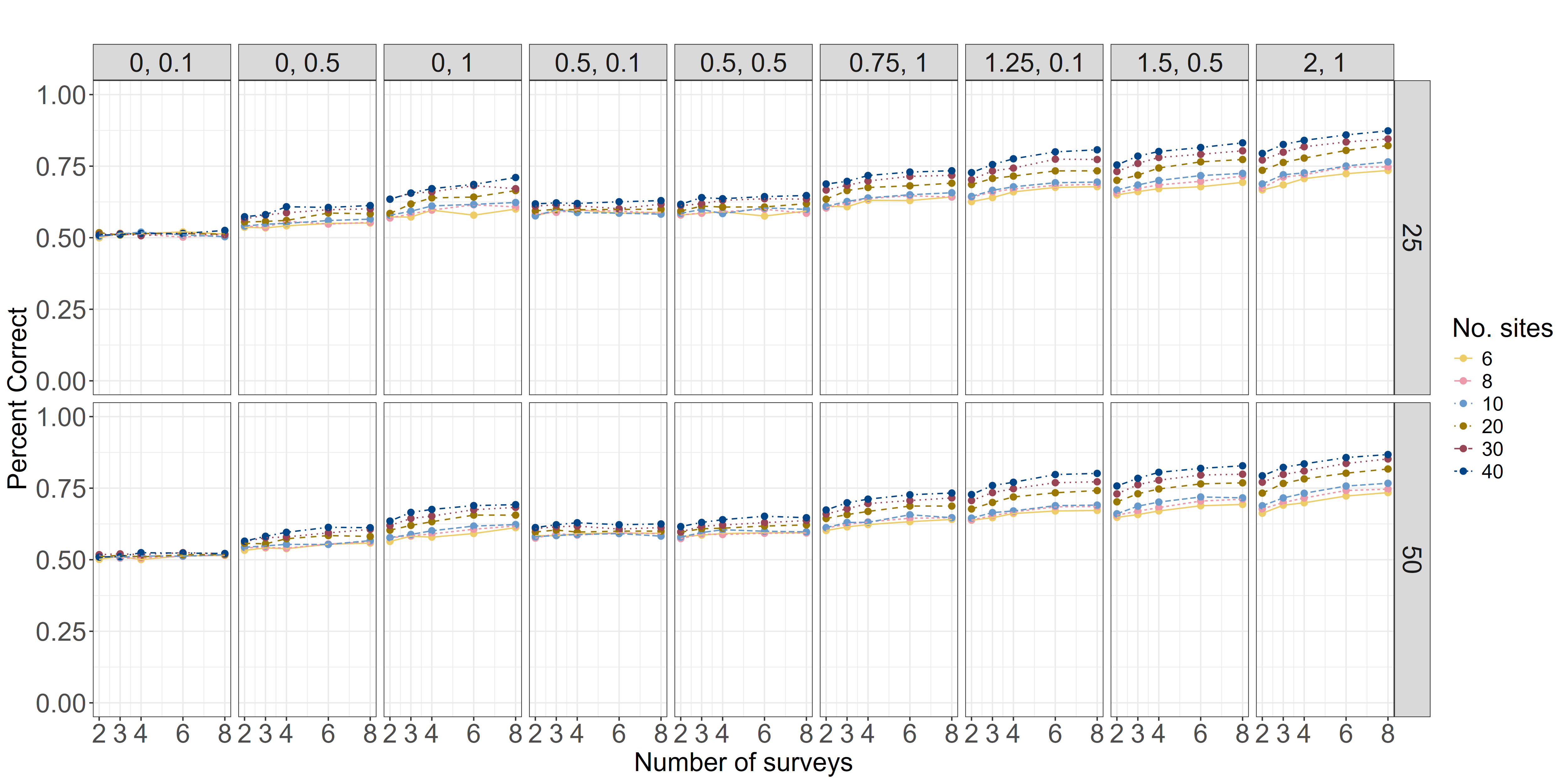


Figure 7a. Percent of correctly classified treatment effect posterior distributions under the single species occupancy model (SSOM). Columns vary by the normal distribution parameters governing the treatment effect scenario. Rows vary by the number of species in the community.

### All species - MSOM


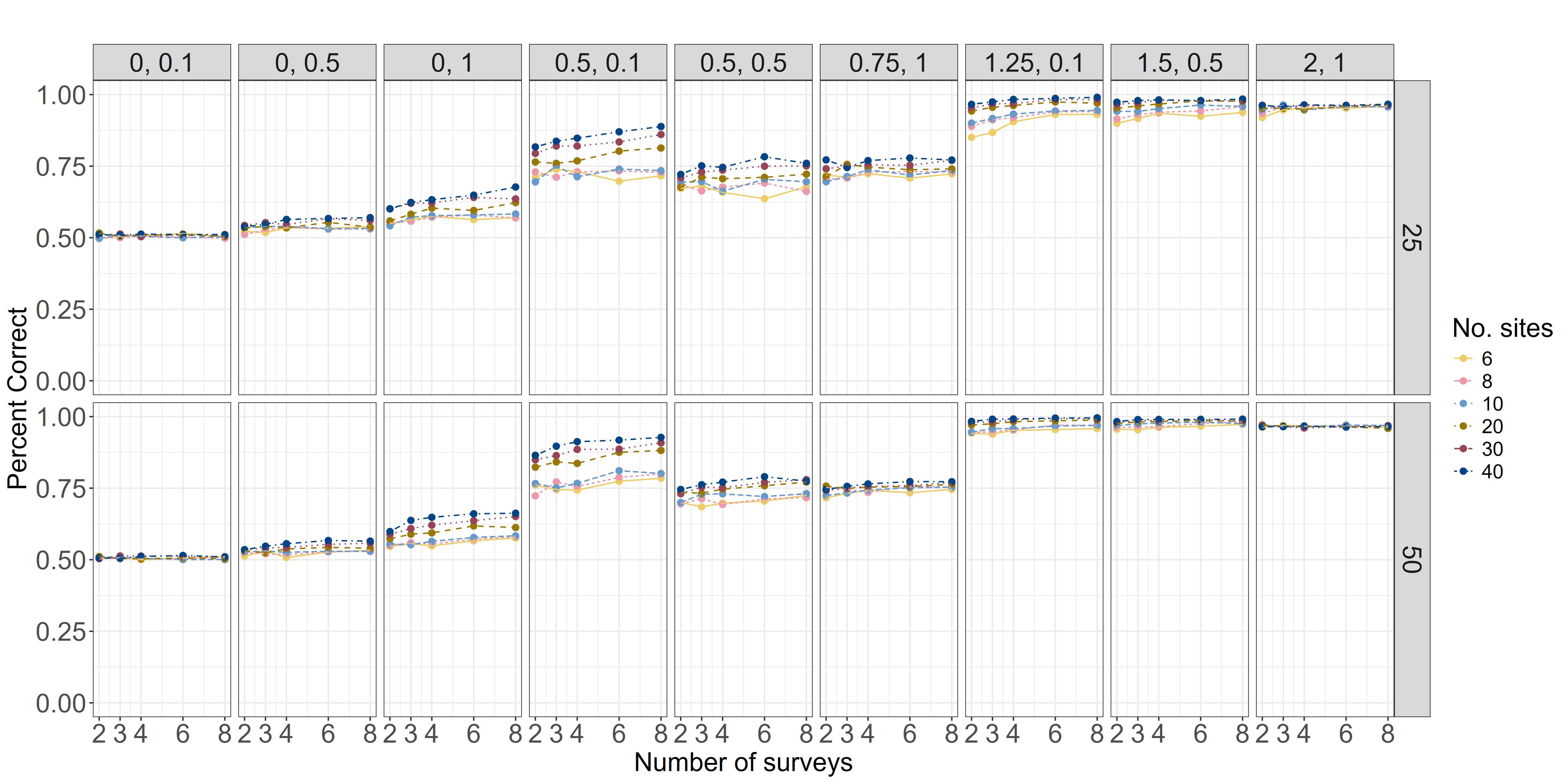


Figure 7b. Percent of correctly classified treatment effect posterior distributions under the multispecies occupancy model (MSOM). Columns vary by the normal distribution parameters governing the treatment effect scenario. Rows vary by the number of species in the community.

### All species - Hybrid


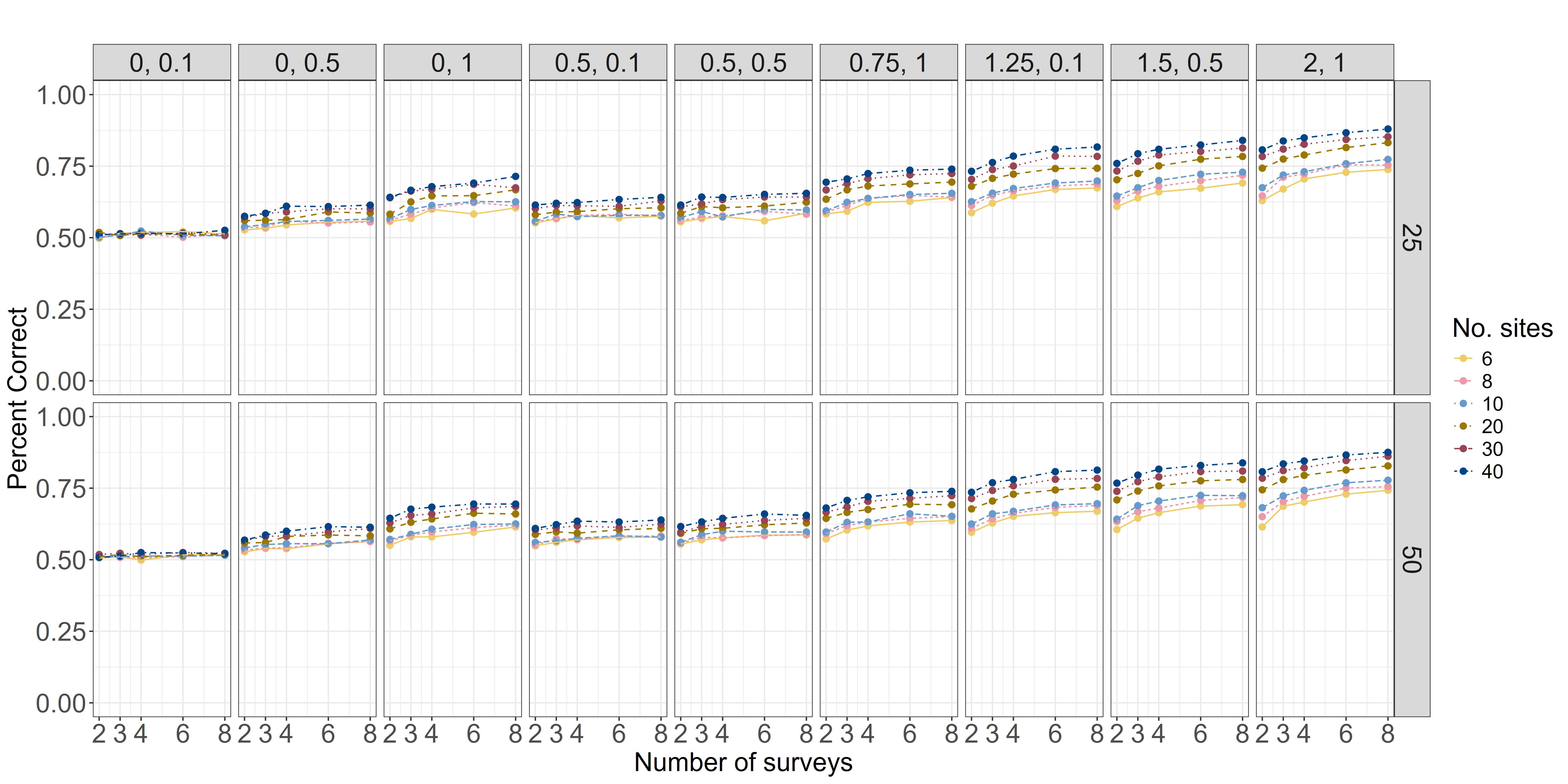


Figure 7c. Percent of correctly classified treatment effect posterior distributions under the hybrid occupancy model. Columns vary by the normal distribution parameters governing the treatment effect scenario. Rows vary by the number of species in the community.

### Only rare species - SSOM


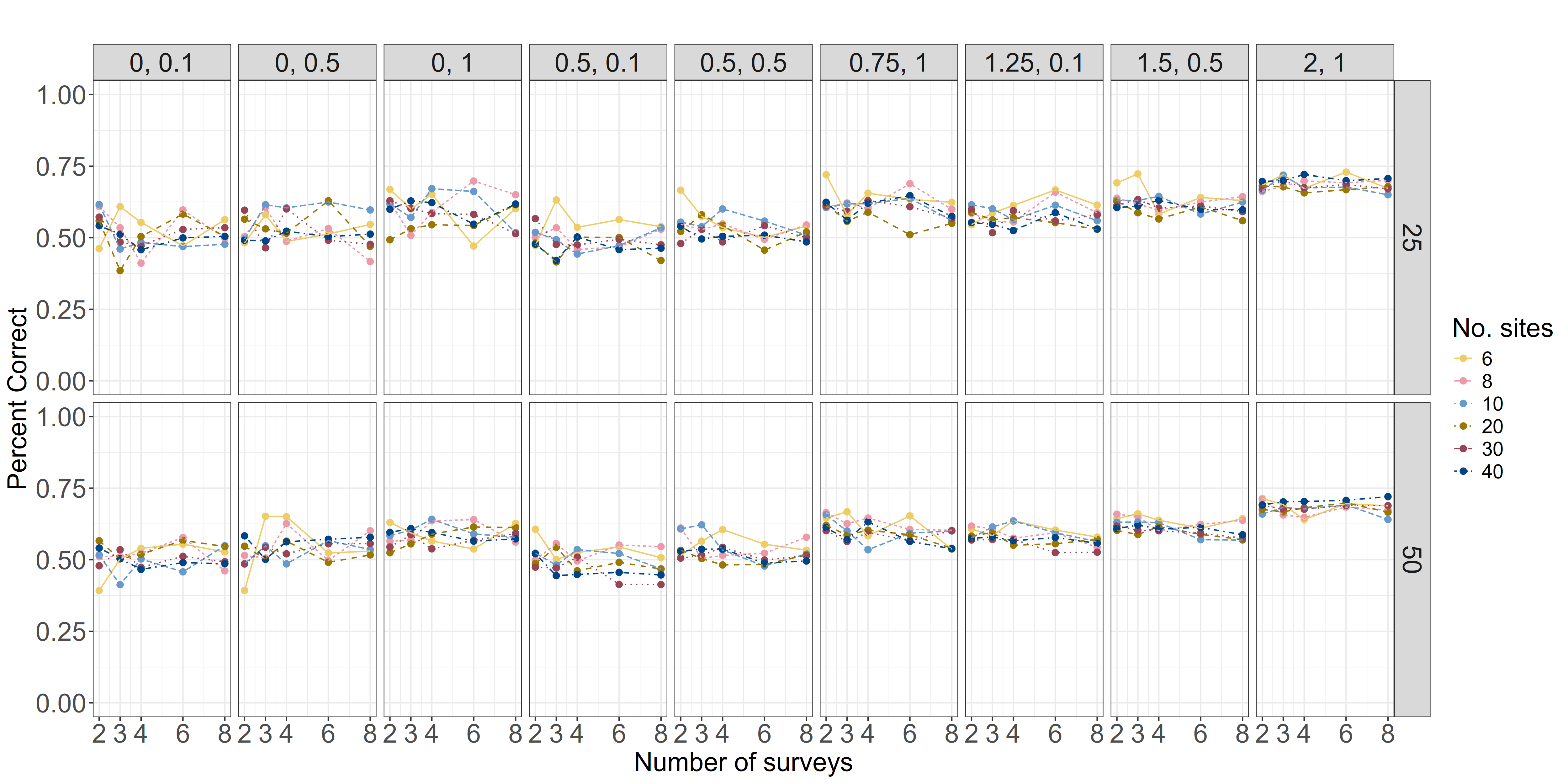


Figure 7d. Percent of correctly classified treatment effect posterior distributions under the single species occupancy model (SSOM) when only species of concern were considered. Columns vary by the normal distribution parameters governing the treatment effect scenario. Rows vary by the number of species in the community.

### Only rare species - MSOM


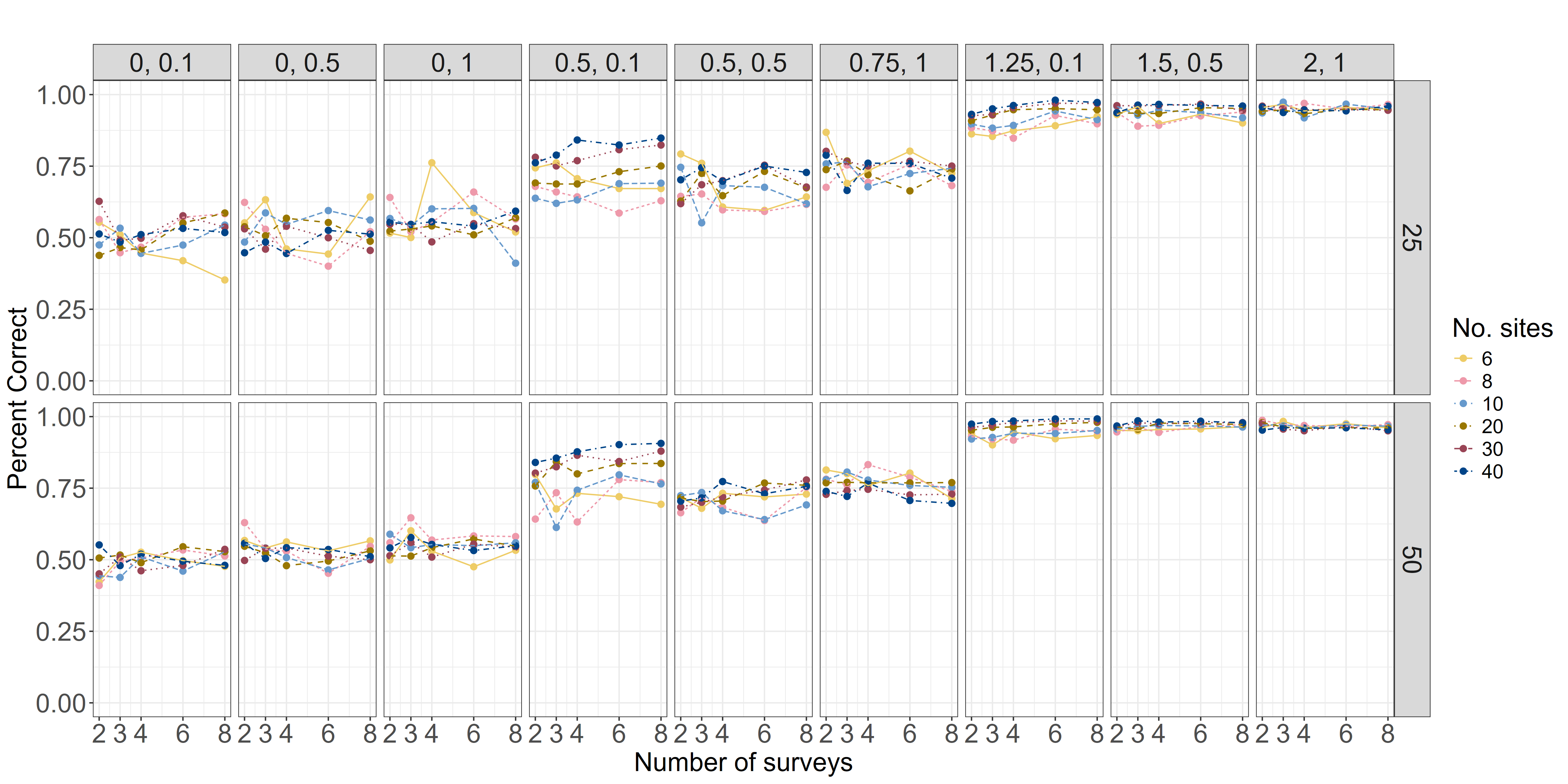


Figure 7e. Percent of correctly classified treatment effect posterior distributions under the multispecies occupancy model (MSOM) when only species of concern were considered. Columns vary by the normal distribution parameters governing the treatment effect scenario. Rows vary by the number of species in the community.

### Only rare species - Hybrid


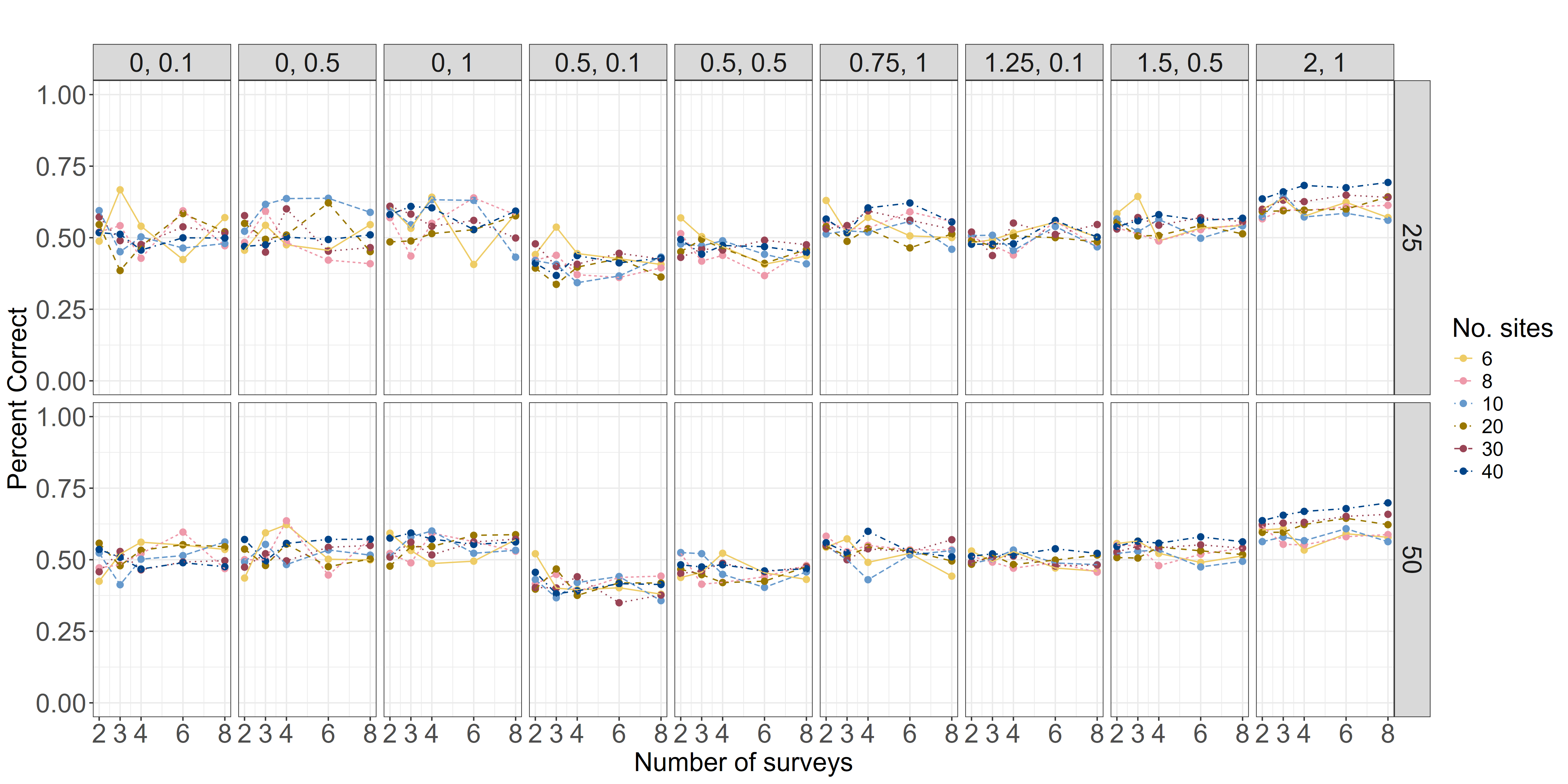


Figure 7f. Percent of correctly classified treatment effect posterior distributions under the hybrid occupancy model when only species of concern were considered. Columns vary by the normal distribution parameters governing the treatment effect scenario. Rows vary by the number of species in the community.

# Disclaimer

Disclaimer: Any use of trade, firm, or product names is for descriptive purposes only and does not imply endorsement by the U.S. Government.
